# Supplementary material for: Osh Proteins Control Nanoscale Lipid Organization Necessary for PI(4,5)P2 Synthesis
Source: Mol Cell. 2019 Sep 5;75(5):1043–1057.e8. doi: 10.1016/j.molcel.2019.06.037 (PMC6739424; doi:10.1016/j.molcel.2019.06.037)
Supplement: Document S1. Figures S1–S7 [file mmc1.pdf]

**Molecular Cell, Volume *75***

## Supplemental Information

# Osh Proteins Control Nanoscale Lipid Organization Necessary for $PI(4,5)P_2$ Synthesis

**Taki Nishimura, Michael Gecht, Roberto Covino, Gerhard Hummer, Michal A. Surma, Christian Klose, Hiroyuki Arai, Nozomu Kono, and Christopher J. Stefan**

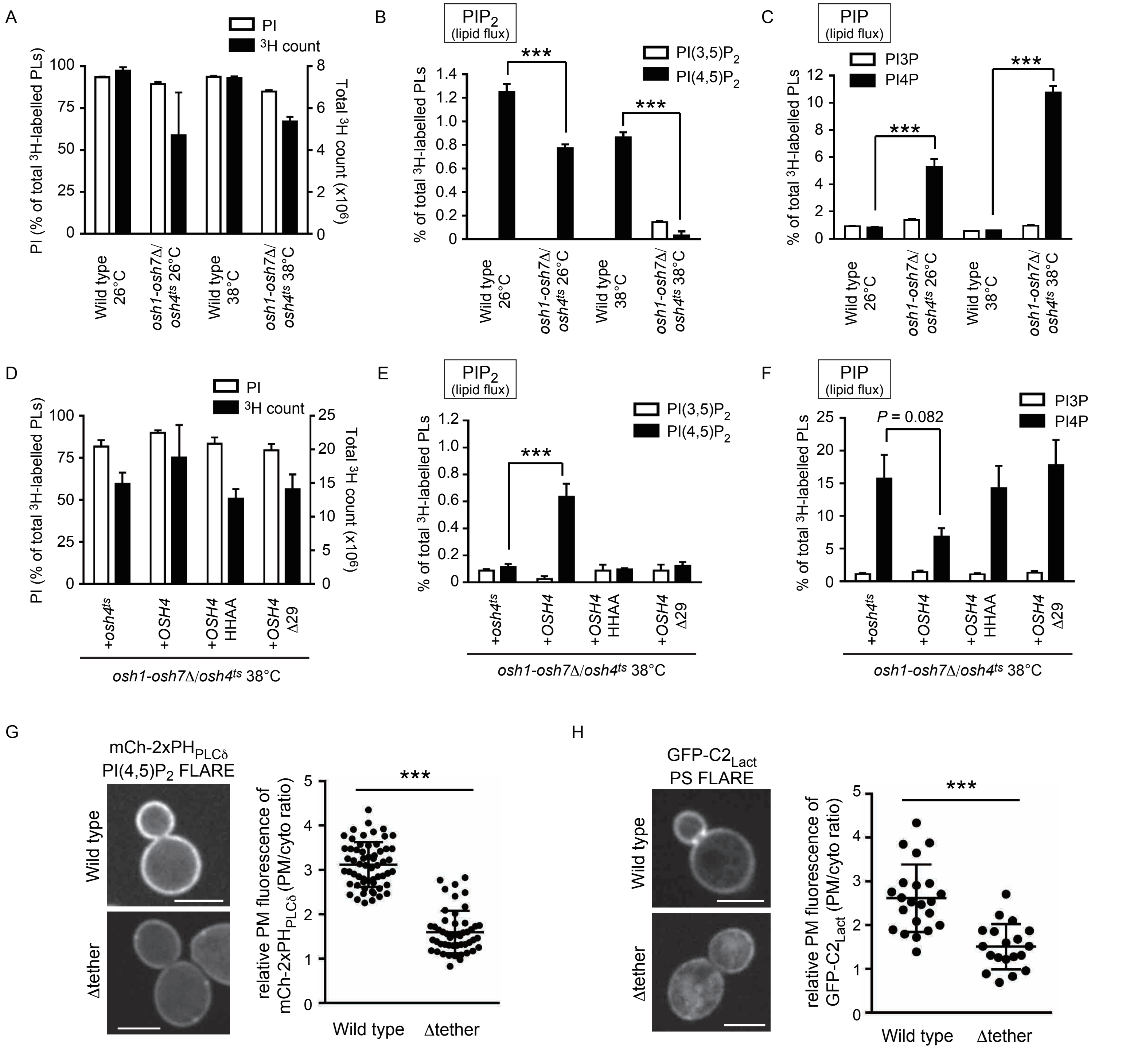


**Figure S1. Reduction of PI(4,5)P$_2$ and PS levels at the PM of *osh1-7Δ/osh4$^{ts}$* and Δtether mutants, related to Figure 1 and the Supplemental Data Set**

(A-C) Measurements of phosphatidylinositol (PI) and phosphoinositide lipid metabolism by $^3$H-inositol labeling and HPLC analysis of wild type and *osh1-7Δ/osh4$^{ts}$* cells cultured at 26ºC or 38ºC for 1 h. Data represent mean ± SEM (n = 3). (D-F) Measurements of phosphatidylinositol (PI) and phosphoinositide lipid metabolism by $^3$H-inositol labeling and HPLC analysis of *osh1-7Δ/osh4$^{ts}$* cells expressing wild type or mutant forms of Osh4 cultured at 38ºC for 1 h. Data represent mean ± SEM (n = 3). (G, H) PI(4,5)P$_2$ (mCherry-2xPH$_{PLCδ}$) and PS (GFP-C2$_{Lact}$) FLARE localization in wild type and Δtether cells. Data represent mean ± SD; (G) n≧50 cells, (H) n≧19 cells. Scale bars, 4 µm. ****P* < 0.001.

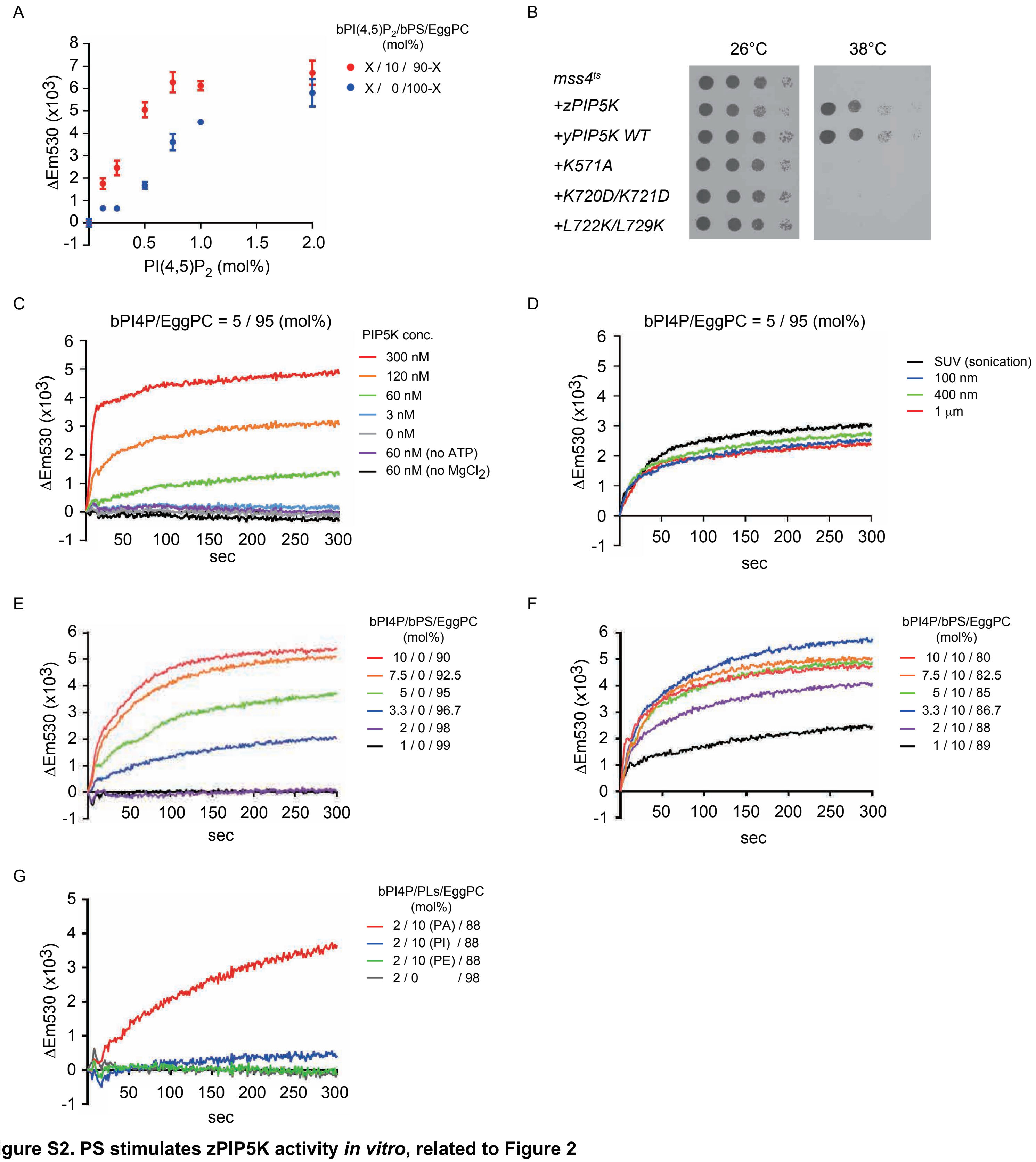


**Figure S2. PS stimulates zPIP5K activity *in vitro*, related to Figure 2**

(A) Changes in NBD-$PH_{PLC\delta}$ fluorescence intensity in response to $PI(4,5)P_2$ levels on liposomes. Liposomes were incubated with 400 nM NBD-$PH_{PLC\delta}$ for 5 min and NBD fluorescence was recorded by fluorescence spectroscopy. Data represent mean ± SEM (n=3). (B) Growth assay of *mss4$^{ts}$* mutant cells. A zebrafish PIP5K catalytic domain (zPIP5K), a full-length yPIP5K wild-type (yPIP5K WT), a kinase dead mutant (K571A), a negatively charged mutant (K720D/K721D), an amphipathic property mutant (L722K/L729K) or empty pRS416 plasmid was expressed in *mss4$^{ts}$* mutant cells as indicated. Serial dilutions of the cells were grown on –Ura plates to retain the plasmids at either 26ºC or 38ºC for 4 days. (C) Increase of NBD-$PH_{PLC\delta}$ fluorescence intensity reflects $PI(4,5)P_2$ generation by the PIP 5-kinase (PIP5K) reaction. Real-time PIP5K assays were performed as described in the STAR Methods. Note that zPIP5K activity was undetectable in the absence of zPIP5K, ATP, and $MgCl_2$. Data represent mean values (n=3; SEM<$0.36x10^3$). (D) zPIP5K showed minimal sensitivity to differences in size of liposomes containing 5 mol% bPI4P. The indicated sizes of liposomes were obtained by extrusion. Small unilamellar vesicles (SUV) were prepared by sonication. Data represent mean values (n≧3; SEM<$0.44x10^3$). (E, F) PIP5K activity against liposomes containing different concentrations of brain PI4P. Liposomes containing 0 mol% bPS (E) or 10 mol% bPS (F) were analyzed. (E, F) Data represent mean values (n≧3; SEM<$1.07x10^3$). (G) The effect of phosphatidic acid (PA), phosphatidylinositol (PI) and phosphatidylethanolamine (PE) on PIP5K activity in a real-time PIP5K assay. Data represent mean values (n=3; SEM<$0.64x10^3$).

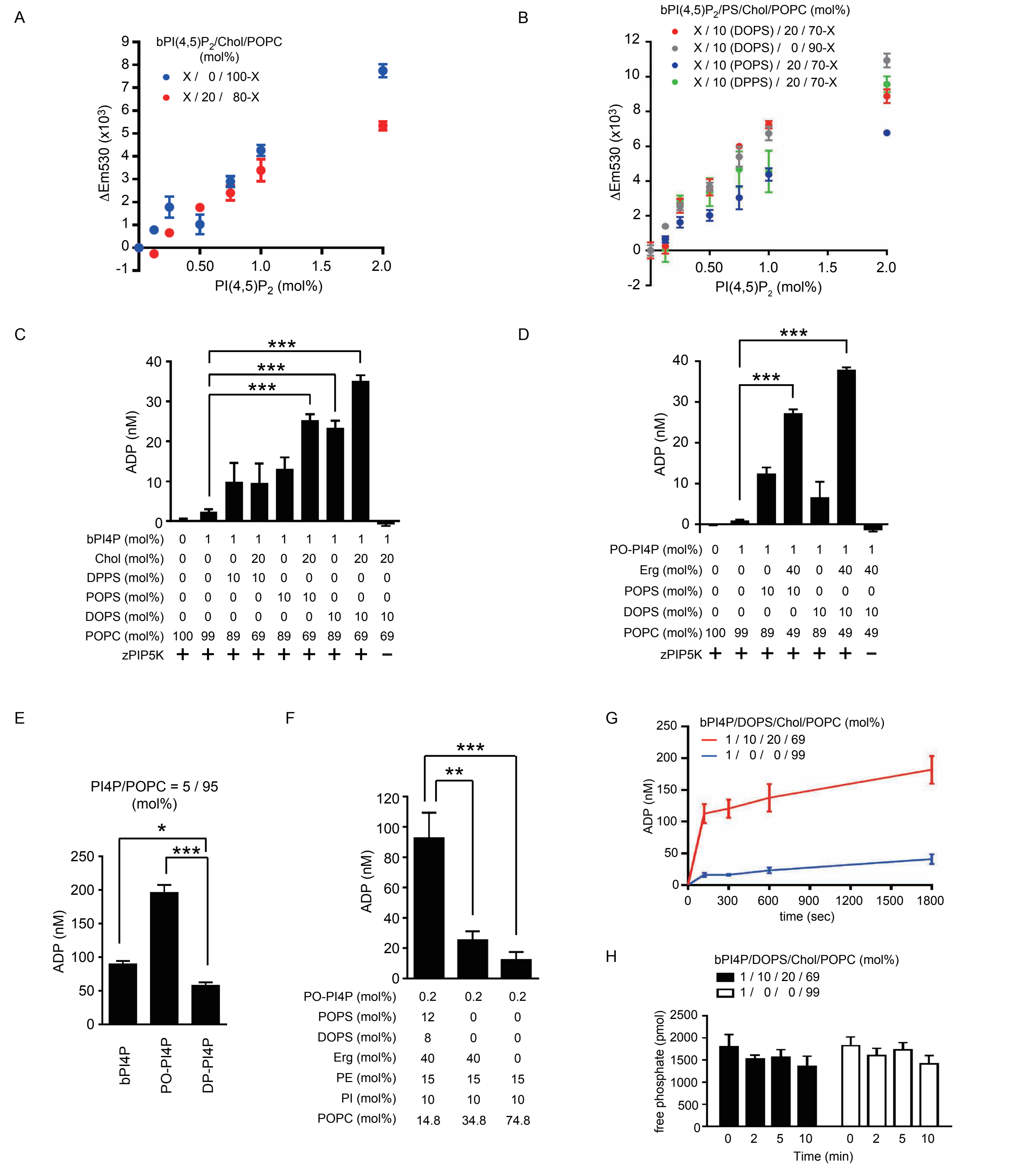


**Figure S3. Sterols stimulate zPIP5K activity *in vitro,* related to Figure 3**

(A) Effects of cholesterol on $PI(4,5)P_2$-dose dependency of NBD-$PH_{PLC\delta}$ fluorescence intensity. Data represent mean ± SEM (n=3).
(B) Calibration of effects by PS and cholesterol on NBD-$PH_{PLC\delta}$ sensitivity to $PI(4,5)P_2$. Data represent mean ± SEM (n=3).
(C) Stimulation of PIP5K activity by phosphatidylserine (PS) and cholesterol. Liposomes containing the indicated PS species and/or cholesterol were used as a substrate. After 3 min, the PIP5K reactions were stopped with ADP-Glo reagent. Data represent mean ± SEM (n = 3).
(D) Unsaturated PS and ergosterol (Erg) stimulate PIP5K activity. Liposomes containing various PS species and/or ergosterol as indicated were used as a substrate. After 3 min, the PIP5K reactions were stopped with ADP-Glo reagent. Data represent mean ± SEM (n = 3).
(E) The effect of PI4P unsaturation on zPIP5K activity. After 2 min, the PIP5K reactions were stopped with ADP-Glo reagent. Data represent mean ± SEM (n = 3). (F) ADP-Glo assay using PIP5K and liposomes resembling yeast PM lipid composition (Zinser et al., 1991) or in the absence of PS and ergosterol as indicated. After 2 min, the PIP5K reactions were stopped with ADP-Glo reagent. Data represent mean ± SEM (n = 3). (G) A time course analysis of PIP5K activity using the ADP-Glo assay. PIP5K (final conc. 130 nM) was used. Note that ADP levels rise suddenly after 120 sec in the presence of DOPS and cholesterol. Data represent mean ± SEM (n=3). (H) Free phosphate levels during the indicated PIP5K reactions were monitored over time by the Malachite Green Phosphate assay. Data represent mean ± SEM (n=3).
$^{*}P < 0.05$, $^{**}P < 0.01$, $^{***}P < 0.001$.

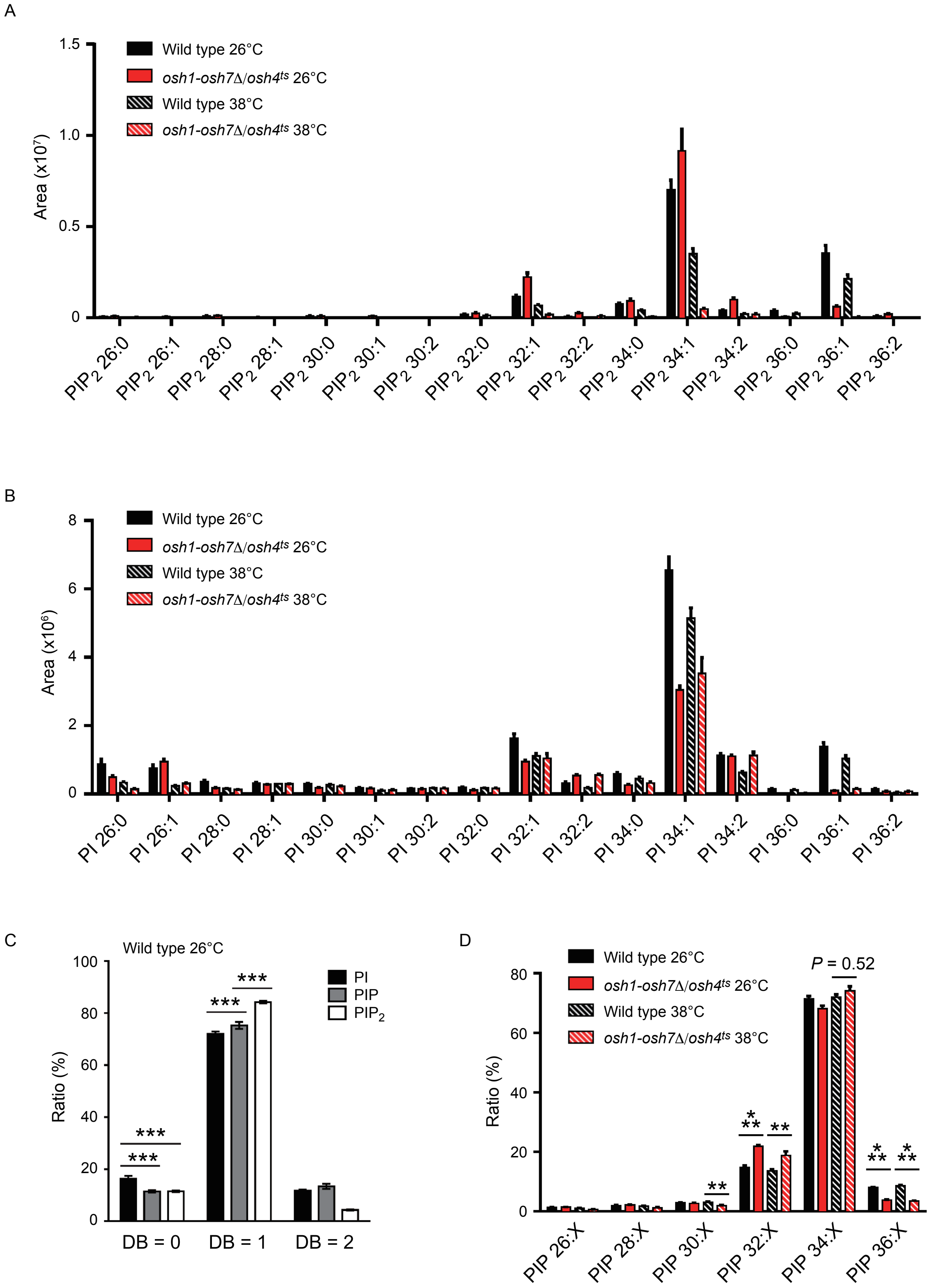


**Figure S4 The fatty acid compositions of PI, PIP and $PIP_2$ in wild type and *osh1-7Δ/osh4$^{ts}$* mutant cells, related to Figure 4**
Analysis of PI, PIP, and $PIP_2$ levels in wild-type and *osh1-7Δ/osh4$^{ts}$* cells cultured at 26ºC or 38ºC for 2 h by quantitative lipidomics was performed as described in the STAR Methods. (A, B) Fatty acid compositions of $PIP_2$ and PI. (C) Mono-unsaturated $PIP_2$ species are enriched in wild-type cells. (D) Fatty acid length of PIP species. (A-D) Data represent mean ± SEM (n = 5). ***P* < 0.01, ****P* < 0.001.

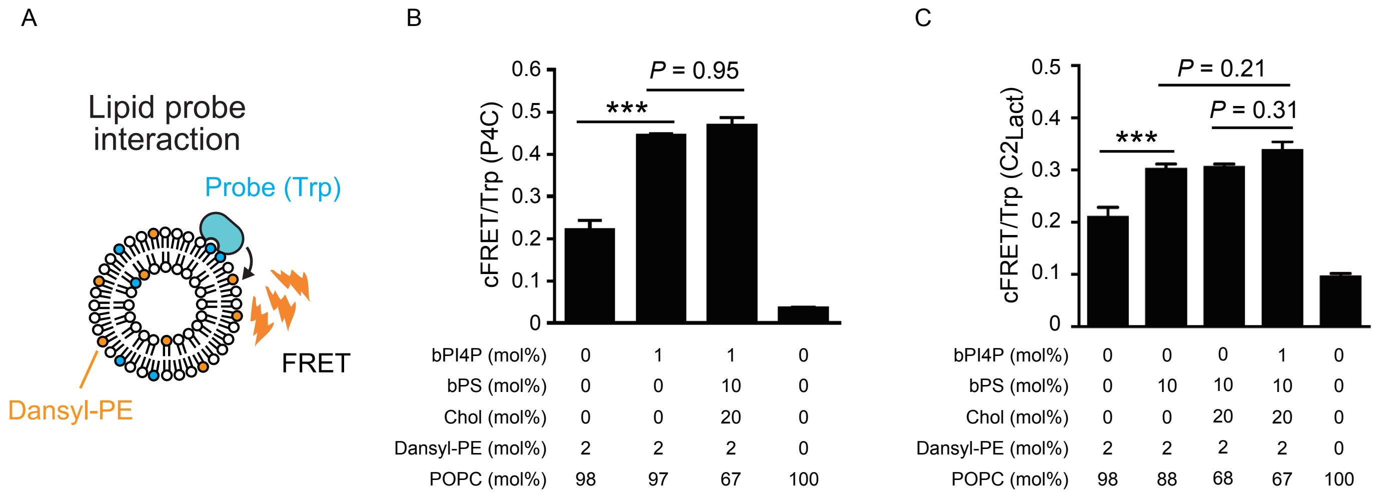


**Figure S5. Quantitative measurements of lipid probe binding in the presence of unsaturated PS, PI4P, and cholesterol, related to Figure 5**

(A) Membrane binding assay using Dansyl-PE liposomes. FRET signals increase by the proximity of dansyl-PE in the liposome to tryptophan residues in lipid probes. Corrected FRET (cFRET) was calculated as described in STAR Methods.

(B) Quantification of the PI4P probe (P4C) binding to dansyl-PE liposomes of defined compositions. (C) Quantification of PS probe ($C2_{Lact}$) binding to dansyl-PE liposomes as indicated. Data represent the mean ± SEM (n=3). ***$P$ < 0.001.

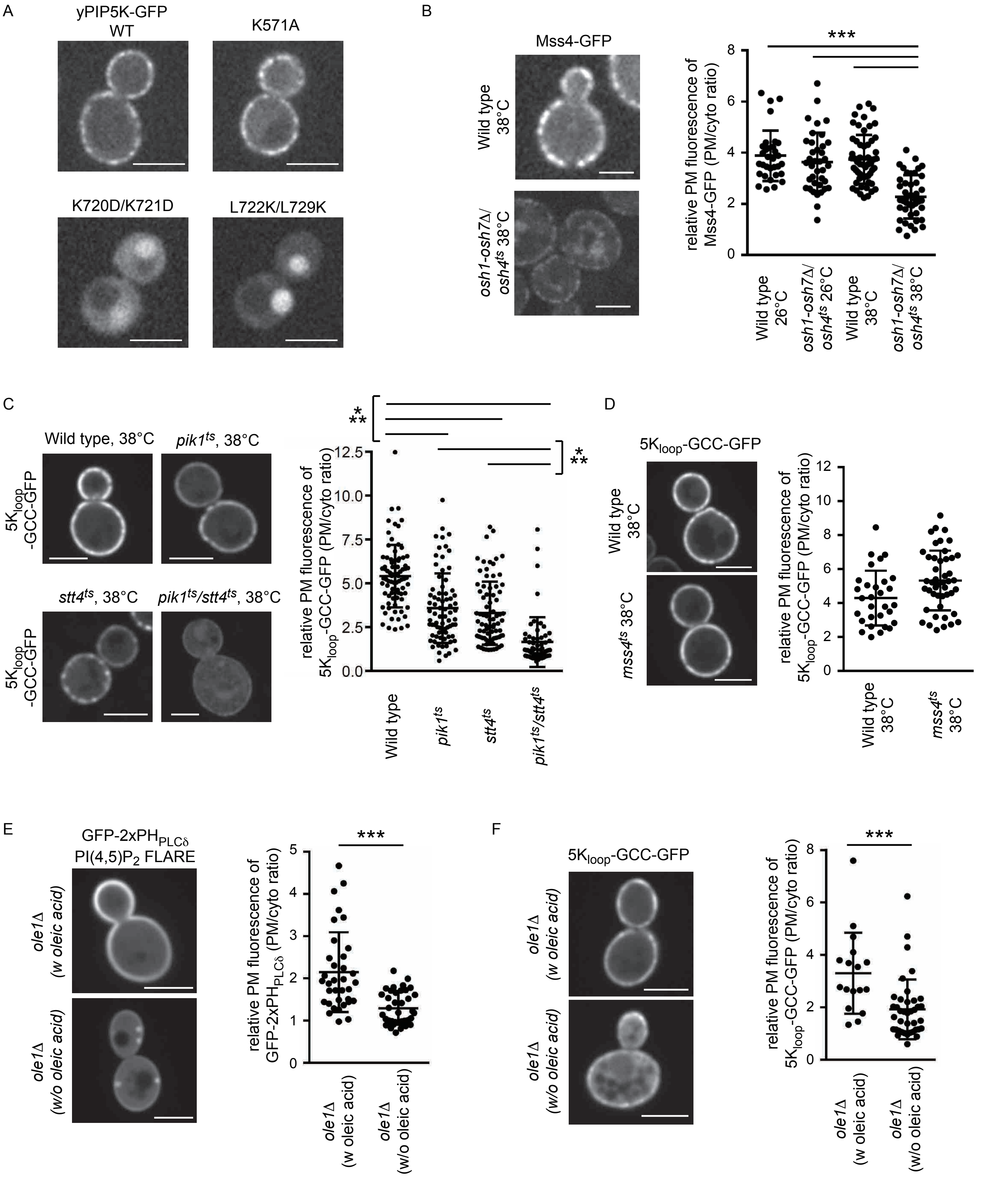


**Figure S6. Unsaturated PI4P and PS co-distribution in the PM is recognized by the specificity loop of PIP5K, related to Figure 6**

(A) Localization of GFP-tagged wild type and mutant yeast PIP5K Mss4 proteins (yPIP5K-GFP) in *mss4*$^{ts}$ mutant cultured at 26ºC. (B) Mss4-GFP localization in wild type and *osh1-7Δ/osh4*$^{ts}$ cells cultured at 38ºC for 2 h (left panels). Quantification of Mss4-GFP localization (PM/cyto ratio) in wild type and *osh1-7Δ/osh4*$^{ts}$ cells cultured at 26ºC or 38ºC for 2 h (right panel). Data represent mean ± SD (n≧32 cells). (C) Localization of $5K_{loop}$-GCC-GFP in wild-type, *pik1*$^{ts}$, *stt4*$^{ts}$ and *pik1*$^{ts}$*/stt4*$^{ts}$ mutants cultured at 38ºC for 1 h. (left panels). Quantification of $5K_{loop}$-GCC-GFP signals in the PM of wild-type, *pik1*$^{ts}$, *stt4*$^{ts}$ and *pik1*$^{ts}$*/stt4*$^{ts}$ mutants. Data represent mean ± SD (n≧61 cells). (D) Quantitative analysis of $5K_{loop}$-GCC-GFP PM localization in wild type and *mss4*$^{ts}$ mutant cells cultured at 38ºC for 1 h. Data represent mean ± SD (n≧30 cells). (E) Quantitative localization of GFP-2x$PH_{PLC\delta}$ in *ole1Δ* mutant cells cultured with or without 1 mM oleic acid for 20 h. Data represent mean ± SD (n≧35 cells). (F) Quantitative localization of $5K_{loop}$-GCC-GFP in *ole1Δ* mutant cells cultured with or without 1 mM oleic acid for 20 h. Data represent mean ± SD (n≧17 cells). Scale bars, 4 μm. ***$P < 0.001$.

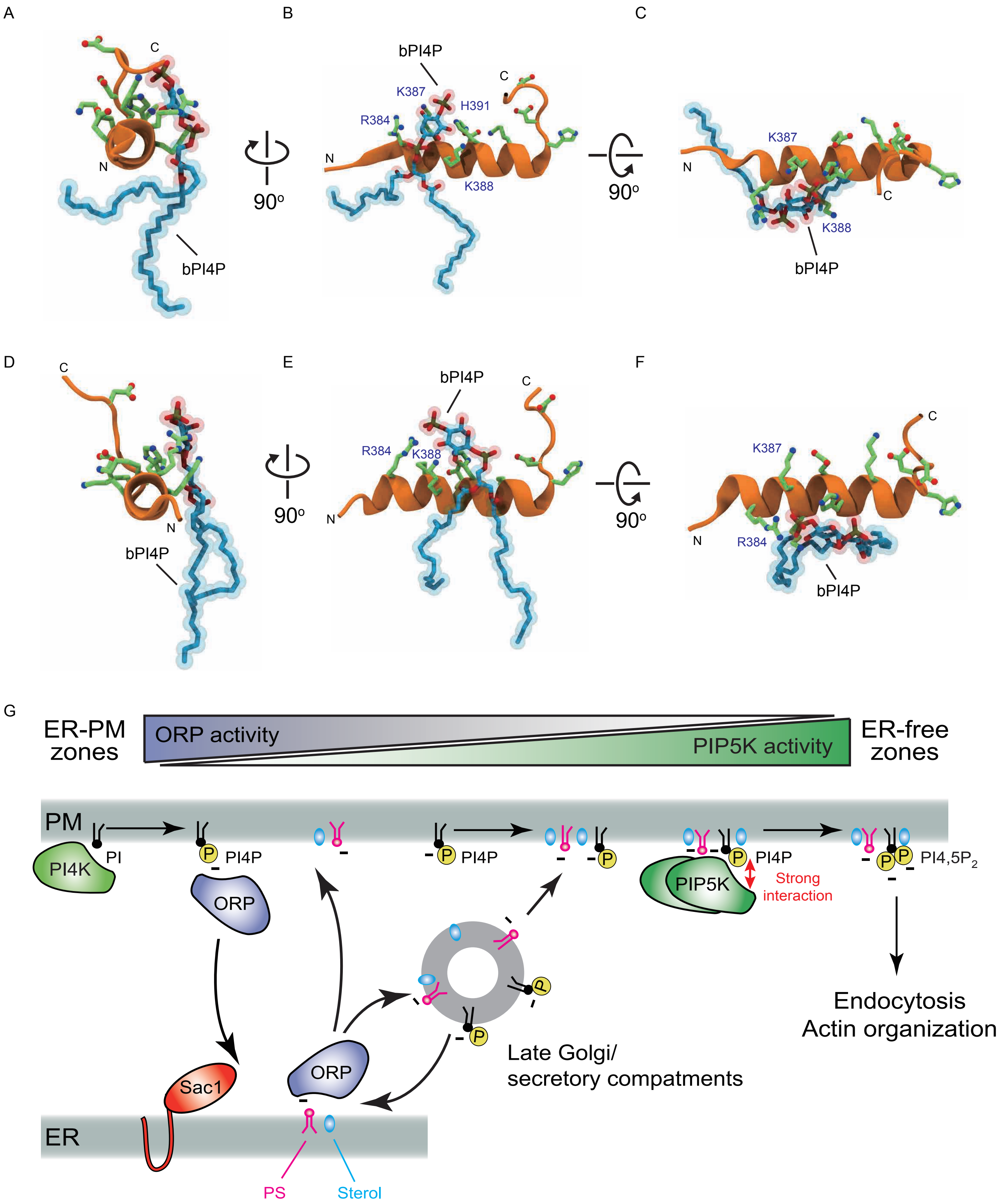


**Figure S7. Speculative model for PIP5K stimulation by Osh-dependent lipid exchange, related to Figure 7**
(A-F) The $5K_{loop}$ is visualized as a ribbon (orange), acidic and basic residues as green sticks, colored the same as in Fig 7H-J.
(A-C) The bPI4P molecule is located at pocket #1 (Fig. 7G) and interacts with four residues of the $5K_{loop}$: R384, K387, K388, and H391.
(D-F) The bPI4P molecule is located at pocket #2 (Fig. 7G) and interacts with R384 and K388. (G) Speculative model for spatial and temporal PIP5K regulation by Osh-dependent lipid transfer. PM-localized PI4KIII$\alpha$ synthesizes PI4P at the PM. As PI4P alone may not be sufficient for PIP5K activity, PI4P is used for Osh-dependent lipid exchange at ER-PM zones to facilitate PS and sterol transport from the ER to the PM. Once PS and sterol lipids reach sufficient levels, PI4P utilization switches from PI4P exchange by the Osh/Sac1 system to PI(4,5)$P_2$ synthesis by PIP5K. In addition, PI4P generated by Golgi-localized PI4K isoforms may be used for Osh-mediated PS and sterol transport to late Golgi/secretory compartments. These PS and sterol molecules are then delivered to the PM via vesicular transport for subsequent PIP5K activity. PI(4,5)$P_2$ synthesized by PIP5K controls global PM organization and dynamic events including endocytosis and actin organization in ER-free PM zones.
