# Supplementary material for: Osh Proteins Control Nanoscale Lipid Organization Necessary for PI(4,5)P2 Synthesis
Source: Mol Cell. 2019 Sep 5;75(5):1043–1057.e8. doi: 10.1016/j.molcel.2019.06.037 (PMC6739424; doi:10.1016/j.molcel.2019.06.037)
Supplement: Data S1. Dataset of the Glycerolipid Composition of Wild-Type, osh1-7Δ/osh4ts, and Δtether Yeast Cells (Shown as Mean mol% ± SEM, n = 3), Related to Figures 1, S1, 4, and S4 [file mmc2.pdf]

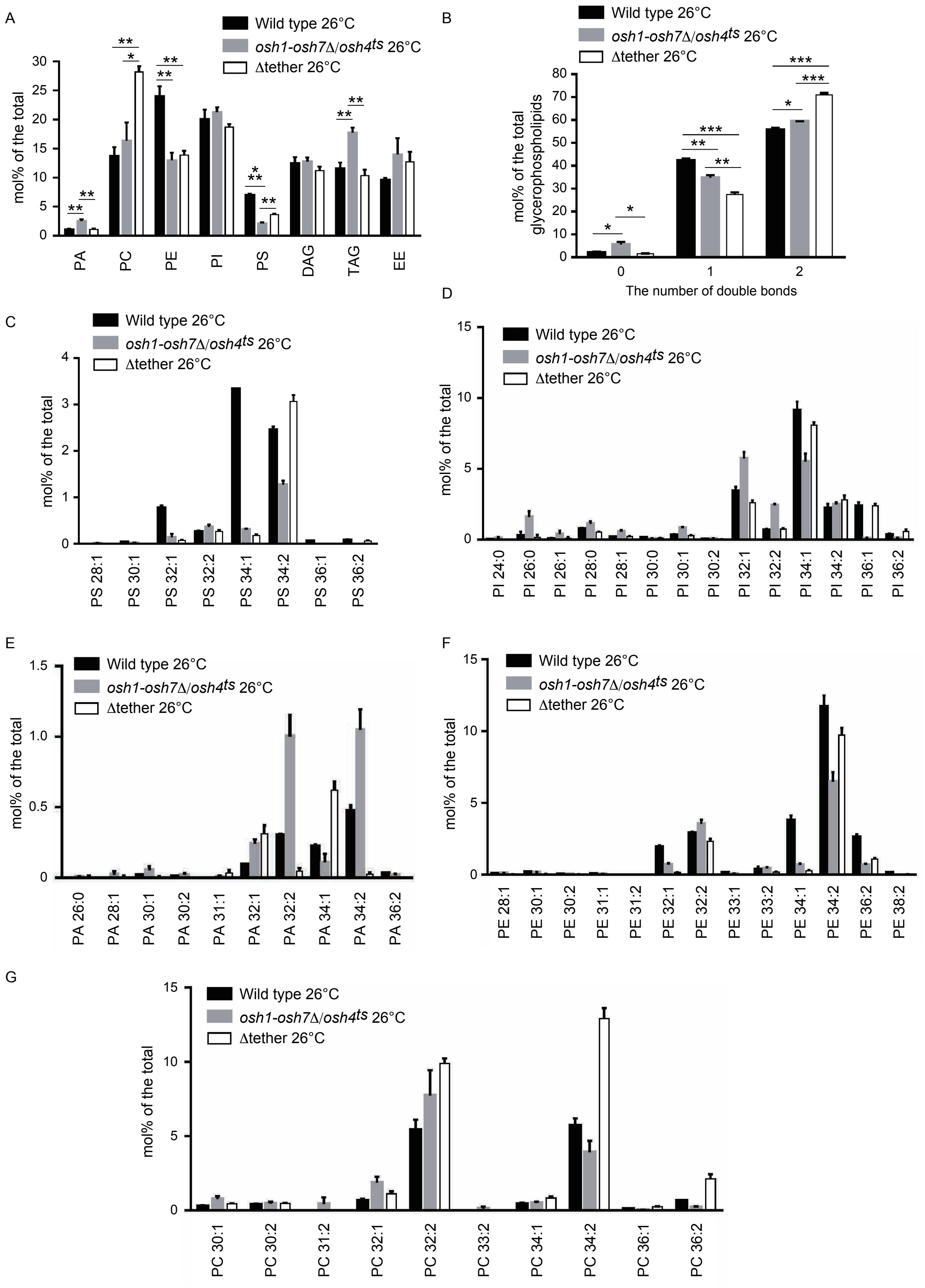

Supplemental Data Set, related to Figures 1, S1, 4, and S4. Data set of the glycerolipid composition of wild type, *osh1-7Δ/osh4^{ts}* and Δtether yeast cells (shown as mean mol% ± SEM, n=3).

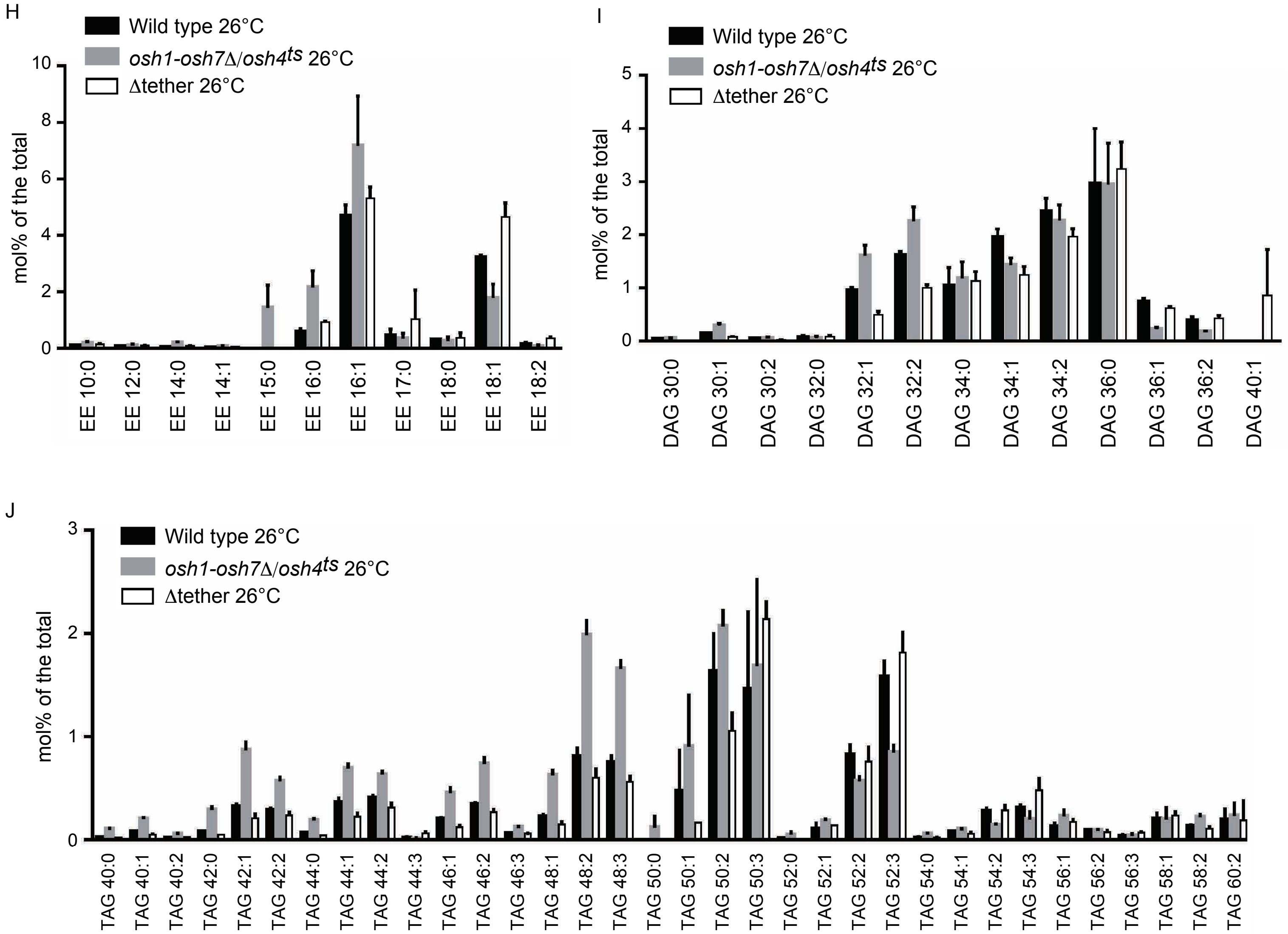

Supplemental Data Set, related to Figures 1, S1, 4, and S4. Data set of the glycerolipid composition of wild type, *osh1-7*Δ/*osh4*^{ts} and Δtether yeast cells (shown as mean mol% ± SEM, n=3).
