# Supplementary material for: Osh Proteins Control Nanoscale Lipid Organization Necessary for PI(4,5)P2 Synthesis
Source: Mol Cell. 2019 Sep 5;75(5):1043–1057.e8. doi: 10.1016/j.molcel.2019.06.037 (PMC6739424; doi:10.1016/j.molcel.2019.06.037)
Supplement: Document S2. Article plus Supplemental Information [file mmc3.pdf]

Molecular Cell

Article

Osh Proteins Control Nanoscale Lipid Organization Necessary for PI(4,5)P_2 Synthesis

Graphical Abstract

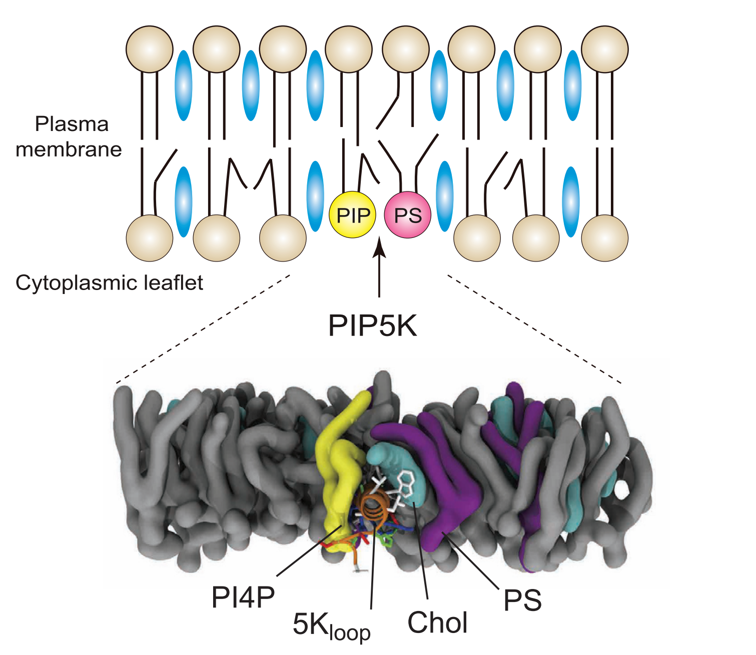

Highlights

- The Osh lipid exchange proteins are required to maintain PI(4,5)P_2 levels in the PM
- Unsaturated PS and sterols synergistically stimulate PIP5K activity
- The specificity loop conserved in PIP5Ks serves as a lipid sensor
- A simulation model of the PIP5K specificity loop embedded in a lipid bilayer

Authors

Taki Nishimura, Michael Gecht, Roberto Covino, ..., Hiroyuki Arai, Nozomu Kono, Christopher J. Stefan

Correspondence

taki.nishimura@crick.ac.uk (T.N.), c.stefan@ucl.ac.uk (C.J.S.)

In Brief

Nishimura et al. show that the Osh lipid exchange proteins are required for PI(4,5) P_2 synthesis, a key regulator of global plasma membrane organization and function. The Osh proteins form a unique membrane lipid environment with unsaturated phosphatidylserine and sterols that specifies PIP 5-kinase activity at the plasma membrane.

Nishimura et al., 2019, Molecular Cell *75*, 1043–1057
September 5, 2019 © 2019 The Author(s). Published by Elsevier Inc.
https://doi.org/10.1016/j.molcel.2019.06.037

Osh Proteins Control Nanoscale Lipid Organization Necessary for $PI(4,5)P_2$ Synthesis

Taki Nishimura,[1,8,*] Michael Gecht,[2] Roberto Covino,[2] Gerhard Hummer,[2,3] Michal A. Surma,[4,9] Christian Klose,[4] Hiroyuki Arai,[5,6] Nozomu Kono,[5,7] and Christopher J. Stefan[1,10,*]
[1]MRC Laboratory for Molecular Cell Biology, University College London, Gower Street, London WC1E 6BT, UK
[2]Department of Theoretical Biophysics, Max Planck Institute of Biophysics, 60438 Frankfurt am Main, Germany
[3]Institute for Biophysics, Goethe University Frankfurt, 60438 Frankfurt am Main, Germany
[4]Lipotype GmbH, Tatzberg 47, 01307 Dresden, Germany
[5]Department of Health Chemistry, Graduate School of Pharmaceutical Sciences, The University of Tokyo, Bunkyo-ku, Tokyo 113-0033, Japan
[6]AMED-CREST, Japan Agency for Medical Research and Development, 1-7-1 Otemachi, Chiyodaku, Tokyo 100-0004, Japan
[7]PRIME, Japan Agency for Medical Research and Development, 1-7-1 Otemachi, Chiyodaku, Tokyo 100-0004, Japan
[8]Present address: Molecular Cell Biology of Autophagy, The Francis Crick Institute, London NW1 1AT, UK
[9]Present address: Łukasiewicz Research Network–PORT Polish Center for Technology Development, Wroclaw, Poland
[10]Lead Contact
*Correspondence: taki.nishimura@crick.ac.uk (T.N.), c.stefan@ucl.ac.uk (C.J.S.)
https://doi.org/10.1016/j.molcel.2019.06.037

SUMMARY

The plasma membrane (PM) is composed of a complex lipid mixture that forms heterogeneous membrane environments. Yet, how small-scale lipid organization controls physiological events at the PM remains largely unknown. Here, we show that ORP-related Osh lipid exchange proteins are critical for the synthesis of phosphatidylinositol (4,5)-bisphosphate [$PI(4,5)P_2$], a key regulator of dynamic events at the PM. In real-time assays, we find that unsaturated phosphatidylserine (PS) and sterols, both Osh protein ligands, synergistically stimulate phosphatidylinositol 4-phosphate 5-kinase (PIP5K) activity. Biophysical FRET analyses suggest an unconventional co-distribution of unsaturated PS and phosphatidylinositol 4-phosphate (PI4P) species in sterol-containing membrane bilayers. Moreover, using *in vivo* imaging approaches and molecular dynamics simulations, we show that Osh protein-mediated unsaturated PI4P and PS membrane lipid organization is sensed by the PIP5K specificity loop. Thus, ORP family members create a nanoscale membrane lipid environment that drives PIP5K activity and $PI(4,5)P_2$ synthesis that ultimately controls global PM organization and dynamics.

INTRODUCTION

Distinctions in membrane lipid composition establish organelle identity in eukaryotic cells (Bigay and Antonny, 2012). For example, the endoplasmic reticulum (ER) and plasma membrane (PM) have notably different lipid compositions (Schneiter et al., 1999). The cytoplasmic leaflet of the ER membrane is defined by low sterol content and is high in unsaturated phospholipids. In contrast, the cytoplasmic leaflet of the PM is enriched in distinct lipids that serve as hallmarks for its identity, including sterols and the anionic phospholipid phosphatidylserine (PS). Among the phospholipids, phosphatidylinositol (4,5)-bisphosphate [$PI(4,5)P_2$] is another key determinant for PM identity and function (Balla, 2013; Di Paolo and De Camilli, 2006). $PI(4,5)P_2$ has vital roles in many events at the PM, including exocytosis, endocytosis, cytoskeletal dynamics, cytokinesis, ion channel regulation, and the generation of second messenger molecules (Balla, 2013; Di Paolo and De Camilli, 2006). Yet, although physiological roles for $PI(4,5)P_2$ at the PM have been intensely studied, less is known about the regulation of $PI(4,5)P_2$ metabolism.

Heterogeneous lipid distribution must also be considered in understanding PM organization (Harayama and Riezman, 2018). Biological membranes are not homogeneous, and lipids are not uniformly distributed within a membrane. Differences in the physical properties of lipids are proposed to induce membrane lipid segregation and the formation of lipid nanodomains (Fujimoto and Parmryd, 2017; Lingwood and Simons, 2010). Accordingly, although lipid composition per se is not fully conserved across organisms, the biophysical principles that govern membrane organization are universally applied (Kaiser et al., 2011; van Meer et al., 2008). As such, it is apparent that lipid heterogeneity contributes to subcompartmental organization and that lipid organizing principles have advantages in vital functions of the PM (Fujimoto and Parmryd, 2017).

Selective lipid transport tends to increase lipid heterogeneity and, thus, distinct membrane environments (Antonny et al., 2018; Bigay and Antonny, 2012). A conserved family of lipid exchange proteins, the oxysterol-binding protein related proteins (ORPs), is thought to transfer lipids, including PS and sterols from the ER to the PM or to late Golgi and secretory compartments in exchange for phosphatidylinositol 4-phosphate (PI4P) (Chung et al., 2015; de Saint-Jean et al., 2011; Mesmin

This is an open access article under the CC BY license (http://creativecommons.org/licenses/by/4.0/).

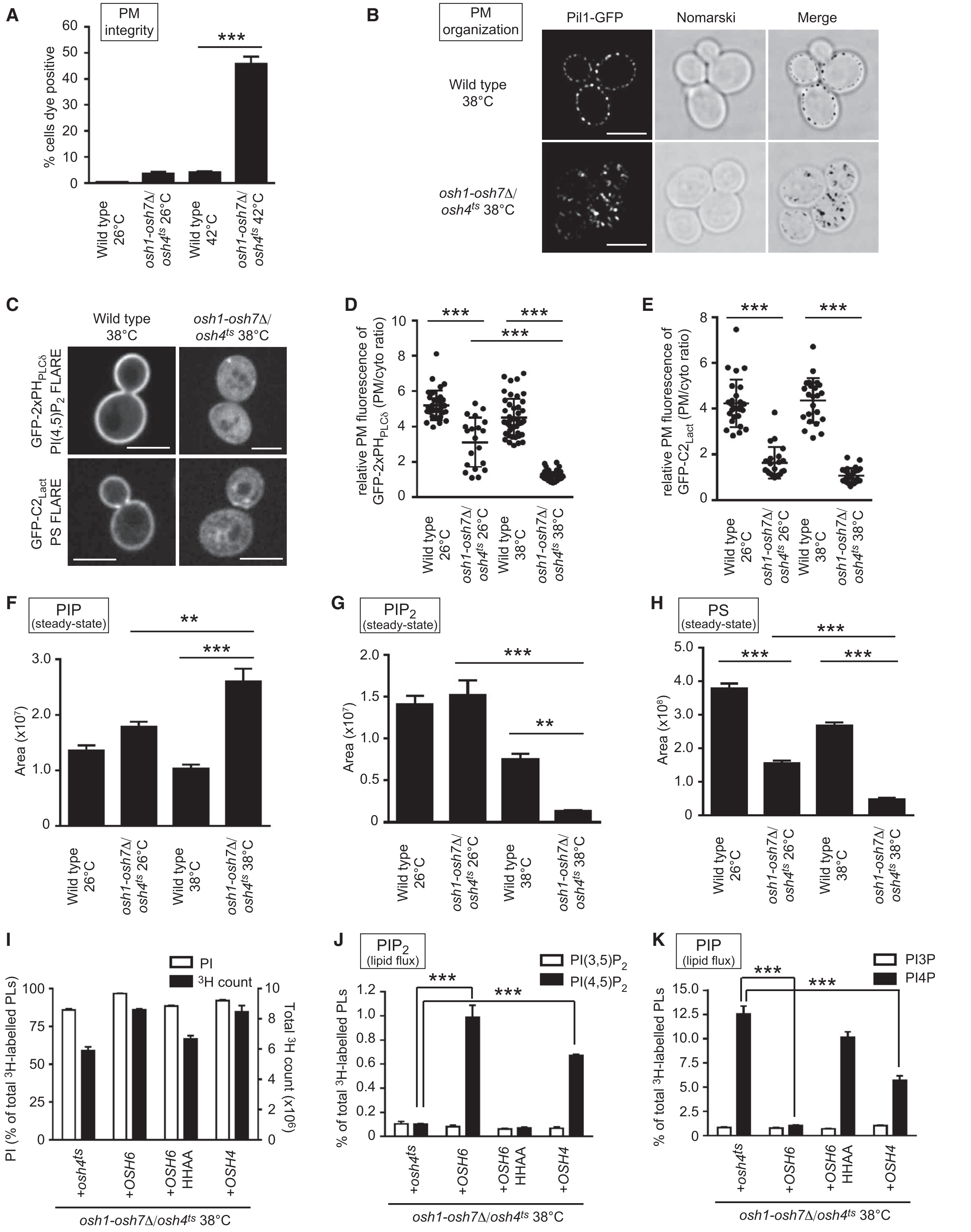

(legend on next page)

et al., 2013; Moser von Filseck et al., 2015a). ORP function is critical for cell growth and survival (Charman et al., 2014). Accordingly, deletion of all four ORP genes in *C. elegans* is embryonic lethal upon cholesterol restriction (Kobuna et al., 2010). Likewise, yeast cells lacking the ORP-related Osh proteins (oxysterol-binding protein homology) are inviable and are impaired in endocytosis (Beh and Rine, 2004), polarized secretion (Alfaro et al., 2011), PI4P metabolism (Stefan et al., 2011) and sterol organization in the PM (Georgiev et al., 2011). However, ORP/Osh protein function is controversial (Menon, 2018) and the essential role for ORP-mediated lipid exchange remains unknown.

We find that the Osh proteins are critical for PI(4,5)P_2 synthesis. Quantitative lipidomics and microscopy show that PI(4,5)P_2 and PS levels are severely reduced upon Osh protein inactivation. PI4P 5-kinase (PIP5K) shows increased activity against PI4P in the presence of unsaturated PS and this effect is markedly enhanced by sterols. Accordingly, FRET analyses imply that unsaturated PS and PI4P co-distribute in the presence of sterols. Finally, *in vivo* imaging and molecular dynamics (MD) simulations suggest that the specificity loop of PIP5K is a lipid sensor that ensures high PIP5K activity at the PM. Thus, Osh protein-mediated lipid exchange results in nanoscale membrane lipid environments necessary for PIP5K activity that controls global PM domain organization and dynamic PM events including exocytosis and endocytosis.

RESULTS

Osh Proteins and ER-PM Contacts Control PI(4,5)P_2 Synthesis

PM identity is defined by its specialized lipid composition. In particular, PI4P and PI(4,5)P_2 are important phosphoinositide lipid species in the PM and both regulate essential processes at the PM (Balla, 2013). Because the ORP/Osh proteins share conserved functions in PI4P binding and metabolism (de Saint-Jean et al., 2011; Im et al., 2005; Moser von Filseck et al., 2015a; Stefan et al., 2011), we reasoned they may control PI4P use (i.e., in lipid exchange reactions and PI(4,5)P_2 synthesis) and, thus, PM organization. To test this, we used *osh1-7Δ/osh4^{ts}* mutant yeast cells that lack the *OSH1–OSH7* genes and carry a temperature-sensitive *osh4-1* allele (Beh and Rine, 2004). We first examined whether Osh proteins maintain PM integrity by using propidium iodide that does not penetrate membranes and enters cells only upon loss of PM integrity. Upon brief PM stress conditions, more than 40% of *osh1-7Δ/osh4^{ts}* cells scored as propidium iodide positive, whereas wild-type cells exhibited negligible staining (Figure 1A). We also examined PM domain organization by monitoring the PI(4,5)P_2-binding protein Pil1, a major component of PM structures termed eisosomes (Karotki et al., 2011). Pil1-GFP was observed at cortical sites in wild-type cells but accumulated in intracellular puncta in *osh1-7Δ/osh4^{ts}* cells (Figure 1B). Thus, both PM integrity and organization are significantly affected in *osh1-7Δ/osh4^{ts}* cells. Based on the observation that similar defects have been observed in cells with impaired PIP5K activity (Karotki et al., 2011; Omnus et al., 2016), we wondered if Osh proteins might regulate PI(4,5)P_2 synthesis.

We investigated whether the Osh proteins control PI(4,5)P_2 localization and levels. PM localization of the PI(4,5)P_2 FLARE (fluorescent lipid-associated reporter) GFP-2xPH$_{PLC\delta}$ was significantly reduced in *osh1-7Δ/osh4^{ts}* cells at the restrictive temperature (Figures 1C and 1D). We also measured phosphoinositide levels by liquid chromatography-electrospray ionization-tandem mass spectrometry (LC-ESI-MS/MS) analysis (Clark et al., 2011). Mono-phosphorylated phosphatidylinositol (PIP) was significantly increased in *osh1-7Δ/osh4^{ts}* cells at the restrictive temperature (38°C; Figure 1F). In contrast, the level of phosphatidylinositol bis-phosphate (PIP_2) was drastically reduced (Figure 1G), consistent with the microscopy results (Figures 1C and 1D). To further define changes in phosphoinositide metabolic flux, we performed ^{3}H-inositol labeling for 1 h and high-performance liquid chromatography (HPLC) analysis. This showed a major reduction in PI(4,5)P_2 synthesis in *osh1-7Δ/osh4^{ts}* cells at the restrictive temperature (Figure S1B), but inositol uptake and phosphatidylinositol (PI) synthesis were not dramatically impaired (Figure S1A). Consistent with intact PI production, PI4P synthesis increased by more than an order of magnitude in the mutant cells (Figure S1C), as previously published (Stefan et al., 2011). In rescue experiments, PI(4,5)P_2 synthesis was restored by expression of wild-type Osh4 from a plasmid (Figure S1E). In contrast, mutant forms of Osh4 impaired in PI4P binding (Osh4HH143,144AA and Osh4Δ29) (de Saint-Jean et al., 2011; Moser von Filseck et al., 2015b) did not rescue PI(4,5)P_2 synthesis (Figure S1E). Likewise, expression of wild-type, but not mutant, Osh4 decreased PI4P in *osh1-7Δ/osh4^{ts}* cells (Figure S1F). Notably, the labeling experiments also indicated that PI4P synthesis exceeds PI(4,5)P_2 synthesis (compare the scales in Figures S1B and S1C). This suggests that PI4P may be consumed during Osh-mediated lipid exchange reactions to promote PIP5K activity and PI(4,5)P_2 production.

Figure 1. Osh Proteins Maintain PI(4,5)P_2 and PS Levels at the PM

(A) PM integrity of wild-type and *osh1-7Δ/osh4^{ts}* cells. Cells incubated at 26°C or 42°C for 15 min were stained with propidium iodide and analyzed by flow cytometry. Data represent the mean ± SEM (n = 3).

(B) Pil1-GFP localization in wild-type and *osh1-7Δ/osh4^{ts}* cells.

(C) PI(4,5)P_2 (GFP-2xPH$_{PLC\delta}$) and PS (GFP-C2$_{Lact}$) FLARE localization in wild-type and *osh1-7Δ/osh4^{ts}* cells.

(B and C) Cells were shifted to 38°C for 2 h. Scale bars, 4 μm.

(D and E) Quantitation of GFP-2xPH$_{PLC\delta}$ (D) and GFP-C2$_{Lact}$ (E) signals in the PM. Relative PM and cytosolic signals were measured as described in STAR Methods. Data represent mean ± SD (n ≥ 20 cells).

(F–H) Lipidomic analysis of PIP (F), PIP_2 (G), and PS (H) in cells cultured at 26°C or 38°C for 2 h. Data represent mean ± SEM (n = 5).

(I–K) Measurements of inositol incorporation and PI synthesis (I), synthesis of the PIP_2 variants PI(3,5)P_2 and PI(4,5)P_2 (J), and synthesis of the PIP variants PI3P and PI4P (K) as monitored by ^{3}H-inositol labeling and HPLC analysis of cells cultured at 38°C for 1 h. Data represent the mean ± SEM (n = 3).

$^{**}p < 0.01$, $^{***}p < 0.001$. See also Figure S1.

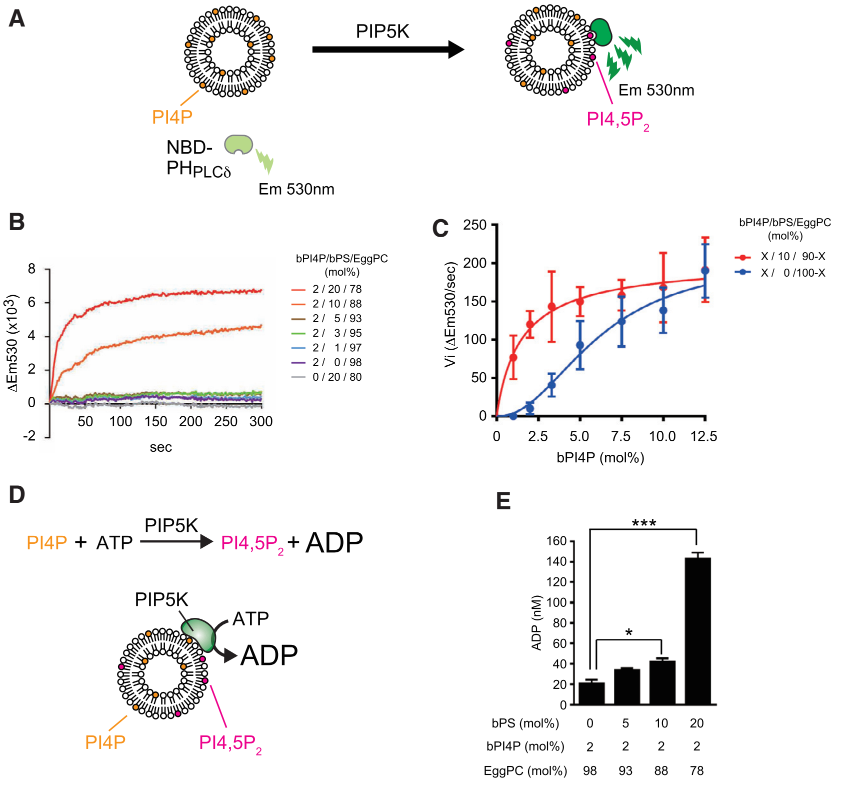

Figure 2. PS Activates PIP5K *In Vitro*

(A) Scheme for the real-time PIP5K assay. NBD-$PH_{PLC\delta}$ fluorescence increases upon binding PI(4,5)P_2 generated by PIP5K on liposomes.

(B) Liposomes containing the indicated amount of brain PI4P (bPI4P), brain PS (bPS), and egg PC were mixed with 60 nM zebrafish PIP5K (zPIP5K) and 400 nM NBD-$PH_{PLC\delta}$. After addition of ATP, NBD fluorescence was recorded by fluorescence spectroscopy. Data represent mean values (n = 3; SEM < 1.12 × 10^3).

(C) zPIP5K activity against various PI4P concentrations in the absence and presence of 10 mol% bPS. Initial velocities (Vi) were determined from PIP5K reaction progress curves (shown in Figures S2E and S2F). Data represent the mean ± SEM (n ≥ 3).

(D) Measurement of PIP5K activity by the ADP-Glo kinase assay. The formation of ADP produced by PIP5K reactions was detected using bioluminescence.

(E) Measurement of PIP5K activity by the ADP-Glo kinase assay (time = 2 min) on liposomes containing the indicated amount of bPI4P, bPS, and egg PC. Data represent mean ± SEM (n = 3).

*p < 0.05, ***p < 0.001. See also Figure S2.

The PM contains high concentrations of sterols, and its cytoplasmic leaflet is enriched in certain lipids, including phosphatidylethanolamine (PE) and PS (Bigay and Antonny, 2012). Osh6 and Osh7 are proposed to serve as PI4P and PS exchange proteins that transfer newly synthesized PS from the ER to the PM (Moser von Filseck et al., 2015a). Accordingly, the PM intensity of the PS FLARE GFP-$C2_{Lact}$ was significantly reduced in *osh1-7Δ/osh4^{ts}* cells (Figures 1C and 1E) even at the permissive temperature of 26°C. Moreover, MS lipid analysis indicated that the amount of PS was significantly reduced in *osh1-7Δ/osh4^{ts}* cells (by >50% and >80% at the permissive and restrictive temperatures, respectively; Figure 1H). The reduction in PS levels may be due to a negative feedback mechanism in the ER membrane reported for both mammalian and yeast PS synthases (Kannan et al., 2017; Sousa et al., 2014). Independent quantitative lipidomics experiments confirmed that PS levels were significantly reduced in *osh1-7Δ/osh4^{ts}* cells (Supplemental Information). Interestingly, expression of wild-type Osh6 from a plasmid restored PI, PI4P, and PI(4,5)P_2 homeostasis (Figures 1I–1K). In contrast, a mutant form of Osh6 impaired in PI4P binding did not rescue PI(4,5)P_2 synthesis (Figure 1J) or PI4P homeostasis (Figure 1K).

We also examined mutant cells (named Δtether cells) lacking six ER-PM "tether" proteins that accumulate PI4P at the PM (Manford et al., 2012). Similar to *osh1-7Δ/osh4^{ts}* cells, PM localization of the PI(4,5)P_2 FLARE mCherry-2x$PH_{PLC\delta}$ was reduced in Δtether cells (Figure S1G). Thus, although PI4P accumulates in *osh1-7Δ/osh4^{ts}* and Δtether cells, it is not an efficient substrate for PIP5K. In addition, the PM fluorescence intensity of the PS FLARE GFP-$C2_{Lact}$ and PS levels were reduced in the Δtether cells compared to wild type (Figure S1H and Supplemental Information). Based on these initial findings, we next considered whether PIP5K selectively uses PI4P in a sterol- and PS-enriched environment formed by Osh proteins and ER-PM contacts.

PS Enhances PIP5K Activity *In Vitro*

To study the lipid requirements for PIP5K activity, we developed a real-time PIP5K assay by using a PI(4,5)P_2 sensor, NBD-$PH_{PLC\delta}$, to monitor PI(4,5)P_2 levels on liposomes by fluorescence spectroscopy. As nitrobenzoxadiazole (NBD) displays enhanced fluorescence at 530 nm in a hydrophobic environment, increases in NBD fluorescence intensity (ΔEm530) indicate recruitment of NBD-$PH_{PLC\delta}$ to liposomes (Figure 2A). We confirmed linear increases in NBD-$PH_{PLC\delta}$ fluorescence intensity in response to PI(4,5)P_2 across a dynamic range (0.125–1 mol%; Figure S2A). Recombinant zebrafish PIP5K1A (zPIP5K amino acid [aa] 49–431) (Hu et al., 2015) was used for PIP5K assays. Expression of zPIP5K restored the growth defect of yeast PIP5K mutant *mss4^{ts}* cells, suggesting zPIP5K recognizes yeast and metazoan lipid species (Figure S2B). In control PIP5K assays, liposomes containing 5 mol% brain-derived PI4P (bPI4P) were mixed with zPIP5K, and NBD-$PH_{PLC\delta}$ signal intensity was measured by fluorescence spectroscopy prior to (time = 0 s) and after addition of ATP. The NBD fluorescence intensity rapidly increased in an ATP, Mg^{2+}, and zPIP5K dose-dependent manner (Figure S2C). Liposome size did not significantly affect zPIP5K activity in the presence of 5 mol% bPI4P (Figure S2D). We observed zPIP5K activity only at concentrations of bPI4P (3.3 mol% and higher; Figure S2E) that are well above concentrations reported in the PM (<0.2 mol% of total PM lipids) (Yoshida et al., 2016). This suggests additional factors promote robust PIP5K activity *in vivo*.

We, therefore, examined whether PS stimulates PIP5K activity in the real-time PIP5K assay. Interestingly, when liposomes also contained 10 mol% or 20 mol% of brain-derived phosphatidylserine (bPS), PIP5K activity was clearly detected against 2 mol % bPI4P (Figures 2B and S2F). For comparison, PS makes up >30% of phospholipids in the PM of yeast cells (Zinser et al., 1991). Consistent with previous work (Shulga et al., 2012), phosphatidic acid (PA) also stimulated PIP5K activity, but other phospholipids found in the PM, including PI and PE, did not (Figure S2G). Kinetic analyses confirmed that bPS activates PIP5K (Figure 2C). The apparent K_m value of zPIP5K for PI4P was effectively reduced from 6.2 mol% to 1.5 mol% in the presence of bPS without obvious changes in V_{max} (Figure 2C). The sensitivity of the PI(4,5)P_2 sensor also increased in the presence of PS by nearly 2-fold (Figure S2A), but this does not fully account for the 4-fold increase in zPIP5K activity (Figures 2C, S2E, and S2F). To confirm the positive effect of bPS on zPIP5K activity, we used an ADP-Glo kinase assay that detects ADP produced by the PIP5K kinase reaction (Figure 2D). ADP formation by PIP5K was significantly increased in the presence of bPS (Figure 2E). Collectively, these results suggest that PS efficiently enhances PIP5K activity *in vitro*.

Sterol and Unsaturated PS Synergistically Enhance PIP5K Activity

We also investigated the effect of sterol lipids shown to bind Osh proteins *in vitro* (de Saint-Jean et al., 2011; Im et al., 2005; Manik et al., 2017; Moser von Filseck et al., 2015b). Cholesterol enhanced zPIP5K activity in the real-time PIP5K assay (Figure 3A) without influencing the sensitivity of the PI(4,5)P_2 sensor NBD-$PH_{PLC\delta}$ (Figure S3A). The apparent K_m value of PIP5K for PI4P was reduced from 6.2 mol% to 3.0 mol% in the presence of cholesterol (Figure 3A). We then analyzed the simultaneous effect of bPS and cholesterol on zPIP5K activity. When the amount of bPI4P in liposomes was reduced to 1 mol%, a positive effect on zPIP5K activity by either bPS or cholesterol alone was weak or not clearly observed (Figure 3B). In contrast, zPIP5K activity increased dramatically in the presence of both bPS and cholesterol (Figure 3B). These results suggest that cholesterol and bPS synergistically stimulate PIP5K activity.

We next investigated whether the fatty acid composition of PS and PI4P affects PIP5K stimulation, as bPS and bPI4P are composed of multiple species including polyunsaturated forms. Compared to saturated di-palmitoyl-phosphatidylserine (16:0/16:0 DPPS), cholesterol was effective in stimulating PIP5K in the presence of mono-unsaturated 1-palmitoyl-2-oleyl-phosphatidylserine (16:0/18:1 POPS) and di-unsaturated di-oleoyl-phosphatidylserine (18:1/18:1 DOPS) (Figures 3C and 3G). zPIP5K showed the highest activity against bPI4P in the presence of DOPS and cholesterol (Figure 3C). Cholesterol did not enhance PI(4,5)P_2 probe sensitivity in the presence of DOPS, and the PI(4,5)P_2 probe displayed only slight differences in sensitivity under the various conditions (Figure S3B). We also examined the effect of lipid composition on PIP5K membrane affinity and found, in contrast to the PI(4,5)P_2 probe, that PIP5K binding to 1 mol% bPI4P liposomes increased upon addition of both DOPS and cholesterol (Figure 3D). In line with the real-time assays, zPIP5K showed the highest activity against bPI4P in the presence of both DOPS and cholesterol in the ADP-Glo kinase assay (Figure S3C). Time course experiments using the ADP-Glo assay confirmed that PIP5K activity occurred rapidly in the presence of DOPS and cholesterol (reaching completion by 120 s; Figure S3G). In contrast, changes in free phosphate levels were negligible and did not increase over the course of the experiment (Figure S3H), confirming that the ADP-Glo assays measured ADP produced by PIP5K-dependent PI(4,5)P_2 synthesis rather than non-specific ATP hydrolysis. Altogether, these results indicate that PIP5K demonstrates high activity in the presence of DOPS and cholesterol.

Yeast cells do not synthesize polyunsaturated phospholipids or cholesterol (see the Supplemental Information and Figure 4). However, similar trends were observed with the yeast lipid species 1-palmitoyl-2-oleyl PI4P (16:0/18:1 PO-PI4P) and ergosterol. Ergosterol enhanced PIP5K enzymatic activity and membrane binding against 1 mol% PO-PI4P in the presence of unsaturated PS (DOPS again was most effective; Figures 3E–3G). Likewise, ergosterol stimulated zPIP5K activity against 1 mol% PO-PI4P liposomes containing unsaturated PS in the ADP-Glo kinase assay (DOPS and ergosterol resulted in the highest activity; Figure S3G). Thus, PIP5K displays high activity in a membrane environment containing sterol and unsaturated PS species. Moreover, the unsaturated PI4P species bPI4P (18:0/20:4) and PO-PI4P (16:0/18:1) were better substrates than saturated di-palmitoyl PI4P (16:0/16:0 DP-PI4P) (Figure S3E), consistent with a previous study (Shulga et al., 2012). Finally, PIP5K showed robust activity against liposomes containing physiological levels of PO-PI4P (0.2 mol%) and additional lipids resembling the composition of the cytoplasmic leaflet of the yeast PM, dependent upon unsaturated PS and ergosterol (Figure S3F). Collectively, our results indicate that PIP5K prefers a membrane lipid environment containing unsaturated PI4P, unsaturated PS, and sterol.

Fatty Acid Unsaturation of PS and PI4P Are Maintained in *osh1-7Δ/osh4^{ts}* Cells

Considering that PIP5K prefers unsaturated PI4P and is activated by unsaturated PS, we examined the fatty acid composition of PS, PI, and phosphoinositide species in wild-type and *osh1-7Δ/osh4^{ts}* cells. The major species of PS, PI, PIP, and PIP_2 in control cells were unsaturated (mono-unsaturated X:1 and di-unsaturated X:2 where X refers to total acyl chain length), whereas saturated species (X:0) were rare (Figures 4A, 4B, S4A, and S4B). The most abundant PS and PIP species in wild-type control cells were mono-unsaturated POPS and PO-PI4P (34:1; Figures 4A and 4B). In *osh1-7Δ/osh4^{ts}* cells, POPS levels decreased and PO-PI4P levels increased (Figures 4A and 4B), consistent with the proposed role for Osh6/7 as PI4P and POPS exchange proteins (Moser von Filseck et al., 2015a). Intriguingly, mono-unsaturated PIP_2 was slightly enriched in comparison to mono-unsaturated PIP and PI in wild-type cells (Figure S4C), suggesting yeast PIP5K may prefer mono-unsaturated PI4P. Although overall PIP levels were increased in *osh1-7Δ/osh4^{ts}* cells (Figures 1F and 4B), major changes in acyl chain profiles were not observed. A small portion of PIP shifted from 36:X acyl species (where X refers to any level of unsaturation) to shorter PIP 32:X species in *osh1-7Δ/osh4^{ts}* cells (Figure S4D).

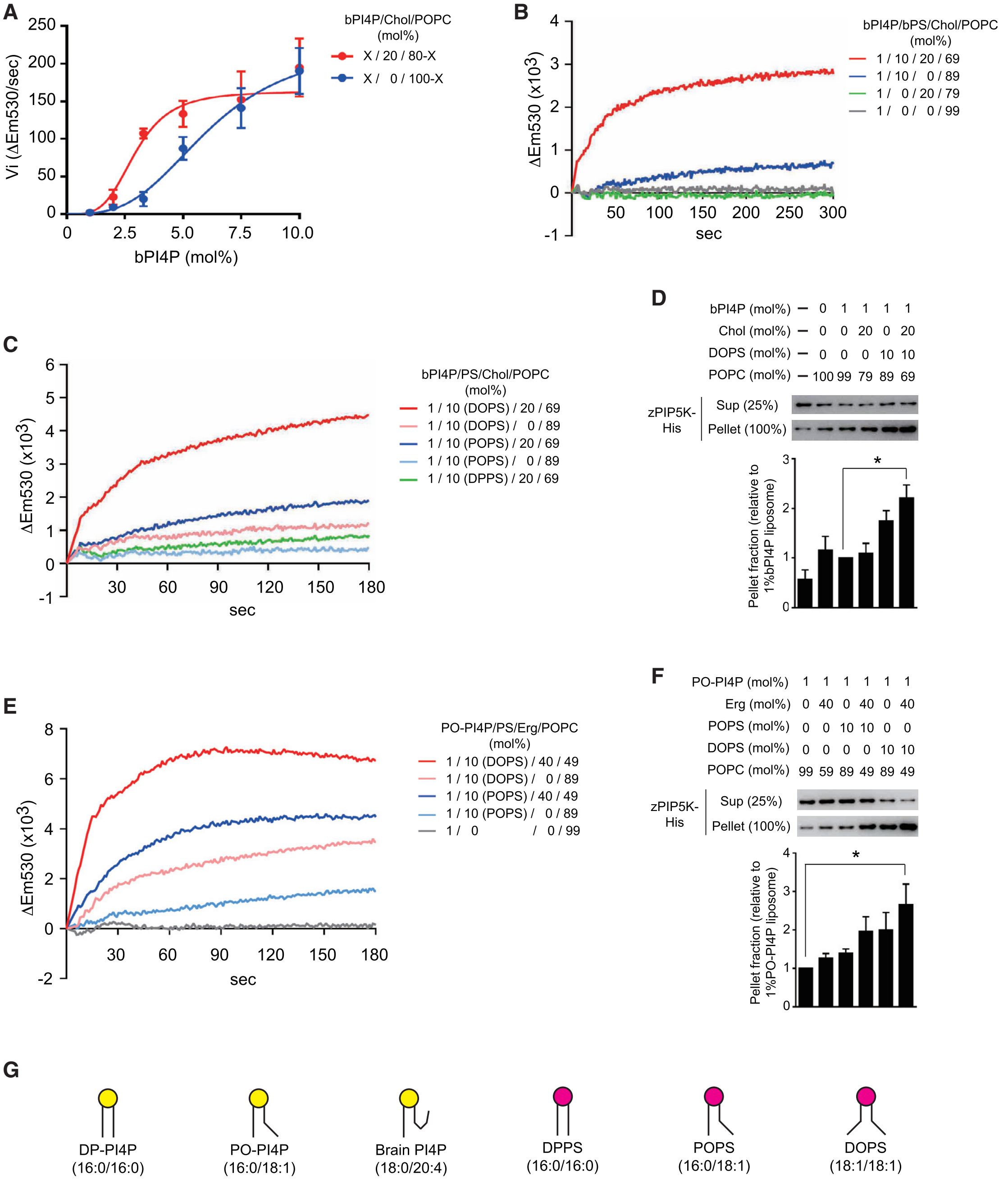

Figure 3. PIP5K Stimulation by Unsaturated PS and Sterols *In Vitro*

(A) PI4P concentration dependence of zPIP5K activity in the absence and presence of 20 mol% cholesterol (Chol). Initial velocities (Vi) were determined from PIP5K reaction progress curves from real-time assays. Data represent mean ± SEM (n = 3).

(B) The activity of zPIP5K toward 1 mol% bPI4P liposomes either lacking or containing bPS and/or cholesterol as indicated. Data represent mean values (n = 3; SEM < 0.25×10^3).

(C) The activity of zPIP5K toward 1 mol% bPI4P liposomes containing the indicated PS species either lacking or containing cholesterol as indicated. Data represent mean values (n $\geq$ 6; SEM < 0.48×10^3).

(D) PIP5K sedimentation assays using liposomes containing bPI4P, DOPS, and cholesterol as indicated. Data represent mean ± SEM (n = 3).

(E) The activity of zPIP5K toward 1 mol% PO-PI4P liposomes containing the indicated PS species either lacking or containing ergosterol (Erg) as indicated. Data represent mean values (n = 3; SEM < 1.20×10^3).

(F) PIP5K sedimentation assays using 1 mol% PO-PI4P liposomes either lacking or containing the indicated PS and/or Erg. Data represent mean ± SEM (n = 3).

(G) Phospholipids used in the PIP5K assays.

*$p < 0.05$. See also Figure S3.

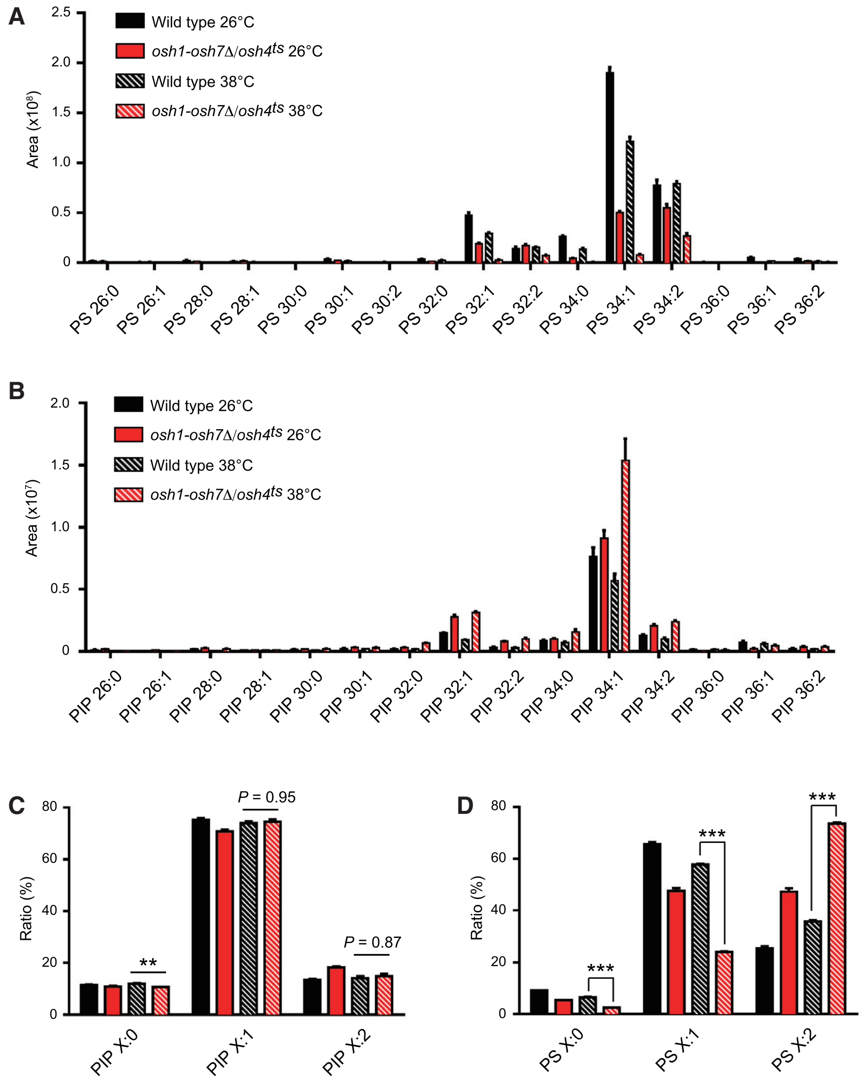

Figure 4. Fatty Acid Unsaturation of PIP and PS Are Maintained in *osh1-7Δ/osh4^ts* Cells

Lipidomic analysis of PS and PIP in wild-type and *osh1-7Δ/osh4^ts* cells cultured at 26°C or 38°C for 2 h. Data represent mean ± SEM (n = 5).

(A and B) Fatty acid compositions of PS (A) and PIP (B).

(C and D) Fatty acid unsaturation degree of PIP (C) and PS (D).

**p < 0.01, ***p < 0.001. See also Figure S4.

However, the overall degree of PIP unsaturation was not significantly affected in *osh1-7Δ/osh4^ts* cells (Figure 4C). As such, the distribution of PIP species did not shift toward saturated species that are poor substrates for PIP5K (Figures 4B and 4C). Likewise, the degree of PS saturation did not increase in *osh1-7Δ/osh4^ts* cells, but instead shifted toward unsaturated species (Figures 4A and 4D). In sum, these results suggest that Osh function has a minor impact on acyl chain length and saturation of PI4P. Rather, a major function for the Osh proteins may be to create a PM lipid environment containing unsaturated PI4P and PS that is suitable for PIP5K activity.

Unsaturated PS and PI4P May Co-distribute in the Presence of Sterols

Sterols are thought to stably interact with saturated lipids but not with unsaturated lipids (Simons and Gerl, 2010). To evaluate whether sterol lipids affect the distribution of unsaturated PS and PI4P, we designed an *in vitro* FRET (Förster resonance energy transfer) assay (Figures 5A and 5B). FRET is well suited to evaluate proximity in the nanometer range and can detect the co-assembly of lipids within a membrane bilayer (Simons and Gerl, 2010). The CFP-tagged PS sensor (CFP-C2$_{Lact}$) and Venus-tagged PI4P sensor (Venus-P4C) were mixed with liposomes of defined compositions, and the FRET signal was measured (Figure 5B). When liposomes contained either bPI4P or bPS, FRET was not observed (Figures 5B and 5C). However, FRET was observed in the presence of both bPI4P and bPS, and the FRET signal was further increased by the addition of cholesterol (Figures 5B and 5C), suggesting that bPS and bPI4P are in close apposition to each other in the presence of cholesterol. Similarly, the co-distribution of unsaturated DOPS and bPI4P was enhanced in the presence of cholesterol (Figure 5D). Likewise, ergosterol increased the co-distribution of PO-PI4P with unsaturated POPS and DOPS (Figure 5E). Binding of the PI4P probe to liposomes was not significantly enhanced by the presence of cholesterol and DOPS (Figures S5A and 5B). Likewise, membrane binding of the PS probe was not significantly enhanced by the presence of cholesterol and PI4P (Figures S5C), consistent with a previous study (Hirama et al., 2017). Thus, the observed increases in FRET efficiencies between the PI4P and PS probes (Figures 5B and 5E) are not likely due to increased membrane recruitment of the lipid probes. Sterols may cause and/or enhance the co-distribution of unsaturated PS and PI4P *in vitro*. However, these experiments do not fully exclude sterol-induced effects on lipid probe binding affinities or membrane fluidity on FRET efficiencies.

PM Targeting of the PIP5K Specificity Loop Depends on PI4P, PS, and Sterol

The substrate specificity and membrane targeting of PIP5Ks are determined by the specificity loop within their catalytic domains (Figure 6A) (Fairn et al., 2009; Hu et al., 2015; Kunz et al., 2000). The specificity loop of PIP5K (5K$_{loop}$) folds into an amphipathic helix (AH) upon membrane binding (Figures 6B and 6C) (Liu et al., 2016). We checked if an amphipathic property of the 5K$_{loop}$ is required for PIP5K function. The purified yPIP5K/Mss4 kinase

CellPress

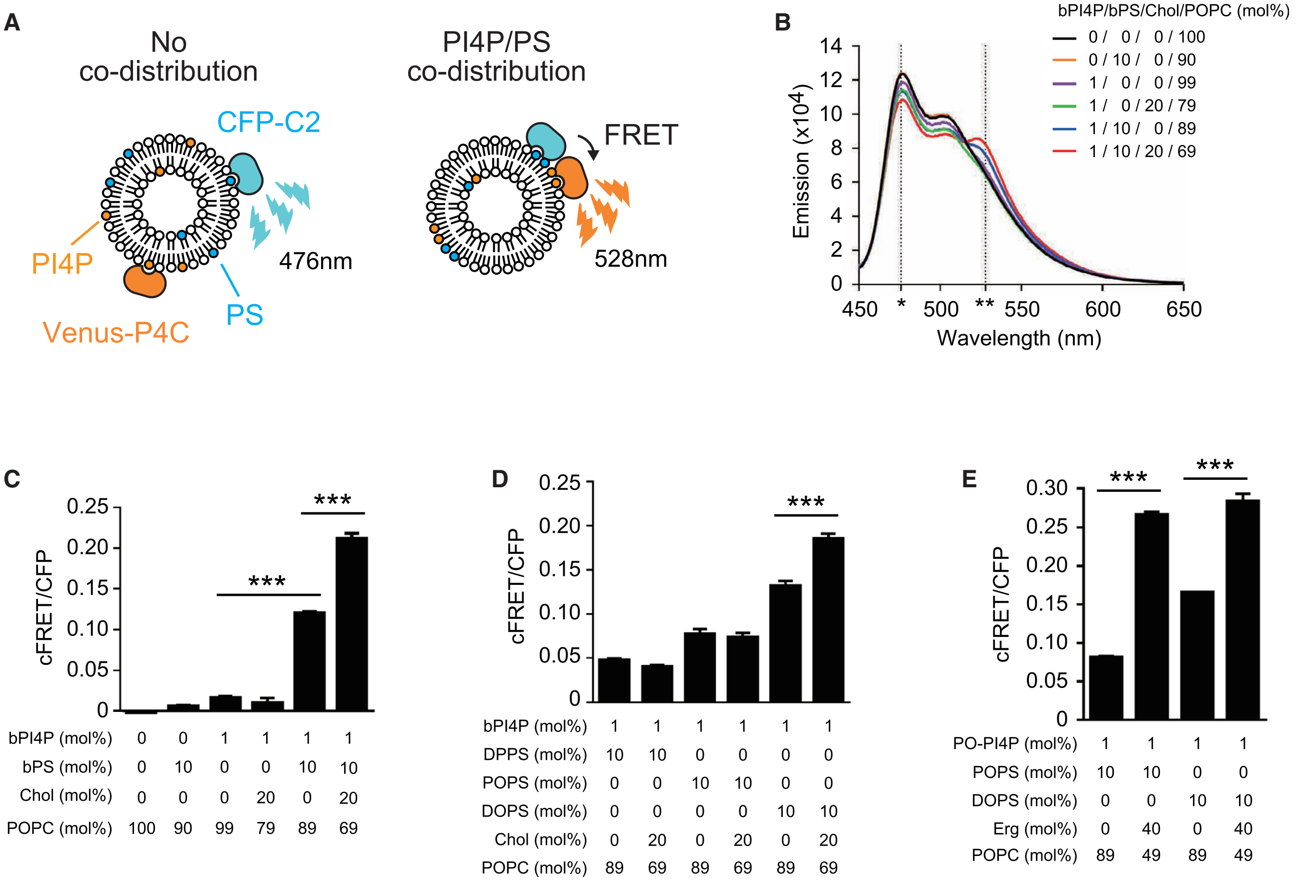

Figure 5. Co-distribution of Unsaturated PS and PI4P in the Presence of Sterols *In Vitro*

(A) The *in vitro* FRET assay to detect PI4P and PS co-distribution on liposomes. Corrected FRET (cFRET) was calculated as described in STAR Methods.

(B) Emission spectrum of Venus-P4C (PI4P probe) and CFP-$C2_{Lact}$ (PS probe) *in vitro*, as described in STAR Methods. The x and y axis indicate wavelength and intensity of emission fluorescence, respectively. Note that FRET signal was increased in the presence of both brain phosphatidylserine (bPS) and cholesterol (Chol). Asterisks indicate positions of emission maximum of CFP at 476 nm (*) and FRET at 528 nm (**).

(C) FRET toward liposomes either lacking or containing 1 mol% bPI4P, bPS, and/or cholesterol (Chol) as indicated.

(D) FRET toward 1 mol% bPI4P liposomes containing the indicated PS species either lacking or containing Chol.

(E) FRET toward 1 mol% PO-PI4P liposomes containing the indicated PS species either lacking or containing ergosterol (Erg). Data represent mean ± SEM (n = 3). ***$p < 0.001$. See also Figure S5.

domain showed PIP5K activity in the presence of both bPS and cholesterol, whereas the L722K/L729K mutant protein bearing mutations in the hydrophobic face showed low PIP5K activity (Figure 6D). These results suggest that an amphipathic property of the $5K_{loop}$ is critical for PIP5K activity. Moreover, basic residues on the opposite face of the $5K_{loop}$ were required, as a negatively charged mutant K720D/K721D did not display PIP5K activity (Figure 6D). We also investigated requirements for the amphipathic property of the $5K_{loop}$ *in vivo*. The L722K/L729K and K720D/K721D mutant forms neither localized to the PM (Figure S6A) nor restored the growth defect of *mss4*^{ts} cells (Figures S2B). On the other hand, a kinase dead mutant (K571A) lacking PIP5K activity (Figure 6D) localized to the PM (Figure S6A). Thus, an amphipathic property of the $5K_{loop}$ is required not only for PIP5K activity *in vitro* but also for PIP5K localization and function *in vivo*.

To further examine $5K_{loop}$ PM targeting, the specificity loop of zPIP5K (zPIP5Kγ aa 380–403) was fused to a GFP-tagged coiled-coil domain ($5K_{loop}$-GCC-GFP; Figure 6E) to enhance its membrane affinity (Horchani et al., 2014). In wild-type yeast cells, $5K_{loop}$-GCC-GFP localized to the PM (Figure 6F). In contrast, the $5K_{loop}$-GCC-GFP mislocalized from the PM to intracellular compartments in *osh1-7Δ/osh4*^{ts} cells (Figures 6F and 6G). Consistent with this, PM targeting of the yeast PIP5K Mss4 was significantly reduced in *osh1-7Δ/osh4*^{ts} mutant cells at the restrictive temperature (Figure S6B). We, therefore, examined lipid requirements for $5K_{loop}$ PM targeting. We first confirmed if the $5K_{loop}$ depended on PI4P by using *pik1*^{ts}, *stt4*^{ts}, and *pik1*^{ts}/*stt4*^{ts} double mutant cells impaired in the major PI 4-kinase activities in yeast (Audhya et al., 2000). The PM localization of $5K_{loop}$-GCC-GFP was partially reduced in *pik1*^{ts} and *stt4*^{ts} single mutants and nearly disappeared in *pik1*^{ts}/*stt4*^{ts} double mutant cells (Figure S6C). In contrast, PM localization of $5K_{loop}$-GCC-GFP was not reduced in *mss4*^{ts} mutant cells impaired in PI(4,5)P_2 synthesis (Figure S6D), indicating that $5K_{loop}$-GCC-GFP mislocalization is not caused by reductions in PI(4,5)P_2. Importantly,

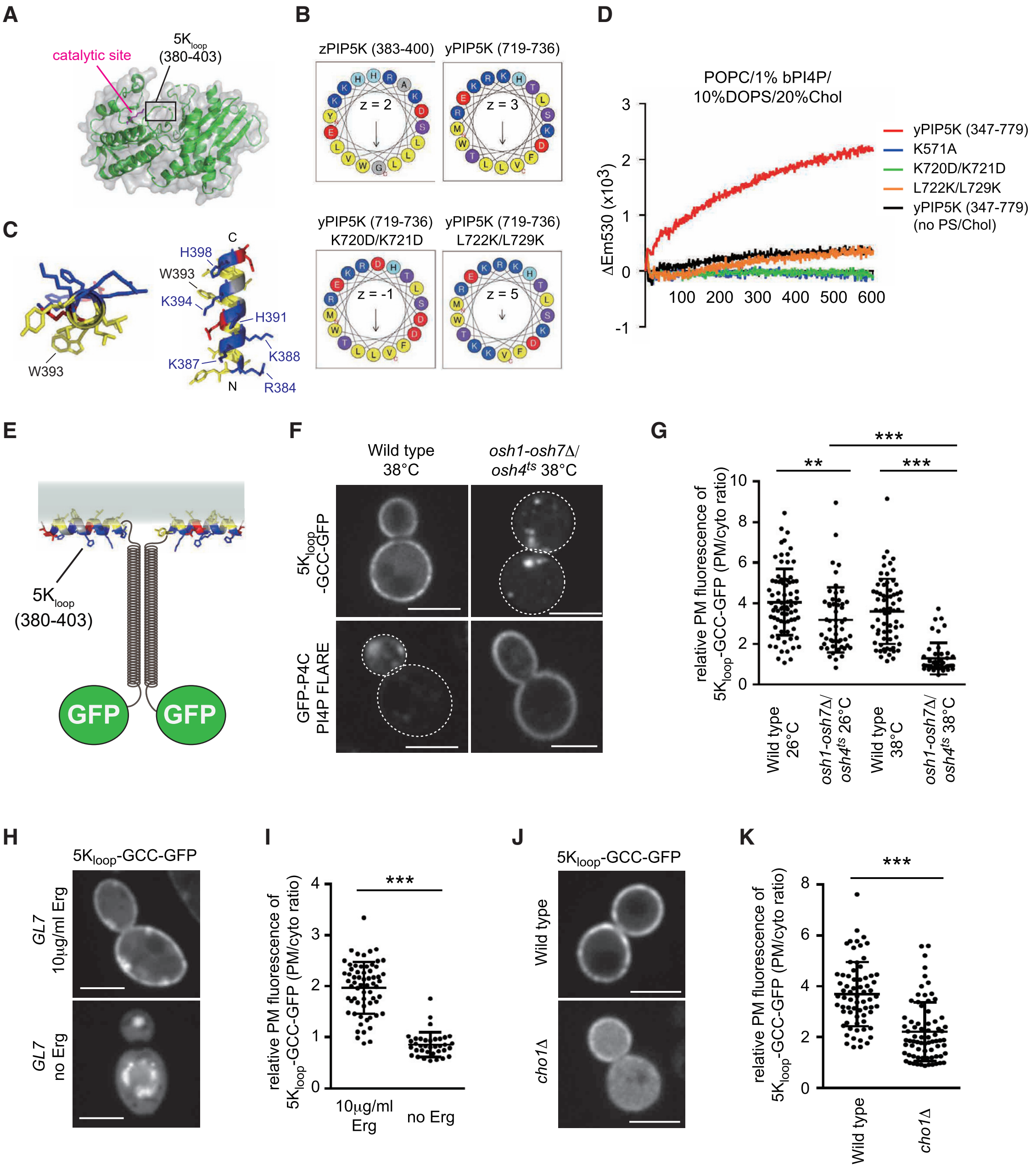

Figure 6. The PIP5K Specificity Loop Requires Osh Proteins, PS, and Sterols for PM Targeting

(A) Crystal structure of zebrafish PIP5K (zPIP5K) (Hu et al., 2015). The specificity loop region ($5K_{loop}$: residues 380–403) was disordered in the crystal structure. The catalytic site (residue K236 in zPIP5K corresponding to K571 in yeast PIP5K) is shown in magenta.

(B) The amphipathic properties of the specificity loop of zPIP5K and yeast PIP5K (yPIP5K). Helical wheel representations were drawn using HeliQuest. Hydrophobic residues are shown in yellow, arginine and lysine in dark blue, histidine in light blue, serine in purple, and glutamate and aspartate in red. Substitutions disrupting charge and the amphipathic property of the specificity loop of yPIP5K are shown in the bottom row (K720D/K721D and L722K/L729K, respectively). Z indicates net charge and arrows in helical wheels correspond to the hydrophobic moment.

(C) The amphipathic character of the specificity loop of zPIP5K ($5K_{loop}$) using PEP-FOLD 3 and PyMOL.

(D) Real-time PIP5K assays using 200 nM wild-type or mutant forms of the yeast PIP5K domain (yPIP5K). K571A is a kinase-dead form of yPIP5K. Data represent mean values ($n \geq 3$; SEM < 0.61×10^3).

(E) Design of the $5K_{loop}$-GCC-GFP FLARE.

(F) Localization of $5K_{loop}$-GCC-GFP and GFP-P4C (PI4P FLARE) in wild-type and *osh1-7Δ/osh4^{ts}* cells cultured at 38°C for 2 h.

(G) $5K_{loop}$-GCC-GFP PM signal in wild-type and *osh1-7Δ/osh4^{ts}* cells. Data represent mean ± SD ($n \geq 46$ cells).

(H) Localization of $5K_{loop}$-GCC-GFP in the sterol auxotroph *GL7* strain cultured with or without 10 μg/ml ergosterol (Erg).

(legend continued on next page)

the PI4P FLARE GFP-P4C accumulated at the PM in *osh1-7*Δ/*osh4[ts]* cells (Figure 6F). Thus, although PI4P is required for $5K_{loop}$ PM targeting, PI4P alone is not sufficient. We then analyzed whether sterol is required for $5K_{loop}$-GCC-GFP PM localization by using the auxotrophic sterol strain *GL7* that also has been shown to be impaired in PI(4,5)P_2 synthesis (Dahl and Dahl, 1985). Localization of $5K_{loop}$-GCC-GFP to the PM in *GL7* mutant cells depended on exogenous ergosterol (Figures 6H and 6I). In addition to sterol dependence, the PM targeting of $5K_{loop}$-GCC-GFP was significantly reduced in *cho1*Δ mutant cells deficient in PS synthesis (Figures 6J and 6K). Thus, PM localization of the $5K_{loop}$ depended on PI4P, PS, and sterol *in vivo*. Our *in vitro* assays indicated that unsaturated PI4P and PS promote PIP5K membrane binding and activity (Figure 3). To investigate a requirement for lipid unsaturation in $5K_{loop}$ PM targeting and PI(4,5)P_2 synthesis *in vivo*, we analyzed *ole1*Δ mutant cells lacking the major fatty acid desaturase activity in yeast (Ogasawara et al., 2017). Upon removal of exogenous unsaturated fatty acid from the medium, both GFP-2xPH$_{PLC\delta}$ and $5K_{loop}$-GCC-GFP displayed reduced PM localization in *ole1*Δ mutant cells (Figures S6E and S6F). Altogether, our results suggest that the $5K_{loop}$ recognizes unsaturated PI4P and PS membrane environments stabilized by sterol *in vivo*.

PIP5K Specificity Loop Shows Specific Interactions in Bilayers

To study the molecular interactions of the $5K_{loop}$ with bilayers, we ran extensive MD simulations in atomistic resolution. We modeled the zPIP5K peptide (zPIP5Kγ aa 380–403) as an α-helix and inserted it onto a bilayer composed of 69 mol% POPC, 10 mol% DOPS, 1 mol% bPI4P (18:0/20:4), and 20 mol% cholesterol (the composition activating zPIP5K). After solvation and equilibration, we simulated three replicas of the system for a cumulative time of 24 μs in the atomistic CHARMM36m representation (Huang et al., 2017). During all simulations the $5K_{loop}$ remained stably inserted in the bilayer, displaying only a partial unfolding of few residues at the C terminus (Figures 7A–7C). The $5K_{loop}$ equilibrates relatively deep in the membrane, with the residues of its hydrophobic interface inserted in the hydrophobic core of the bilayer (Figures 7A–7C).

To gain a systematic view on how the different lipid species interact with the $5K_{loop}$, we calculated their localization probabilities over the cumulated simulation time. POPC does not form specific contacts with the $5K_{loop}$, and despite its abundance in the model bilayer, its localization is marginally affected by the presence of the helix (compare the two leaflets in Figure 7D). By contrast, DOPS molecules form contacts with the $5K_{loop}$ by electrostatic interactions (Figure 7E), and cholesterol transiently associates with the $5K_{loop}$ (Figure 7F). Given the low molar concentration, only a few bPI4P molecules are present in the bilayer we simulated. However, in all our simulations, a bPI4P quickly associates to the $5K_{loop}$ forming a contact that remains stable for the rest of the simulations, mostly due to its net charge. This is reflected in the probability maps (Figure 7G) showing that bPI4P strongly interacts with two sites along the $5K_{loop}$.

Visual inspection of the trajectories reveals a rich set of interactions between the $5K_{loop}$ and the different lipid species. Cholesterol molecules reversibly associate to the $5K_{loop}$, filling the packing defect created by the insertion of the helix and interacting with aromatic residues (e.g., W393; Figure 7B). Interestingly, the cholesterol hydrocarbon chain also wedges between the acyl chains of the polyunsaturated PI4P stably associated with the $5K_{loop}$ (Figures 7B, 7H, and 7J). Electrostatic interactions drive the formation of contacts between the charged headgroups of DOPS and bPI4P, and the positively charged residues located on the hydrophilic face of the $5K_{loop}$. DOPS and bPI4P can occupy distinct pockets along opposing sides of the $5K_{loop}$ at the same time (Figures 7B, 7E, and 7G). It is important to note, however, that PI4P and DOPS are almost never found on the same side of the $5K_{loop}$. This is due to the highly charged PI4P headgroup occupying all electrostatic interactions on one side of the $5K_{loop}$. This observation is in line with the localization plots of the densities of each lipid on opposing sides (Figures 7E and G). PI4P is situated very close to the $5K_{loop}$, whereas DOPS is relatively far away. Nevertheless, DOPS interacts by its headgroup with residues K387 and K394 and also pushes its hydrophobic tails under the $5K_{loop}$, where cholesterol is wedged in (Figure 7B). bPI4P forms intimate interactions with the $5K_{loop}$, almost wrapping around it, maximizing the contact both with its headgroup and its polyunsaturated acyl chain (Figures 7C, 7H, and 7J). Indeed, bPI4P can interact with the $5K_{loop}$ by its glycerol backbone, the 1-phosphate moiety between the backbone and inositol, and the hydroxyl-groups and 4-phosphate moiety on the inositol ring (Figures 7H–7J and S7A–S7F). The latter enables the headgroup of bPI4P to bridge over several lipids and interact with the $5K_{loop}$ even if the lipid tails are relatively far away (Figures S7D–S7F). These observations suggest that association of bPI4P and the $5K_{loop}$ should be described in terms of a dynamic network of electrostatic interactions that can be mediated by other lipid molecules.

DISCUSSION

Our findings show that Osh-mediated membrane lipid dynamics and ER-PM contacts promote PIP5K activity at the PM (Figure S7G). Remarkably, our data indicate that only a small pool of PI4P synthesized in the cell is used as a substrate for PI(4,5)P_2 synthesis. PI4P appears to be largely consumed during Osh-mediated lipid exchange reactions (Figures 1, S1, and 4). In this manner, newly synthesized PS and sterol lipids may be transported from the ER to the PM by Osh proteins in exchange for PI4P (Figure S7G). The Osh proteins may then transfer PI4P to the ER (Antonny et al., 2018) or directly present PI4P to the Sac1 PI4P phosphatase at ER-PM contacts (Stefan et al., 2011). Both modes of Sac1 activity (in *cis* and in *trans*) would promote continued rounds of lipid transfer by the Osh proteins, as both

(I) $5K_{loop}$-GCC-GFP PM signal in *GL7* cells. Data represent mean ± SD ($n \geq 36$ cells).
(J) Localization of $5K_{loop}$-GCC-GFP in wild-type and *cho1*Δ mutant cells supplemented with 1 mM ethanolamine.
(K) $5K_{loop}$-GCC-GFP PM signal in wild-type and *cho1*Δ cells. Data represent mean ± SD ($n \geq 69$ cells). Scale bars, 4 μm.
$^{**}p < 0.01$, $^{***}p < 0.001$. See also Figure S6.

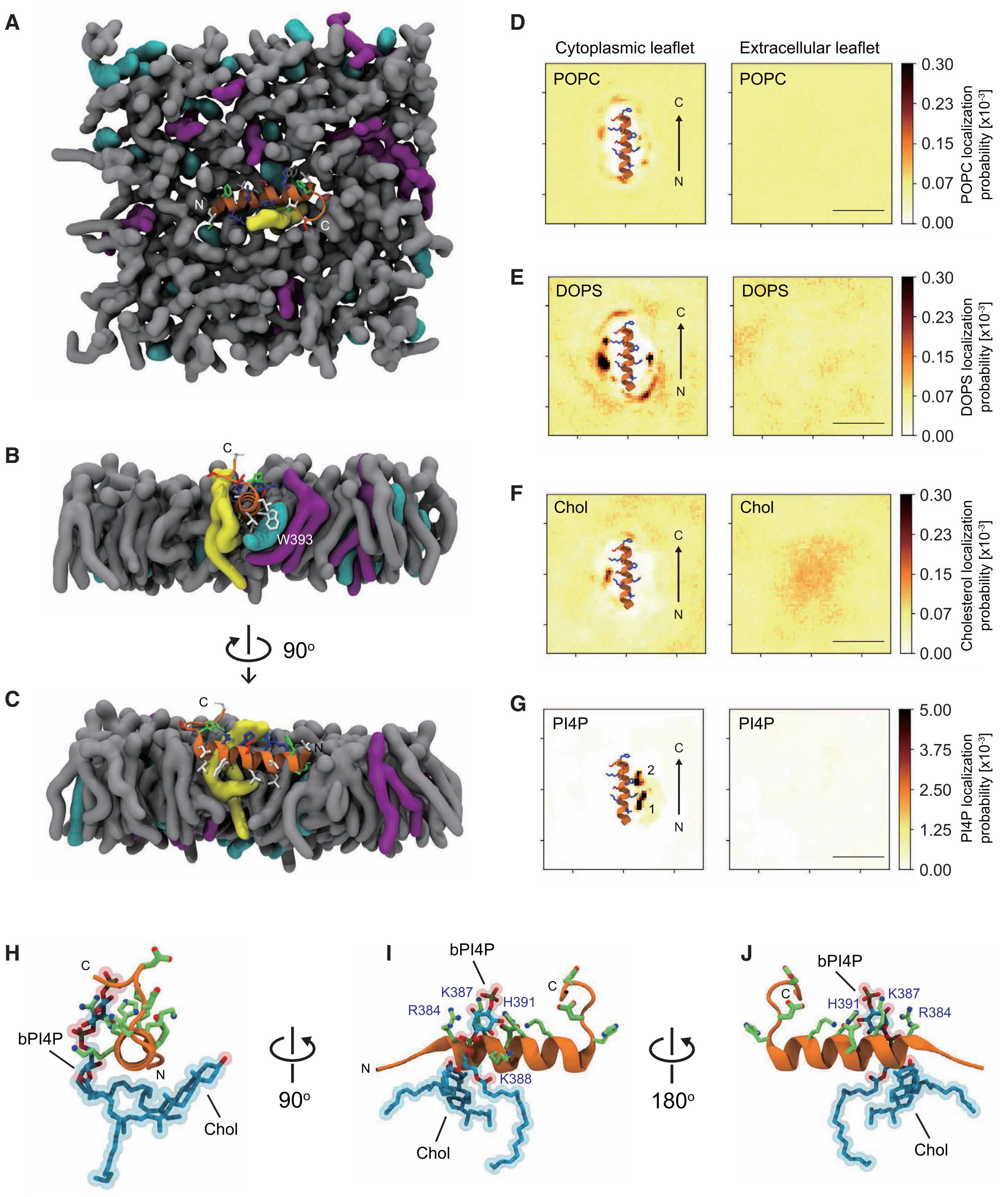

Figure 7. Molecular View of the PIP5K Specificity Loop Embedded in a Lipid Bilayer

(A–C) The specificity loop of PIP5K ($5K_{loop}$) embedded into a model membrane bilayer in atomistic molecular dynamics simulations. The $5K_{loop}$ is shown as ribbon (orange) with hydrophobic, polar, acidic, and basic amino acids represented as sticks in white, green, red, and blue, respectively. The surfaces of lipids and sterols are shown for POPC (gray), DOPS (magenta), cholesterol (cyan), and bPI4P (yellow). The figure shows an area of the membrane leaflet of approximately 4 × 4 nm (A). Cross sectional views of the $5K_{loop}$ embedded in the model membrane bilayer (B and C). Water and ions outside the membrane are omitted for clarity.

(D–G) Lipid localization. Time-averaged positions of the phosphate moieties of the phospholipids POPC (D), DOPS (E), and PI4P (G) or oxygen atoms in cholesterol (Chol, F) from all-atom molecular dynamics simulations of a model membrane bilayer. Colors indicate the localization probability of different lipids over the course of the whole 10-μs trajectory. The membrane leaflet (cytoplasmic) with the $5K_{loop}$ embedded is shown in the left panels; the opposing leaflet (extracellular) is shown on the right. Scale bars, 2.5 nm.

(H–J) Atomistic views of the bPI4P molecule interacting with pocket #1 of the $5K_{loop}$ with a cholesterol molecule on the other side as shown in Figure 7B (H); 90° orientation (I); 180° orientation (J). The two phosphate moieties of bPI4P interact with four basic residues of the amphipathic helix (R384, K387, K388, and H391).

(legend continued on next page)

mechanisms would release PI4P bound to Osh proteins. Thus, PI4P serves two vital functions in the cell. It is the substrate for PIP5K and it is used as a "currency" for ORP/Osh-mediated lipid exchange that stimulates PIP5K activity. Importantly, our data indicate that PI4P alone is not sufficient for PIP5K activity. However, once PS and sterol lipids reach sufficient levels, PI4P metabolism switches from PI4P exchange and hydrolysis by the Osh and Sac1 system to PI(4,5)P_2 synthesis by PIP5K (Figure S7G). This has two significant implications. First, PI4P extraction by Osh proteins will decrease upon PS and sterol delivery because of competition between PI4P and PS or sterol for the Osh proteins (Moser von Filseck et al., 2015a). Second, PI4P, PS, and sterol synergistically promote PIP5K targeting and activity. Thus, non-vesicular lipid dynamics taking place at ER-PM contacts may promote the formation of PIP5K assemblies that ultimately drive vesicular trafficking events in adjacent ER-free PM zones.

ORP and Osh proteins may also exchange PI4P for PS and sterol at late Golgi and secretory compartments (Antonny et al., 2018) (Figure S7G). This may promote secretory vesicle maturation and allow rapid conversion of PI4P to PI(4,5)P_2 immediately prior to or following vesicle fusion with the PM. Alternatively, PI4P extracted from late Golgi compartments and other organelles may be directly transferred to the PM by Osh proteins. Both of these mechanisms could explain the involvement of Golgi PI4P pools in $5K_{loop}$ PM targeting and PI(4,5)P_2 synthesis (Figure S6) (Audhya et al., 2000) and why cells depleted of ER-PM contacts are viable (Manford et al., 2012). Osh proteins also control sterol distribution within the PM (Georgiev et al., 2011). Accordingly, ORP/Osh proteins might move lipids within the PM resulting in PIP5K activation.

Our study confirms key roles for the ORP/Osh proteins and ER-PM contacts in PM identity. Both are required for enrichment of PS in the cytoplasmic leaflet of the PM (Figures 1 and S1), consistent with previous reports (Chung et al., 2015; Moser von Filseck et al., 2015a). Our findings unexpectedly suggest that the ORP/Osh proteins also create heterogeneous PM environments and, thus, transient unconventional lipid interactions at the nanoscale level. Numerous studies have described physical interactions between saturated lipids and sterols. Yet, less attention has been paid to the effect of sterols on unsaturated lipids, in spite of the fact that PS and PI4P are key components of the inner leaflet of the PM (Balla, 2013; Yeung et al., 2008) and most of them have unsaturated fatty acids (Figure 4). Our analyses suggest that unsaturated PI4P and PS co-distribution might be modulated by sterols (Figure 5). Unsaturated fatty acids are incorporated into the *sn-2* position in most phosphoinositides (PIPs) (Harayama and Riezman, 2018), but unsaturated lipids are not efficiently incorporated into sterol-containing liquid-ordered domain. One possible explanation is that unsaturated lipids are condensed by their repulsive interaction with sterols (Krause and Regen, 2014). Alternatively, our MD simulations indicate that cholesterol interacts with the acyl chains of polyunsaturated PI4P stably associated with the PIP5K specificity loop and fills the packing defect created by insertion of the peptide helix (Figure 7). Additional interactions may also provide important contributions. For example, PS retains cholesterol in the inner leaflet of the PM and cholesterol, in turn, limits electrostatic repulsion between anionic PS molecules (Hirama et al., 2017; Maekawa and Fairn, 2015). This may explain why inactivation of Osh4, shown to transfer sterol lipids *in vitro*, results in decreased PS levels at the PM (Figures 1 and 4). The ionization state of PIPs, dependent on neighboring lipids and ionic strength, may also govern PIP segregation patterns (Wen et al., 2018). Thus multiple factors likely account for the complex behavior of unsaturated phospholipids and sterols in membranes.

Our *in vivo* imaging, *in vitro* assays, and simulation analyses suggest that the conserved PIP5K specificity loop forms an AH that prefers a membrane environment containing unsaturated anionic lipids and sterols. Given that another anionic lipid, PA, also stimulates PIP5K (Shulga et al., 2012), PA might interact with the PIP5K AH similarly to PS. Interestingly, the hydrophobic face of the PIP5K AH contains bulky aromatic residues, similar to other AH sensors that preferentially insert into membrane regions with lipid packing-order defects formed by acyl chain unsaturation. This design may allow PIP5K to recognize unsaturated PI4P species that cluster in intermittent disordered regions of the PM. Given that yeast PO-PI4P was an effective substrate for PIP5K (Figure S3), yeast PI4P might induce lipid-packing defects as much as mammalian PI4P. It is remarkable that the PIP5K AH targets to the PM rather than the highly disordered ER. Accordingly, PM targeting of the PIP5K AH is specified by basic residues within the AH that bind PI4P and PS (and PA) in the cytoplasmic leaflet of the PM and is further stabilized by interactions with sterols (Figure 7). Thus, the PIP5K AH is endowed with distinctive chemical and physical properties, compared to previously described AH membrane sensors (Bigay and Antonny, 2012; Covino et al., 2018; Hofbauer et al., 2018), allowing it to detect a unique PM environment containing PI4P, PS, and sterol. Although PIP5K AH targeting to the PM depended on both PS and PI4P, our analyses indicate increased binding affinity for PI4P over PS. This suggests an order of events for PIP5K targeting and activity. The ORP/Osh proteins may form a PM environment enriched in unsaturated PS stabilized by sterols that initially recruits PIP5K (Figure S7G).

The human ORP5 and ORP8 proteins transfer unsaturated PS species (Chung et al., 2015), suggesting they may be involved in PI(4,5)P_2 regulation. In turn, PI(4,5)P_2 controls ORP5 and ORP8 PM localization (Ghai et al., 2017; Sohn et al., 2018), indicating cross-regulation of PS and PI(4,5)P_2 homeostasis. Intriguingly, ORP5 and ORP8 knock down results in increased PI(4,5)P_2 levels (Ghai et al., 2017; Sohn et al., 2018). But PI4P is increased and PS levels are maintained to some extent upon ORP5 and ORP8 depletion, implying that other ORP isoforms or other lipid

The cholesterol hydrocarbon chain wedges between the acyl chains of the polyunsaturated PI4P. The amphipathic helix is visualized as a ribbon (orange) and acidic and basic residues as sticks in green. Positively charged nitrogen atoms and negatively charged oxygen atoms are shown as spheres, colored blue and red, respectively.
See also Figure S7.

transfer proteins may be induced under these conditions. As such, future studies are likely to reveal key roles for additional lipid transfer proteins in PM lipid heterogeneity and organization.

STAR★METHODS

Detailed methods are provided in the online version of this paper and include the following:

- KEY RESOURCES TABLE
- LEAD CONTACT AND MATERIALS AVAILABILITY
- EXPERIMENTAL MODEL AND SUBJECT DETAILS
  - Yeast
- METHOD DETAILS
  - Plasmids
  - Fluorescence Microscopy
  - Analysis of ^{3}H-labeled Inositol Phosphates by HPLC
  - LC-MS/MS analysis of methylated PIPs and PS
  - Quantitative shotgun lipid MS data acquisition, analysis, and post-processing
  - Protein purification
  - Preparation of NBD-PH$_{PLC\delta}$ Proteins
  - Liposome preparations
  - Real-time PIP 5-kinase assay
  - ADP-Glo kinase assay
  - Malachite green phosphate assay
  - Liposome sedimentation assay
  - *in vitro* FRET assays
  - Quantitative membrane binding assay using Dansyl-PE liposomes
  - Plasma membrane integrity assays
  - Atomistic simulations
  - Analysis of lipid localization
  - Trajectory analysis
- QUANTIFICATION AND STATISTICAL ANALYSIS
- DATA AND CODE AVAILABILITY

SUPPLEMENTAL INFORMATION

Supplemental Information can be found online at https://doi.org/10.1016/j.molcel.2019.06.037.

ACKNOWLEDGMENTS

We thank Scott Emr, Susan Henry, Yuxin Mao, Greg Fairn, Bruno Antonny, Guillaume Drin, Takeshi Noda, Ya Ha, and W. David Nes for reagents. We thank Mark Marsh, Graham Warren, Shamshad Cockcroft, Tim Levine, Toyoshi Fujimoto, Will Prinz, Robert Ernst, and members of the Stefan lab for helpful discussions. C.J.S. is supported by MRC funding to the MRC LMCB University Unit at UCL, award code MC_UU_00012/6. T.N. was supported by JSPS Postdoctoral Fellowships for Research Abroad and the Osamu Hayaishi Scholarship for Study Abroad. M.G., R.C., and G.H. acknowledge the support of the Max Planck Society.

AUTHOR CONTRIBUTIONS

T.N. and C.J.S. designed the study and performed experiments; M.A.S., C.K., H.A., and N.K. performed lipidomic analyses; M.G., R.C., and G.H. designed and performed simulation analyses; T.N., M.G., R.C., M.A.S., N.K., and C.J.S. analyzed the data; T.N., M.G., R.C., N.K., and C.J.S. wrote the manuscript. All authors discussed the results and commented on the manuscript.

DECLARATION OF INTERESTS

C.K. has paid employment at Lipotype GmbH. This does not alter the authors' adherence to all policies on sharing data and materials. The other authors declare no competing financial interests.

Received: January 8, 2019
Revised: May 13, 2019
Accepted: June 25, 2019
Published: August 8, 2019

REFERENCES

Alfaro, G., Johansen, J., Dighe, S.A., Duamel, G., Kozminski, K.G., and Beh, C.T. (2011). The sterol-binding protein Kes1/Osh4p is a regulator of polarized exocytosis. Traffic *12*, 1521–1536.

Antonny, B., Bigay, J., and Mesmin, B. (2018). The Oxysterol-Binding Protein Cycle: Burning Off PI(4)P to Transport Cholesterol. Annu. Rev. Biochem. *87*, 809–837.

Audhya, A., Foti, M., and Emr, S.D. (2000). Distinct roles for the yeast phosphatidylinositol 4-kinases, Stt4p and Pik1p, in secretion, cell growth, and organelle membrane dynamics. Mol. Biol. Cell *11*, 2673–2689.

Balla, T. (2013). Phosphoinositides: tiny lipids with giant impact on cell regulation. Physiol. Rev. *93*, 1019–1137.

Beh, C.T., and Rine, J. (2004). A role for yeast oxysterol-binding protein homologs in endocytosis and in the maintenance of intracellular sterol-lipid distribution. J. Cell Sci. *117*, 2983–2996.

Berendsen, H.J.C., Postma, J.P.M., van Gunsteren, W.F., DiNola, A., and Haak, J.R. (1984). Molecular-Dynamics with Coupling to an External Bath. J. Chem. Phys. *81*, 3684–3690.

Bigay, J., and Antonny, B. (2012). Curvature, lipid packing, and electrostatics of membrane organelles: defining cellular territories in determining specificity. Dev. Cell *23*, 886–895.

Bussi, G., Donadio, D., and Parrinello, M. (2007). Canonical sampling through velocity rescaling. J. Chem. Phys. *126*, 014101.

Charman, M., Colbourne, T.R., Pietrangelo, A., Kreplak, L., and Ridgway, N.D. (2014). Oxysterol-binding protein (OSBP)-related protein 4 (ORP4) is essential for cell proliferation and survival. J. Biol. Chem. *289*, 15705–15717.

Chung, J., Torta, F., Masai, K., Lucast, L., Czapla, H., Tanner, L.B., Narayanaswamy, P., Wenk, M.R., Nakatsu, F., and De Camilli, P. (2015). Intracellular transport. PI4P/phosphatidylserine countertransport at ORP5- and ORP8-mediated ER-plasma membrane contacts. Science *349*, 428–432.

Clark, J., Anderson, K.E., Juvin, V., Smith, T.S., Karpe, F., Wakelam, M.J., Stephens, L.R., and Hawkins, P.T. (2011). Quantification of PtdInsP3 molecular species in cells and tissues by mass spectrometry. Nat. Methods *8*, 267–272.

Covino, R., Hummer, G., and Ernst, R. (2018). Integrated Functions of Membrane Property Sensors and a Hidden Side of the Unfolded Protein Response. Mol. Cell *71*, 458–467.

Dahl, J.S., and Dahl, C.E. (1985). Stimulation of cell proliferation and polyphosphoinositide metabolism in Saccharomyces cerevisiae GL7 by ergosterol. Biochem. Biophys. Res. Commun. *133*, 844–850.

de Saint-Jean, M., Delfosse, V., Douguet, D., Chicanne, G., Payrastre, B., Bourguet, W., Antonny, B., and Drin, G. (2011). Osh4p exchanges sterols for phosphatidylinositol 4-phosphate between lipid bilayers. J. Cell Biol. *195*, 965–978.

Di Paolo, G., and De Camilli, P. (2006). Phosphoinositides in cell regulation and membrane dynamics. Nature *443*, 651–657.

Ejsing, C.S., Sampaio, J.L., Surendranath, V., Duchoslav, E., Ekroos, K., Klemm, R.W., Simons, K., and Shevchenko, A. (2009). Global analysis of the yeast lipidome by quantitative shotgun mass spectrometry. Proc. Natl. Acad. Sci. USA *106*, 2136–2141.

Fairn, G.D., Ogata, K., Botelho, R.J., Stahl, P.D., Anderson, R.A., De Camilli, P., Meyer, T., Wodak, S., and Grinstein, S. (2009). An electrostatic switch

displaces phosphatidylinositol phosphate kinases from the membrane during phagocytosis. J. Cell Biol. *187*, 701–714.

Fujimoto, T., and Parmryd, I. (2017). Interleaflet Coupling, Pinning, and Leaflet Asymmetry-Major Players in Plasma Membrane Nanodomain Formation. Front. Cell Dev. Biol. *4*, 155.

Georgiev, A.G., Sullivan, D.P., Kersting, M.C., Dittman, J.S., Beh, C.T., and Menon, A.K. (2011). Osh proteins regulate membrane sterol organization but are not required for sterol movement between the ER and PM. Traffic *12*, 1341–1355.

Ghai, R., Du, X., Wang, H., Dong, J., Ferguson, C., Brown, A.J., Parton, R.G., Wu, J.W., and Yang, H. (2017). ORP5 and ORP8 bind phosphatidylinositol-4, 5-biphosphate (PtdIns(4,5)P $_2$) and regulate its level at the plasma membrane. Nat. Commun. *8*, 757.

Harayama, T., and Riezman, H. (2018). Understanding the diversity of membrane lipid composition. Nat. Rev. Mol. Cell Biol. *19*, 281–296.

Herzog, R., Schwudke, D., Schuhmann, K., Sampaio, J.L., Bornstein, S.R., Schroeder, M., and Shevchenko, A. (2011). A novel informatics concept for high-throughput shotgun lipidomics based on the molecular fragmentation query language. Genome Biol. *12*, R8.

Herzog, R., Schuhmann, K., Schwudke, D., Sampaio, J.L., Bornstein, S.R., Schroeder, M., and Shevchenko, A. (2012). LipidXplorer: a software for consensual cross-platform lipidomics. PLoS One *7*, e29851.

Hirama, T., Lu, S.M., Kay, J.G., Maekawa, M., Kozlov, M.M., Grinstein, S., and Fairn, G.D. (2017). Membrane curvature induced by proximity of anionic phospholipids can initiate endocytosis. Nat. Commun. *8*, 1393.

Hofbauer, H.F., Gecht, M., Fischer, S.C., Seybert, A., Frangakis, A.S., Stelzer, E.H.K., Covino, R., Hummer, G., and Ernst, R. (2018). The molecular recognition of phosphatidic acid by an amphipathic helix in Opi1. J. Cell Biol. *217*, 3109–3126.

Horchani, H., de Saint-Jean, M., Barelli, H., and Antonny, B. (2014). Interaction of the Spo20 membrane-sensor motif with phosphatidic acid and other anionic lipids, and influence of the membrane environment. PLoS One *9*, e113484.

Hu, J., Yuan, Q., Kang, X., Qin, Y., Li, L., Ha, Y., and Wu, D. (2015). Resolution of structure of PIP5K1A reveals molecular mechanism for its regulation by dimerization and dishevelled. Nat. Commun. *6*, 8205.

Huang, J., Rauscher, S., Nawrocki, G., Ran, T., Feig, M., de Groot, B.L., Grubmüller, H., and MacKerell, A.D., Jr. (2017). CHARMM36m: an improved force field for folded and intrinsically disordered proteins. Nat. Methods *14*, 71–73.

Im, Y.J., Raychaudhuri, S., Prinz, W.A., and Hurley, J.H. (2005). Structural mechanism for sterol sensing and transport by OSBP-related proteins. Nature *437*, 154–158.

Kaiser, H.J., Surma, M.A., Mayer, F., Levental, I., Grzybek, M., Klemm, R.W., Da Cruz, S., Meisinger, C., Müller, V., Simons, K., and Lingwood, D. (2011). Molecular convergence of bacterial and eukaryotic surface order. J. Biol. Chem. *286*, 40631–40637.

Kannan, M., Lahiri, S., Liu, L.K., Choudhary, V., and Prinz, W.A. (2017). Phosphatidylserine synthesis at membrane contact sites promotes its transport out of the ER. J. Lipid Res. *58*, 553–562.

Karotki, L., Huiskonen, J.T., Stefan, C.J., Ziółkowska, N.E., Roth, R., Surma, M.A., Krogan, N.J., Emr, S.D., Heuser, J., Grünewald, K., and Walther, T.C. (2011). Eisosome proteins assemble into a membrane scaffold. J. Cell Biol. *195*, 889–902.

Klose, C., Surma, M.A., Gerl, M.J., Meyenhofer, F., Shevchenko, A., and Simons, K. (2012). Flexibility of a eukaryotic lipidome–insights from yeast lipidomics. PLoS One *7*, e35063.

Kobuna, H., Inoue, T., Shibata, M., Gengyo-Ando, K., Yamamoto, A., Mitani, S., and Arai, H. (2010). Multivesicular body formation requires OSBP-related proteins and cholesterol. PLoS Genet. *6*, e1001055.

Krause, M.R., and Regen, S.L. (2014). The structural role of cholesterol in cell membranes: from condensed bilayers to lipid rafts. Acc. Chem. Res. *47*, 3512–3521.

Kunz, J., Wilson, M.P., Kisseleva, M., Hurley, J.H., Majerus, P.W., and Anderson, R.A. (2000). The activation loop of phosphatidylinositol phosphate kinases determines signaling specificity. Mol. Cell *5*, 1–11.

Lingwood, D., and Simons, K. (2010). Lipid rafts as a membrane-organizing principle. Science *327*, 46–50.

Liu, A., Sui, D., Wu, D., and Hu, J. (2016). The activation loop of PIP5K functions as a membrane sensor essential for lipid substrate processing. Sci. Adv. *2*, e1600925.

Maekawa, M., and Fairn, G.D. (2015). Complementary probes reveal that phosphatidylserine is required for the proper transbilayer distribution of cholesterol. J. Cell Sci. *128*, 1422–1433.

Manford, A.G., Stefan, C.J., Yuan, H.L., Macgurn, J.A., and Emr, S.D. (2012). ER-to-plasma membrane tethering proteins regulate cell signaling and ER morphology. Dev. Cell *23*, 1129–1140.

Manik, M.K., Yang, H., Tong, J., and Im, Y.J. (2017). Structure of Yeast OSBP-Related Protein Osh1 Reveals Key Determinants for Lipid Transport and Protein Targeting at the Nucleus-Vacuole Junction. Structure *25*, 617–629.e613.

Menon, A.K. (2018). Sterol gradients in cells. Curr. Opin. Cell Biol. *53*, 37–43.

Mesmin, B., Bigay, J., Moser von Filseck, J., Lacas-Gervais, S., Drin, G., and Antonny, B. (2013). A four-step cycle driven by PI(4)P hydrolysis directs sterol/PI(4)P exchange by the ER-Golgi tether OSBP. Cell *155*, 830–843.

Moser von Filseck, J., Čopič, A., Delfosse, V., Vanni, S., Jackson, C.L., Bourguet, W., and Drin, G. (2015a). Intracellular transport. Phosphatidylserine transport by ORP/Osh proteins is driven by phosphatidylinositol 4-phosphate. Science *349*, 432–436.

Moser von Filseck, J., Vanni, S., Mesmin, B., Antonny, B., and Drin, G. (2015b). A phosphatidylinositol-4-phosphate powered exchange mechanism to create a lipid gradient between membranes. Nat. Commun. *6*, 6671.

Ogasawara, Y., Kira, S., Mukai, Y., Noda, T., and Yamamoto, A. (2017). Ole1, fatty acid desaturase, is required for Atg9 delivery and isolation membrane expansion during autophagy in Saccharomyces cerevisiae. Biol. Open *6*, 35–40.

Omnus, D.J., Manford, A.G., Bader, J.M., Emr, S.D., and Stefan, C.J. (2016). Phosphoinositide kinase signaling controls ER-PM cross-talk. Mol. Biol. Cell *27*, 1170–1180.

Parrinello, M., and Rahman, A. (1980). Crystal-Structure and Pair Potentials: a Molecular-Dynamics Study. Phys. Rev. Lett. *45*, 1196–1199.

Schneiter, R., Brügger, B., Sandhoff, R., Zellnig, G., Leber, A., Lampl, M., Athenstaedt, K., Hrastnik, C., Eder, S., Daum, G., et al. (1999). Electrospray ionization tandem mass spectrometry (ESI-MS/MS) analysis of the lipid molecular species composition of yeast subcellular membranes reveals acyl chain-based sorting/remodeling of distinct molecular species en route to the plasma membrane. J. Cell Biol. *146*, 741–754.

Shulga, Y.V., Anderson, R.A., Topham, M.K., and Epand, R.M. (2012). Phosphatidylinositol-4-phosphate 5-kinase isoforms exhibit acyl chain selectivity for both substrate and lipid activator. J. Biol. Chem. *287*, 35953–35963.

Simons, K., and Gerl, M.J. (2010). Revitalizing membrane rafts: new tools and insights. Nat. Rev. Mol. Cell Biol. *11*, 688–699.

Sohn, M., Korzeniowski, M., Zewe, J.P., Wills, R.C., Hammond, G.R.V., Humpolickova, J., Vrzal, L., Chalupska, D., Veverka, V., Fairn, G.D., et al. (2018). PI(4,5)P_2 controls plasma membrane PI4P and PS levels via ORP5/8 recruitment to ER-PM contact sites. J. Cell Biol. *217*, 1797–1813.

Sousa, S.B., Jenkins, D., Chanudet, E., Tasseva, G., Ishida, M., Anderson, G., Docker, J., Ryten, M., Sa, J., Saraiva, J.M., et al. (2014). Gain-of-function mutations in the phosphatidylserine synthase 1 (PTDSS1) gene cause Lenz-Majewski syndrome. Nat. Genet. *46*, 70–76.

Stefan, C.J., Manford, A.G., Baird, D., Yamada-Hanff, J., Mao, Y., and Emr, S.D. (2011). Osh proteins regulate phosphoinositide metabolism at ER-plasma membrane contact sites. Cell *144*, 389–401.

Surma, M.A., Herzog, R., Vasilj, A., Klose, C., Christinat, N., Morin-Rivron, D., Simons, K., Masoodi, M., and Sampaio, J.L. (2015). An automated shotgun lipidomics platform for high throughput, comprehensive, and quantitative analysis of blood plasma intact lipids. Eur. J. Lipid Sci. Technol. *117*, 1540–1549.

van Meer, G., Voelker, D.R., and Feigenson, G.W. (2008). Membrane lipids: where they are and how they behave. Nat. Rev. Mol. Cell Biol. *9*, 112–124.

Wen, Y., Vogt, V.M., and Feigenson, G.W. (2018). Multivalent Cation-Bridged PI(4,5)P_2 Clusters Form at Very Low Concentrations. Biophys. J. *114*, 2630–2639.

Yeung, T., Gilbert, G.E., Shi, J., Silvius, J., Kapus, A., and Grinstein, S. (2008). Membrane phosphatidylserine regulates surface charge and protein localization. Science *319*, 210–213.

Yoshida, A., Shigekuni, M., Tanabe, K., and Fujita, A. (2016). Nanoscale analysis reveals agonist-sensitive and heterogeneous pools of phosphatidylinositol 4-phosphate in the plasma membrane. Biochim. Biophys. Acta *1858*, 1298–1305.

Zinser, E., Sperka-Gottlieb, C.D., Fasch, E.V., Kohlwein, S.D., Paltauf, F., and Daum, G. (1991). Phospholipid synthesis and lipid composition of subcellular membranes in the unicellular eukaryote Saccharomyces cerevisiae. J. Bacteriol. *173*, 2026–2034.

STAR★METHODS

KEY RESOURCES TABLE

REAGENT or RESOURCE	SOURCE	IDENTIFIER
Bacterial and Virus Strains		
BL21 (DE3)	New England BioLabs	C2527I
Rosetta (DE3) pLysS	Merck	70956
Chemicals, Peptides, and Recombinant Proteins		
Ethanolamine	ACROS Organics	149580010
Tween 80	SIGMA-ALDRICH	P1754
(Trimethylsilyl)diazomethane solution 2.0 M in hexanes	SIGMA-ALDRICH	362832
Formic acid eluent additive for LC-MS	SIGMA-ALDRICH	56302
ACQUITY UPLC Protein BEH C4 Column, 300Å, 1.7 µm, 1 mm X 100 mm	Waters	186005590
Yeast nitrogen base without amino acids, ammonium sulfate and inositol	FORMEDIUM	CYN3810
Casamino Acids	FORMEDIUM	CAS01
Myo-[2-H^3]-inositol	PerkinElmer	NET114A005MC
Perchloric acid	ACROS Organics	223312500
Glass beads	SIGMA	G1277
40% methylamine	SIGMA-ALDRICH	426466
1-Butanol	ACROS Organics	107690025
Diethyl-ether	ACROS Organics	176830010
Ethyl formate	ACROS Organics	150675000
Partisphere 5 µm SAX column	Hichrome	4621-1505
Ammonium phosphate, dibasic	ACROS Organics	201820025
Phosphoric acid	ACROS Organics	389020025
Ultima-Flo AP scintillation fluid	PerkinElmer	6013599
Malachite Green	SIGMA-ALDRICH	38800
Ammonium molybdate	SIGMA-ALDRICH	277908
Sulfuric acid	SIGMA-ALDRICH	339741
Complete EDTA-free protease inhibitor	Thermo Fisher Scientific	A32955
Dithiothreitol (DTT)	Thermo Fisher Scientific	R0861
AEBSF Protease Inhibitor	Thermo Fisher Scientific	78431
Glutathione Sepharose 4B	GE Healthcare	17-0756-01
PreScission protease	GE Healthcare	270843
Ni-NTA Agarose	QIAGEN	1018244
Imidazole	SIGMA ALDRICH	I202
IANBD-amide	Invitrogen	D2004
L-Cysteine Hydrochloride	SIGMA	C7477
NitroPure™, Nitrocellulose Transfer Membrane, 0.45 µm	GVS	1212602
SuperSignal™ West Pico Chemiluminescent Substrate	Thermo Scientific	10481755
Purified Mouse Anti-6xHis	BD Biosciences	552565
DP-PI4P (16:0/16:0)	CellSignals	912
Ergosterol	United States Biological	275432
Brain PI4P (L-α-phosphatidylinositol-4-phosphate)	Avanti Polar Lipids	840045X

(*Continued on next page*)

Continued

REAGENT or RESOURCE	SOURCE	IDENTIFIER
Brain PI(4,5)P_2 (L-α-phosphatidylinositol-4,5-bisphosphate)	Avanti Polar Lipids	840046X
Egg PC (L-α-phosphatidylcholine)	Avanti Polar Lipids	840051
Brain PS (L-α-phosphatidyserine)	Avanti Polar Lipids	840032
POPC (1-palmitoyl-2-oleoyl-sn-glycero-3-phosphocholine)	Avanti Polar Lipids	850457
Cholesterol	Avanti Polar Lipids	700000P
Oleic acid	Sigma-Aldrich	O1008
Egg PA (L-α-phosphatidic acid) (Egg, Chicken)	Avanti Polar Lipids	840101
Liver PI (L-α-phosphatidylinositol) (Liver, Bovine)	Avanti Polar Lipids	840042
POPE (2-Oleoyl-1-palmitoyl-*sn*-glycero-3-phosphoethanolamine)	Sigma	O1991
18:1 Dansyl PE (1,2-dioleoyl-sn-glycero-3-phosphoethanolamine-N-(5-dimethylamino-1-naphthalenesulfonyl)	Avanti Polar Lipids	810330
PO-PI4P (1-palmitoyl-2-oleoyl-sn-glycero-3-phospho-(1'-myo-inositol-4'-phosphate))	Avanti Polar Lipids	850157P
DPPS (1,2-dipalmitoyl-sn-glycero-3-[phospho-L-serine])	Avanti Polar Lipids	840037
POPS (1-palmitoyl-2-oleoyl-sn-glycero-3-[phospho-L-serine])	Avanti Polar Lipids	840034
DOPS (1,2-dioleoyl-sn-glycero-3-[phospho-L-serine])	Avanti Polar Lipids	840035
17:0-20:4 PI(4)P (1-heptadecanoyl-2-(5Z,8Z,11Z,14Z-eicosatetraenoyl)-sn-glycero-3-phospho-(1'-myo-inositol-4'-phosphate) (ammonium salt))	Avanti Polar Lipids	LM1901
17:0-20:4 PI(4,5)P_2 (1-heptadecanoyl-2-(5Z,8Z,11Z,14Z-eicosatetraenoyl)-sn-glycero-3-phospho-(1'-myo-inositol-4',5′-bisphosphate) (ammonium salt))	Avanti Polar Lipids	LM1904
17:0-20:4 PI(3,4,5)P_3 (1-heptadecanoyl-2-(5Z,8Z,11Z,14Z-eicosatetraenoyl)-sn-glycero-3-phospho-(1'-myo-inositol-3′,4',5′-trisphosphate) (ammonium salt))	Avanti Polar Lipids	LM1906
17:0-20:4 PI (1-heptadecanoyl-2-(5Z,8Z,11Z,14Z-eicosatetraenoyl)-sn-glycero-3-phospho-(1'-myo-inositol) (ammonium salt))	Avanti Polar Lipids	LM1502
17:0-20:4 PS (1-heptadecanoyl-2-(5Z,8Z,11Z,14Z-eicosatetraenoyl)-sn-glycero-3-phospho-L-serine (ammonium salt))	Avanti Polar Lipids	LM1302
Zirconia beads 5.0 mm	TOMY	ZB-50
Mini-extruder Set	Avanti Polar Lipids	610000
PC Membranes 0.1 μm	Avanti Polar Lipids	610005
PC Membranes 0.4 μm	Avanti Polar Lipids	610007
PC Membranes 1.00 μm	Avanti Polar Lipids	610010
Filter Supports	Avanti Polar Lipids	610014
Slide-A-Lyzer^{TM} Dialysis Cassettes, 3.5K MWCO	Thermo Fisher Scientific	66330
Propidium iodide	Invitrogen	P3566
Deposited Data		
Raw imaging data	This study	Mendeley Data: https://doi.org/10.17632/x96sprmwrg.1
Critical Commercial Assays		
ADP-Glo^{TM} Kinase Assay	Promega	V6930
Experimental Models: Organisms/Strains		
SEY6210 [*MATα leu2-3,112 ura3-52 his3Δ200 trp1-Δ901 lys2-801 suc2Δ9*]	PMID: 3062374	N/A
SEY6210.1 [*MAT**a** leu2-3,112 ura3-52 his3Δ200 trp1-Δ901 lys2-801 suc2Δ9*]	PMID: 3062374	N/A

(*Continued on next page*)

Continued

REAGENT or RESOURCE	SOURCE	IDENTIFIER
CBY886 (*osh1-7Δ/osh4*^{ts}) [*SEY6210 osh1Δ::kan-MX4 osh2Δ::kan-MX4 osh3Δ::LYS2 osh4Δ::HIS3 osh5Δ::LEU2 osh6Δ::LEU2 osh7Δ::HIS3 (osh4-1, URA3)*]	Beh and Rine, 2004	N/A
ANDY198 (Δtether) [*SEY6210.1 ist2Δ::HISMX6 scs2Δ::TRP1 scs22Δ::HISMX6 tcb1Δ::KANMX6 tcb2Δ::KANMX6 tcb3Δ::HISMX6*]	Manford et al., 2012	N/A
AAY104 (*pik1*^{ts}) [*SEY6210 pik1Δ::HIS3 carrying* pRS314*pik1-83* (*TRP1 CEN6 pik1-83)*]	Audhya et al., 2000	N/A
AAY102 (*stt4*^{ts}) [*SEY6210 stt4Δ::HIS3* carrying pRS415*stt4-4* (*LEU2 CEN6 stt4-4*)]	Audhya et al., 2000	N/A
AAY105 (*pik1*^{ts}/*stt4*^{ts}) [*SEY6210 stt4Δ::HIS3 pik1Δ::HIS3* carrying pRS415*stt4-4* (*LEU2 CEN6 stt4-4*) and pRS314*pik1-83* (*TRP1 CEN6 pik1-83*)]	Audhya et al., 2000	N/A
AAY202 (*mss4*^{ts}) [SEY6210 *mss4Δ::HIS3MX6* carrying pYCplac111 *mss4-102 (LEU2 CEN6 mss4-102)*]	https://doi.org/10.1091/mbc.01-10-0476	N/A
W303-1A [*MATα leu2-3,112 trp1-1 can1-100 ura3-1 ade2-1 his3-11,15*]	PMID: 2645056	N/A
cho1Δ [*W303-1A cho1Δ::URA3*]	https://doi.org/10.1021/bi300086c	N/A
YTN1 [*W303-1A ADE2::URA3*]	This study	N/A
YTN3 [*W303-1A cho1Δ::URA3 ADE2::URA3*]	This study	N/A
GL7 [*X2180**a** gal2 erg12-1 hem3-6*]	PMID: 323256	N/A
YTN39 [*X2180**a** gal2 erg12-1 hem3-6 ura3Δ:: KANMX6*]	This study	N/A
ole1Δ [*BY4741 ole1Δ::CgHIS3*]	https://doi.org/10.1242/bio.022053	N/A
Recombinant DNA		
pRS424-*P*^{PRC1}-GFP-2xPH$_{PLCδ}$	This study	N/A
pRS415-*P*^{GPD}-mCherry-2xPH$_{PLCδ}$	https://doi.org/10.1038/emboj.2012.127	N/A
pRS426-*P*^{PRC1}-GFP-2xPH$_{PLCδ}$	https://doi.org/10.1091/mbc.01-10-0476	N/A
pRS314-*P*^{GPD}-GFP-C2$_{Lact}$	Yeung et al., 2008	N/A
pRS416-*P*^{GPD}-GFP-C2$_{Lact}$	Yeung et al., 2008	N/A
pRS414-*P*^{GPD}-GFP-P4C$_{SidC}$	This study	N/A
pRS416-*P*^{GPD}-GFP-P4C$_{SidC}$	https://doi.org/10.1371/journal.ppat.1004965	N/A
pEGFP-N1:GCC$_{GMAP210(39\text{-}377aa)}$	Horchani et al., 2014	N/A
pRS424-*P*^{GPD}-5K$_{loop}$-GCC$_{GMAP210}$-GFP	This study	N/A
pRS426-*P*^{GPD}-5K$_{loop}$-GCC$_{GMAP210}$-GFP	This study	N/A
pET21b+: *zPIP5K1Aα*$_{(49\text{-}431aa)}$	Hu et al., 2015	N/A
pRS416-*P*^{GPD}-*zPIP5K1Aα*$_{(49\text{-}431aa)}$	This study	N/A
pRS414-*P*^{MSS4}-*MSS4*-GFP	https://doi.org/10.1038/emboj.2012.127	N/A
pRS416-*P*^{MSS4}-*MSS4*-GFP	https://doi.org/10.1038/emboj.2012.127	N/A
pRS416-*P*^{MSS4}-*mss4* K571A-GFP	This study	N/A
pRS416-*P*^{MSS4}-*mss4* K720D/K721D-GFP	This study	N/A
pRS416-*P*^{MSS4}-*mss4* L722K/L729K-GFP	This study	N/A
pRS414-*P*^{PIL1}-*PIL1*-GFP	Karotki et al., 2011	N/A
pRS314-*P*^{OSH4}-*OSH4*	Beh and Rine, 2004	N/A
pRS314-*P*^{OSH4}-*osh4* G183D (*ts* mutant)	Beh and Rine, 2004	N/A
pRS314-*P*^{OSH4}-*osh4* H143A/H144A	This study	N/A
pRS314-*P*^{OSH4}-*osh4Δ29*	This study	N/A
pRS414-*P*^{GPD}-*OSH6*	This study	N/A
pRS414-*P*^{GPD}-*osh6* H157A/H158A	This study	N/A
pGEX6P-1:PH$_{PLCδ}$ V58C	This study	N/A

(*Continued on next page*)

Continued

REAGENT or RESOURCE	SOURCE	IDENTIFIER
pGEX6P-1:CFP-C2$_{Lact}$	This study	N/A
pGEX6P-1:Venus-C2$_{Lact}$	This study	N/A
pGEX6P-1:Venus-P4C$_{SidC}$	This study	N/A
pGEX6P-1:*MSS4*$_{(347-779aa)}$-his$_6$	This study	N/A
pGEX6P-1:*mss4*$_{(347-779aa)}$ K571A-his$_6$	This study	N/A
pGEX6P-1: *mss4*$_{(347-779aa)}$ K720D/K721D-his$_6$	This study	N/A
pGEX6P-1: *mss4*$_{(347-779aa)}$ L722K/L729K-his$_6$	This study	N/A
Software and Algorithms		
Fiji/ImageJ	Fiji	RRID: SCR_002285
Adobe Photoshop CS6 extended	Adobe	RRID: SCR_014199
GraphPad Prism 6	GraphPad Software	RRID: SCR_002798
RStudio	RStudio	RRID: SCR_000432
HeliQuest	CNRS	http://heliquest.ipmc.cnrs.fr/
PyMOL	Schrodinger	RRID: SCR_000305
PEP-FOLD 3	RPBS	http://bioserv.rpbs.univ-paris-diderot.fr/services/PEP-FOLD3/
VMD	University of Illinois<br>https://doi.org/10.1016/0263-7855(96)00018-5	https://www.ks.uiuc.edu/Research/vmd/
GROMACS	European Research Council<br>https://doi.org/10.1016/j.softx.2015.06.001)	RRID: SCR_014565
MDAnalysis	https://doi.org/10.25080/majora-629e541a-00e<br>https://doi.org/10.1002/jcc.21787	https://www.mdanalysis.org/
NumPy	http://SciPy.org<br>https://doi.org/10.1109/MCSE.2011.37	RRID: SCR_008633
SciPy	http://SciPy.org	RRID: SCR_008058
IPython	http://ipython.org<br>https://doi.org/10.1109/MCSE.2007.53	RRID: SCR_001658
Matplotlib	http://SciPy.org<br>https://doi.org/10.1109/MCSE.2007.55	RRID: SCR_008624
UCSF CHIMERA	UCSF<br>https://doi.org/10.1002/jcc.20084	RRID: SCR_004097
CHARMM-GUI	CHARMM<br>https://doi.org/10.1021/acs.jctc.5b00935	http://www.charmm-gui.org/
CHARMM36m	NIH (Huang et al., 2017)	http://mackerell.umaryland.edu/charmm_ff.shtml
Martini v2.2 force field	https://doi.org/10.1021/jp071097f<br>https://doi.org/10.1021/ct300646g	http://cgmartini.nl/index.php/224-m22
Total Chrome Navigator software	PerkinElmer	https://www.perkinelmer.com/
Analyst	Sciex	https://sciex.com
MultiQuant	Sciex	https://sciex.com

LEAD CONTACT AND MATERIALS AVAILABILITY

Further information and requests for resources and reagents should be directed to and will be fulfilled by the Lead Contact, Christopher J. Stefan (c.stefan@ucl.ac.uk).

EXPERIMENTAL MODEL AND SUBJECT DETAILS

Yeast

Descriptions of *Saccharomyces cerevisiae* strains used in this study are in the Key Resources Table. Standard techniques were used for yeast growth. For *cho1*Δ cells, the medium was supplemented with 1 mM ethanolamine. For the *GL7* strain (provided by Dr. W. David Nes, Texas Tech Univ.), 2 mg/ml ergosterol solubilized in a Tween 80/ethanol solution (1:4, w/w) was added to the medium as a 0.5% solution for a final concentration of 10 μg/ml. The *URA3* gene in the *GL7* strain was deleted by homologous recombination. For *ole1*Δ cells (provided by Dr. Takeshi Noda, Osaka Univ.), 0.5% casamino acids were added to support growth of the *ole1*Δ mutant cells in synthetic defined medium. Oleic acid at a final concentration of 1 mM and 1% (v/v) Triton X-100 were also added to the culture medium.

METHOD DETAILS

Plasmids

Descriptions of plasmids used in this study are in the Key Resources Table. DNA sequences encoding yPIP5K/Mss4 fragment (aa 347-779, NP_010494) were amplified by PCR and subcloned into pGEX-6P-1 vector together with His-Tag. yPIP5K K571A, K720D/K721D, and L722K/L729K were generated by inverse PCR. $C2_{Lact}$ and P4C cDNAs were amplified by PCR and subcloned into pGEX-6P-1 vector together with CFP and/or Venus. cDNA encoding zPIP5K (aa 49-431, NP_001018438) (provided by Dr. Ya Ha, Yale School of Medicine) was subcloned into pRS416 plasmid. zPIP5K fragment (aa 380-403) fused to GMAP210 coiled-coil region (aa 39-377) (provided by Dr. Bruno Antonny, CNRS Université de Nice Sophia Antipolis) was cloned into pRS424 or pRS426 plasmid together with EGFP. Osh4 wild-type, G183D (*ts* mutant), Δ29, and H143A/H144A cDNAs were subcloned into pRS314 plasmid. Osh6 wild-type and H157A/H158A cDNAs (provided by Dr. Guillaume Drin, CNRS Université de Nice Sophia Antipolis) were subcloned into the plasmid pRS414. GFP-P4C and $2xPH_{PLC\delta}$ were subcloned into pRS414 or pRS424, respectively.

Fluorescence Microscopy

Images were obtained with a 100 × CFI Plan Apochromat VC oil-immersion objective lens (1.4 NA), using a Perkin-Elmer Ultraview VOX spinning disk confocal microscope that consists of a Nikon TiE inverted stand attached to Yokogawa CSU-X1 spinning unit and a Hamamatsu C9100-13 EMCCD camera with a pixel size of 16 μm. All images were collected as square images with 512 × 512 pixels. For the final output, images were processed using Fiji/ImageJ and Adobe Photoshop CS6 extended software. Plasma membrane (PM) relative fluorescence (relative F_{PM}) was quantified as described below. Briefly, single channel images were created by splitting channels. Single cell images were randomly chosen from the single channel images, duplicated and saved. Three lines (crossing over, inside and outside a cell) were drawn on the single cell images, added to ROI manager, and analyzed by 'Multi Plot' module in Fiji/ImageJ to obtain fluorescence intensity profiles. These values, related to the intensity values for each pixel, were imported into Excel. We then calculated the average values of fluorescence intensity profiles on drawn lines inside and outside a cell, and expressed as F_{in} and F_{out}, respectively. The specific cytosolic signal (F_{cyto}) was calculated by subtracting F_{out} from F_{in}. In order to obtain PM fluorescence intensity (F_{PM}), the two outer cellular intensity peaks were found on the line crossing over a cell. The average value of intensity of those two peaks, one pixel in front and behind peaks was calculated (F_{cross}), and then subtracted F_{out} from F_{cross}. F_{PM} was divided by F_{cyto} to calculate relative F_{PM}. Taken together, PM relative fluorescence was calculated by using this equation: relative $F_{PM} = F_{PM}/F_{cyto} = (F_{cross}-F_{out})/(F_{in}-F_{out})$. Finding peaks in intensity profiles and calculations were automatically processed with an Excel VBA macro.

Analysis of ^{3}H-labeled Inositol Phosphates by HPLC

PIPs levels were analyzed as previously described (Stefan et al., 2011). Briefly, 5 OD_{600} units of cells cultured in YND media were washed by media lacking inositol and pre-incubated at 26°C or 38°C for 15 min. The cells were labeled with 50 μCi of myo-[2-H^3]-inositol in media lacking inositol and further incubated for 1 hour. Then, the cells were lysed in 4.5% perchloric acid with glass beads to generate extracts. After washed by 0.1 M EDTA, the extracts were mixed with methylamine reagent (methanol/40% methylamine/water/1-butanol; 4.6:2.6:1.6:1.1 v/v) and incubated at 53°C for 1 h to deacylate phospholipids. Samples were dried in a vacuum chamber, washed with water, dried again, and resuspended in 300 μl water. Extraction reagent (1-butanol/ethl-ether/formic acid ethyl ester; 20:4:1 v/v) was added and [^{3}H] glycerol-PIPs were separated into the aqueous phase by vortexing and centrifugation at 14,000 x *g* for 5 min. The extraction was repeated twice and the final aqueous phase was collected and dried. Dried pellets were resuspended in 260 μl water and separated on a Partisphere 5 μm SAX column attached to a PerkinElmer Series 200 HPLC system and a radiomatic 150TR detector using Ultima-Flo AP scintillation fluid. The HPLC and on-line detector were controlled with Total Chrome Navigator software. The data were analyzed using Total Chrome Navigator software.

LC-MS/MS analysis of methylated PIPs and PS

20 OD_{600} units of cells were precipitated and washed with cold 4.5% perchloric acid. For phosphoinositide measurements, cells were resuspended in 500 μL 0.5 M HCl and disrupted with a 5.0 mm zirconia bead by vigorous shaking (1,500 rpm for 10 min) using Shake Master Neo (BMS, Tokyo, Japan). The homogenates were transferred to new tubes and centrifuged at 15,000 × *g* for 5 min. The pellets were resuspended in 170 μL water and 750 μL of $CHCl_3$/MeOH/1 M HCl (2:1:0.1, v/v) and incubated for 5 min at room

temperature. To each sample 725 μL of $CHCl_3$ and 170 μL of 2 M HCl were added, followed by vortexing. After centrifugation at 1,500 × *g* for 5 min, the lower phase was collected and washed with 780 μL of pre-derivatization wash solution (the upper phase of $CHCl_3$/MeOH/0.01 M HCl (2:1:0.75 v/v)). The lipid extracts were derivatized by adding 50 μL of 2 M TMS-diazomethane in hexane. The derivatization was carried out at room temperature for 10 min and was stopped by adding 6 μL of glacial acetic acid. The derivatized samples were washed twice with 700 μL of post-derivatization wash solution (the upper phase of $CHCl_3$/MeOH/water (2:1:0.75 v/v)). After adding 100 μL of MeOH/H_2O (9:1, v/v), the samples were dried under a stream of N_2, dissolved in 80 μL of MeOH and sonicated briefly. After adding 20 μL of water, the samples were subjected to LC-ESI-MS/MS analysis. The LC-ESI-MS/MS analysis was performed on a Shimadzu Nexera ultra high performance liquid chromatography system coupled with a QTRAP 4500 hybrid triple quadrupole linear ion trap mass spectrometer. Chromatographic separation was performed on an Acquity UPLC C4 BEH column (100 mm × 1 mm, 1.7 μm; Waters) maintained at 40°C using mobile phase A (water containing 0.1% formate) and mobile phase B (acetonitrile containing 0.1% formate) in a gradient program (0–5 min: 45% B; 5–10 min: 45% B → 100% B; 10-15 min: 100% B; 15–16 min: 100% B → 45% B; 16-20: 45% B) with a flow rate of 0.1 mL/min. The instrument parameters for positive ion mode were as follows: curtain gas, 10 psi; collision gas, 7 arb. unit; ionspray voltage, 4500 V; temperature, 600°C; ion source gas 1, 30 psi; ion source gas 2, 50 psi; declustering potential, 121 V; entrance potential, 10 V; collision energy, 39 V; collision cell exit potential, 10 V. Methylated phosphoinositides and phosphatidylserine were identified and quantified by multiple reaction monitoring. For these measurements, an internal standard of 10 ng of 17:0-20:4 PIP was added to each sample.

Quantitative shotgun lipid MS data acquisition, analysis, and post-processing

The glycerolipid compositions of wild-type, *osh1-7Δ/osh4^{ts}* and Δtether mutant cell extracts shown in the Supplemental Information were determined by mass spectrometry-based quantitative, shotgun lipidomics by Lipotype GmbH (Dresden, Germany) as described (Ejsing et al., 2009; Klose et al., 2012). Total yeast cell lysate samples were diluted to 0.2 OD units using 155 mM ammonium bicarbonate in water to the total volume of 150 μl and were spiked with internal lipid standard mixture. Lipids were extracted using a two-step chloroform/methanol procedure with 750 μl volume of each organic phase step (chloroform:methanol, 15:1 and 2:1 respectively for the 1st and the 2nd step)(Ejsing et al., 2009). After extraction, the organic phase was transferred to an infusion plate and dried in a speed vacuum concentrator. 1st step dry extract was re-suspended in 100 μl 7.5 mM ammonium acetate in chloroform/methanol/propanol (1:2:4, V:V:V) and 2nd step dry extract in 100 μl 33% ethanol solution of methylamine in chloroform/methanol (0.003:5:1; V:V:V). All liquid handling steps were performed using Hamilton Robotics STARlet robotic platform with the Anti Droplet Control feature for organic solvents pipetting.

Samples were analyzed by direct infusion on a QExactive mass spectrometer (Thermo Scientific) equipped with a TriVersa NanoMate ion source (Advion Biosciences). Samples were analyzed in both positive and negative ion modes with a resolution of $R_{m/z = 200}$ = 280000 for MS and $R_{m/z = 200}$ = 17500 for MSMS experiments, in a single acquisition. MSMS was triggered by an inclusion list encompassing corresponding MS mass ranges scanned in 1 Da increments (Surma et al., 2015). Both MS and MSMS data were combined to monitor EE, DAG and TAG ions as ammonium adducts; PC as an acetate adduct; and PA, PE, PG, PI and PS as deprotonated anions.

Data were analyzed with in-house developed lipid identification software based on LipidXplorer (Herzog et al., 2012; Herzog et al., 2011). Data post-processing and normalization were performed using an in-house developed data management system. Only lipid identifications with a signal-to-noise ratio > 5, and a signal intensity 5-fold higher than in corresponding blank samples were considered for further data analysis.

Protein purification

Eschericia coli strains BL21 or Rosetta pLysS were used as a host cell line. Expression of recombinant protein was induced with 0.1-1 mM IPTG at 22°C or 37°C. The cell pellets were collected and resuspended in ice-cold homogenization buffer (50 mM Tris-HCl pH 6.8, 300 mM NaCl, 1 mM dithiothreitol (DTT), 0.1 mM AEBSF, and complete EDTA-free protease inhibitor). Cells were then disrupted by sonication in ice-cold homogenization buffer. The homogenized cells were centrifuged at 20,800 × *g* for 30 min to remove cell debris. GST recombinant proteins were purified with glutathione-Sepharose and cleaved from GST by using 0.1 U/μl PreScission protease. Untagged proteins were dialyzed with dialysis buffer (50 mM Tris-HCl pH 6.8, 150 mM NaCl, and 1 mM DTT) three times and then dialyzed with storage buffer (50 mM Tris-HCl pH 6.8, 150 mM NaCl, 2 mM DTT, and 50% glycerol) and stored at −80°C before analysis. For purification of His-tagged zPIP5K1Aα (49-431aa), 10 mM imidazole was additionally added to homogenization buffer to reduce non-specific binding. His-tagged recombinant proteins were purified with Ni-NTA Agarose, eluted by 80 mM and 160 mM imidazole and dialyzed as shown above.

Preparation of NBD-$PH_{PLC\delta}$ Proteins

Rat PLCδ (NP_058731, 11-140 aa) cDNA (1x $PH_{PLC\delta}$) was amplified by PCR and subcloned into pGEX-6P-1. $PH_{PLC\delta\ V58C}$/pGEX6P-1 was prepared by introducing point mutations for NBD labeling. GST-$PH_{PLC\delta\ V58C}$ protein was purified as described above. After cleavage of GST tag, untagged $PH_{PLC\delta\ V58C}$ was dialyzed in TBS (50 mM Tris-HCl pH 6.8, and 150 mM NaCl) three times to remove DTT, and then labeled with a 10-fold excess of IANBD-amide. After overnight incubation at 4°C, the reaction was stopped with 4 mM cysteine and residual IANBD-amide was removed by dialysis (50 mM Tris-HCl pH 6.8, 150 mM NaCl, and 1 mM DTT). NBD-labeled proteins were mixed with equal volume of glycerol and stored at −80°C before analysis.

Liposome preparations

Lipids were mixed at the desired molar ratio and the organic solvent was removed in a rotary evaporator. The lipid films were hydrated in buffer A (50 mM Tris-HCl pH 7.5, 150 mM NaCl, 1 mM DTT, 0.2 mM EDTA, and 1 mM EGTA) for 30 min at room temperature or at 65°C for liposomes containing saturated lipids. The suspensions were sonicated in a bath sonicator or extruded through polycarbonate filters of the indicated pore size using a mini-extruder (Avanti Polar Lipids). Liposomes were used within 1 day.

Real-time PIP 5-kinase assay

PIP 5-kinase reactions were carried out in buffer A (50 mM Tris-HCl pH 7.5, 150 mM NaCl, 1 mM DTT, 0.2 mM EDTA, and 1 mM EGTA) supplemented with 2 mM $MgCl_2$ and 80 μM ATP and measured with a Fluoromax spectrometer (HORIBA Scientific). Briefly, the sample (150 μl) containing liposomes (400 μM total lipids) were mixed with PIP5K (final conc. 60 nM except as indicated in Figure S2C and S3G-H) and NBD-$PH_{PLC\delta}$ (final conc. 400 nM) in a 200 μL quartz cell. After 5 min, 15 μL of $MgCl_2$ was added (final conc. 2 mM). At 2 min later, the reaction was initiated by adding 15 μL of ATP solution (final conc. 80 μM). NBD fluorescence (ex/em 468 nm/530 nm) was recorded every second. The excitation and emission slits were set at 5 nm bandwidths. We calculated an increase in signal of NBD fluorescence ($\Delta Em_{530,\ raw\ data}$) from that measured before ATP addition. To subtract the contribution of liposomes alone, a background signal of NBD fluorescence ($\Delta Em_{530,\ BG}$) was measured with the NBD-$PH_{PLC\delta}$ in the presence of 0% PI4P liposomes. Finally, an NBD signal increase dependently of PIP5K reaction was calculated by using this equation: $\Delta Em_{530} = \Delta Em_{530,\ raw\ data} - \Delta Em_{530,\ BG}$. Data were analyzed by using RStudio or an Excel VBA macro. To determine initial velocities of PIP5K reaction at different substrate concentrations, ΔEm_{530} data were analyzed by using GraphPad Prism software and a slope of the initial portion of the regression curve was used as an individual initial velocity. Then, initial velocities of PIP5K reaction were fitted to an allosteric sigmoidal kinetic model to obtain the apparent K_m and V_{max} values by using Graphpad Prsim software.

ADP-Glo kinase assay

PIP 5-kinase reactions were carried out as in the real-time assays except with 8 μM ATP. ADP-Glo assays were performed according to the manufacturer's instructions. Briefly, a part of reaction mixture (25 μl) was mixed with 25 μL of ADP-Glo reagent. After a 40 min of incubation, 50 μL of Kinase Detection Reagent was added and further incubated for 30 min. Luminescence signal was recorded using a Tecan infinite F200 microplate reader. Non-specific background signals independent of PIP5K activity were subtracted by using liposomes lacking PI4P.

Malachite green phosphate assay

A malachite-molybdate solution was made freshly by mixing a malachite green solution (0.03175% malachite green and 0.35% polyvinyl alcohol) and molybdate solution (3.46% ammonium molybdate and 11.2% (v/v) concentrated sulfuric acid) at a ratio of 64 to 86. To measure free phosphate levels during the PIP5K reaction course, 100 μL of PIP5K reaction mixture was mixed with 150 μL of malachite-molybdate solution. Absorbance at 595 nm was measured using a Tecan infinite F200 microplate reader. Phosphate levels were determined using a phosphate standard.

Liposome sedimentation assay

Sedimentation assays were carried out in buffer A (50 mM Tris-HCl pH 7.5, 150 mM NaCl, 1 mM DTT, 0.2 mM EDTA, and 1 mM EGTA) supplemented with 2 mM $MgCl_2$. Briefly, liposomes of defined compositions (400 μM total lipids) were mixed with zPIP5K (final conc. 130 nM). After 15 min at room temperature, the mixture was centrifuged at 20,800 x *g* for 30 min. The supernatant and pellet fractions were solubilized with SDS-PAGE sample buffer and heated at 95°C for 5 min. The supernatant and pellet fractions were separated by SDS-PAGE and transferred to nitrocellulose membranes. Immunoblot analysis was performed with an anti-His antibody and visualized with Super-Signal West Pico Chemiluminescent substrate to detect the zPIP5K-His protein. Signal intensities were analyzed using the LAS-4000mini image analyzer and Fiji/ImageJ.

in vitro FRET assays

Liposomes (400 μM total lipids) were mixed with CFP-$C2_{Lact}$ (400 nM) and Venus-P4C (400 nM) in buffer A (50 mM Tris-HCl pH 7.5, 150 mM NaCl, 1 mM DTT, 0.2 mM EDTA, and 1 mM EGTA) supplemented with 2 mM $MgCl_2$. After incubation for 5 min, fluorescence spectra were recorded using a Fluoromax spectrometer. CFP signal (ex/em 433 nm/476 nm), Venus signal (ex/em 505 nm/ 528 nm), and FRET signal (ex/em 433 nm/528 nm) were measured. The excitation and emission slits were set at 5 nm bandwidths. We analyzed CFP or Venus proteins alone at several different concentrations and found that 42.38% of CFP signal and 3.206% of Venus signal bled through into the FRET signal in our experimental conditions. Based on those results, corrected FRET values were calculated using the following equation: cFRET = FRET – 0.4238*CFP – 0.03206*Venus.

Quantitative membrane binding assay using Dansyl-PE liposomes

Liposomes (400 μM total lipids) containing 2 mol% Dansyl-PE were mixed with Venus-$C2_{Lact}$ (400 nM) or Venus-P4C (1 μM) in buffer A (50 mM Tris-HCl pH 7.5, 150 mM NaCl, 1 mM DTT, 0.2 mM EDTA, and 1 mM EGTA) supplemented with 2 mM $MgCl_2$. After incubation for 5 min, fluorescence spectra were recorded using a Fluoromax spectrometer. Tryptophan (Trp; ex/em 280 nm/350 nm), Dansyl (ex/em 330 nm/ 510 nm), and FRET signals (ex/em 280 nm/500 nm) were measured. The excitation and emission slits were set at 5 nm

bandwidths. We analyzed Venus-C2$_{Lact}$, Venus-P4C or Dansyl-PE liposomes alone at several different concentrations and calculated a background signal and amount of bleed-through signals into the FRET signal in our experimental conditions. Based on those results, corrected FRET values were calculated using the following equation: cFRET = FRET – 0.0678*Trp – 0.3316*Dansyl – 1674, for Venus-C2$_{Lact}$; cFRET = FRET – 0.2195*Trp – 0.3316*Dansyl – 1674, for Venus-P4C.

Plasma membrane integrity assays

Yeast strains were grown to midlog phase at 26°C and shifted to 42°C for 15 min as indicated. 1 OD_{600} equivalent of cells was pelleted and resuspended in PBS, and cells were stained with propidium iodide for 15 min. Cells were then washed twice with ddH_2O and analyzed by flow cytometry (BD Accuri C6). For each condition, 50,000 cells were counted in duplicate in three independent experiments. Background was determined by analyzing each of the cell strains at the indicated temperatures prior to staining with propidium iodide.

Atomistic simulations

We modeled a 24-mer peptide (LQSYRLVKKLEHSWKALLHDGDTV) from the phsophatidylinositol-4-phosphate 5 kinase (Q503I3) into a α-helical conformation using the UCSF CHIMERA software package and aligned it along the x axis, centered at the origin. We used CHARMM-GUI to set up the atomistic plasma membrane model systems with CHARMM36m (Huang et al., 2017). All simulations were run using the GROMACS 2018 software package. N- and C-termini were neutralized (residue types NNEU and CNEU, respectively), whereas H391 and H398 were protonated. We embedded the peptide into the lipid headgroup region by translating it by 16 Å along the z axis. Ions were added to a total concentration of 150 mM NaCl, after neutralizing the system with counter ions. The resulting membrane containing cholesterol has the following molar composition (the number in brackets reports the number of molecules for each lipid species): 69% mol POPC [414], 20% mol cholesterol [120], 10% mol DOPS [60], 1% mol polyunsaturated bPI4P (SA-PI4P) [6]. We energy minimized all systems with steepest descent and performed equilibration in subsequent steps. First the system was equilibrated in a canonical (NVT) ensemble with an integration time step of 1 fs for 25 ps, maintaining a constant temperature of 310 K with the Berendsen thermostat. In this phase, position restraints of 1000 kJ $mol^{-1}nm^{-2}$ were applied to all lipid heavy atoms, whereas restraints on protein backbone heavy atoms were lowered from 4000 to 2000 kJ $mol^{-1}nm^{-2}$. The Berendsen barostat was used to keep a constant pressure of 1 bar in the isothermal isobaric (NPT) equilibration for the first 25 ps with a time step of 1 fs (Berendsen et al., 1984). All subsequent equilibration steps were performed for 300 ps with a time step of 2 fs. Here, lipid position restraints were decreased from 400 to 0 kJ $mol^{-1}nm^{-2}$ and protein position restraints were decreased from 1000 to 200 kJ $mol^{-1}nm^{-2}$. No restraints were applied on the systems during the production simulations. The temperature and pressure were kept constant at 310 K and 1 bar using the Velocity Rescale thermostat (Bussi et al., 2007) and Parrinello-Rahman barostat (Parrinello and Rahman, 1980) with a semiisotropic pressure coupling, applying each on the protein, solvent and membrane with characteristic times of 1 and 5 ps, respectively. All non-bonded interactions were cutoff at 1.2 nm throughout simulations.

Analysis of lipid localization

We discretized the position of each lipid on a two-dimensional grid by using the x and y coordinates of phosphate moieties (phospholipids) or oxygen atoms (cholesterol), using a bin width of 1 Å and sampling every 250 ps. We used an in-plane least-square fit of the protein backbone C atoms of the 5K$_{loop}$ helical peptide to a reference structure aligned along the x axis, to obtain a two-dimensional in-place rotational angle for every frame. The two-dimensional grid was rotated by the calculated angle about the axis orthogonal to the lipid bilayer plane and interpolated using a third order spline. The grid was extended and cropped after the rotation in every frame, taking into account periodic boundary conditions, to keep the original dimensions. Finally, we obtained the reported lipid localization densities by averaging all two-dimensional grids along the entire trajectory ignoring the first 100 ns.

Trajectory analysis

We used VMD, GROMACS, MDAnalysis, NumPy, SciPy, IPython, and Matplotlib for the analysis and visualization of trajectories.

QUANTIFICATION AND STATISTICAL ANALYSIS

Statistical analysis was carried out using GraphPad Prism 6. To compare the mean of two groups, an unpaired two-tailed t test was used. To compare the mean of multiple groups, we used one-way ANOVA followed by Tukey-Kramer multiple comparisons.

DATA AND CODE AVAILABILITY

The raw imaging data have been deposited in Mendeley Data and can be accessed: https://doi.org/10.17632/x96sprmwrg.1.

Molecular Cell, Volume *75*

Supplemental Information

Osh Proteins Control Nanoscale Lipid Organization Necessary for PI(4,5)P_2 Synthesis

Taki Nishimura, Michael Gecht, Roberto Covino, Gerhard Hummer, Michal A. Surma, Christian Klose, Hiroyuki Arai, Nozomu Kono, and Christopher J. Stefan

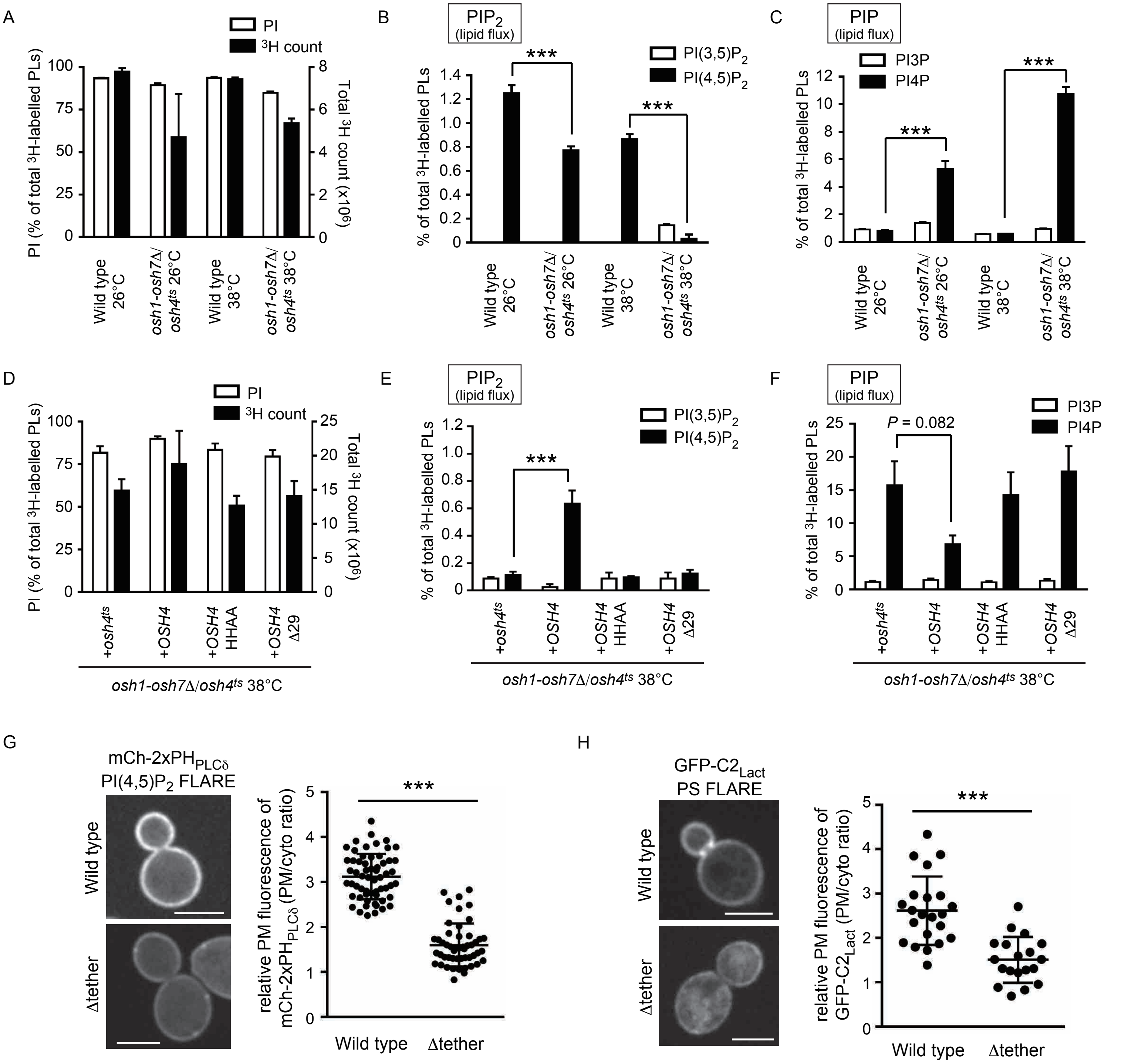

Figure S1. Reduction of PI(4,5)P_2 and PS levels at the PM of *osh1-7Δ/osh4^{ts}* and Δtether mutants, related to Figure 1 and the Supplemental Data Set

(A-C) Measurements of phosphatidylinositol (PI) and phosphoinositide lipid metabolism by ^{3}H-inositol labeling and HPLC analysis of wild type and *osh1-7Δ/osh4^{ts}* cells cultured at 26ºC or 38ºC for 1 h. Data represent mean ± SEM (n = 3). (D-F) Measurements of phosphatidylinositol (PI) and phosphoinositide lipid metabolism by ^{3}H-inositol labeling and HPLC analysis of *osh1-7Δ/osh4^{ts}* cells expressing wild type or mutant forms of Osh4 cultured at 38ºC for 1 h. Data represent mean ± SEM (n = 3). (G, H) PI(4,5)P_2 (mCherry-2xPH$_{PLCδ}$) and PS (GFP-C2$_{Lact}$) FLARE localization in wild type and Δtether cells. Data represent mean ± SD; (G) n≧50 cells, (H) n≧19 cells. Scale bars, 4 µm. ***P < 0.001.

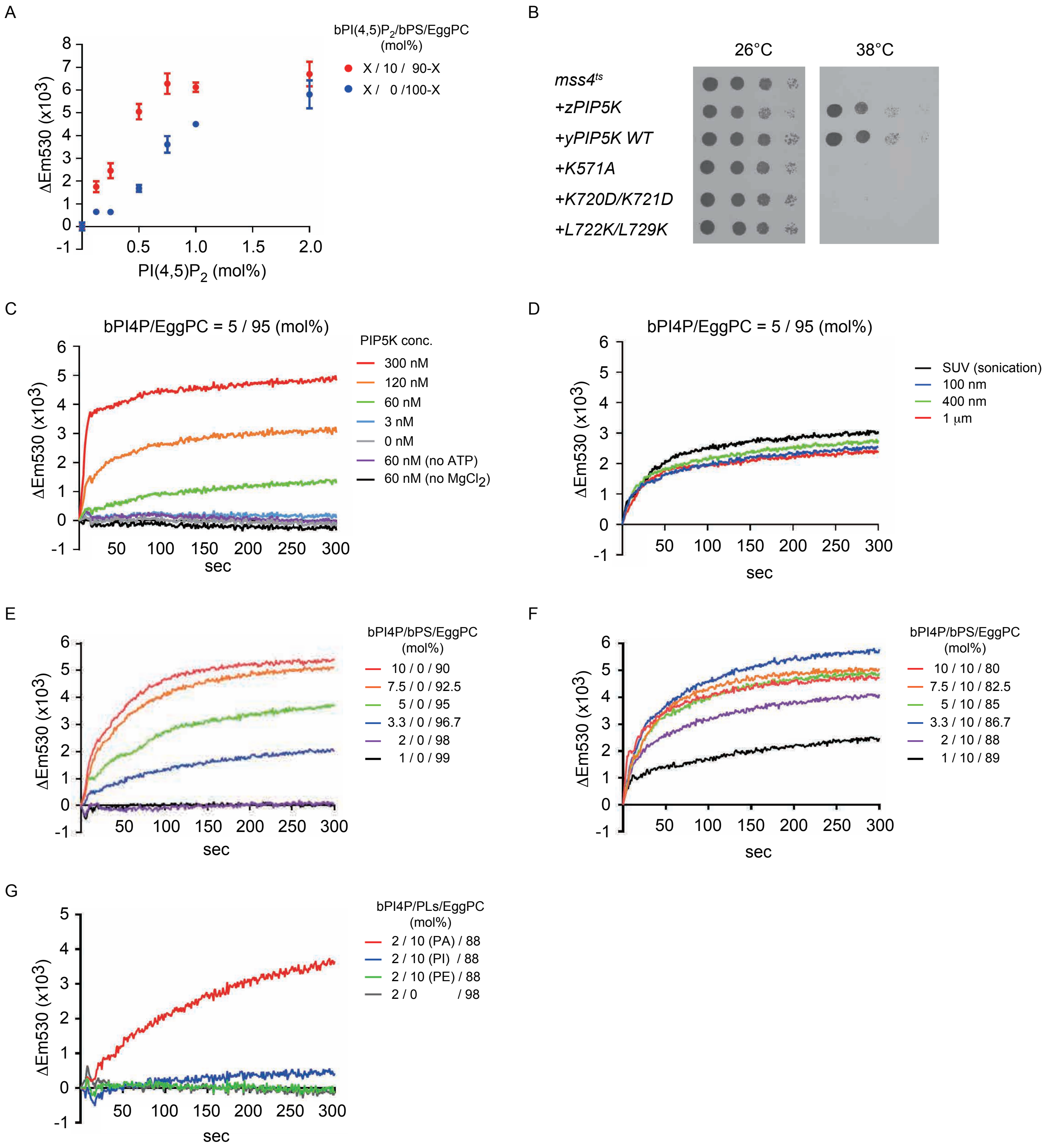

Figure S2. PS stimulates zPIP5K activity *in vitro*, related to Figure 2

(A) Changes in NBD-PH$_{PLC\delta}$ fluorescence intensity in response to PI(4,5)P$_2$ levels on liposomes. Liposomes were incubated with 400 nM NBD-PH$_{PLC\delta}$ for 5 min and NBD fluorescence was recorded by fluorescence spectroscopy. Data represent mean ± SEM (n=3). (B) Growth assay of $mss4^{ts}$ mutant cells. A zebrafish PIP5K catalytic domain (zPIP5K), a full-length yPIP5K wild-type (yPIP5K WT), a kinase dead mutant (K571A), a negatively charged mutant (K720D/K721D), an amphipathic property mutant (L722K/L729K) or empty pRS416 plasmid was expressed in $mss4^{ts}$ mutant cells as indicated. Serial dilutions of the cells were grown on –Ura plates to retain the plasmids at either 26ºC or 38ºC for 4 days. (C) Increase of NBD-PH$_{PLC\delta}$ fluorescence intensity reflects PI(4,5)P$_2$ generation by the PIP 5-kinase (PIP5K) reaction. Real-time PIP5K assays were performed as described in the STAR Methods. Note that zPIP5K activity was undetectable in the absence of zPIP5K, ATP, and MgCl$_2$. Data represent mean values (n=3; SEM<0.36x10^3). (D) zPIP5K showed minimal sensitivity to differences in size of liposomes containing 5 mol% bPI4P. The indicated sizes of liposomes were obtained by extrusion. Small unilamellar vesicles (SUV) were prepared by sonication. Data represent mean values (n≧3; SEM<0.44x10^3). (E, F) PIP5K activity against liposomes containing different concentrations of brain PI4P. Liposomes containing 0 mol% bPS (E) or 10 mol% bPS (F) were analyzed. (E, F) Data represent mean values (n≧3; SEM<1.07x10^3). (G) The effect of phosphatidic acid (PA), phosphatidylinositol (PI) and phosphatidylethanolamine (PE) on PIP5K activity in a real-time PIP5K assay. Data represent mean values (n=3; SEM<0.64x10^3).

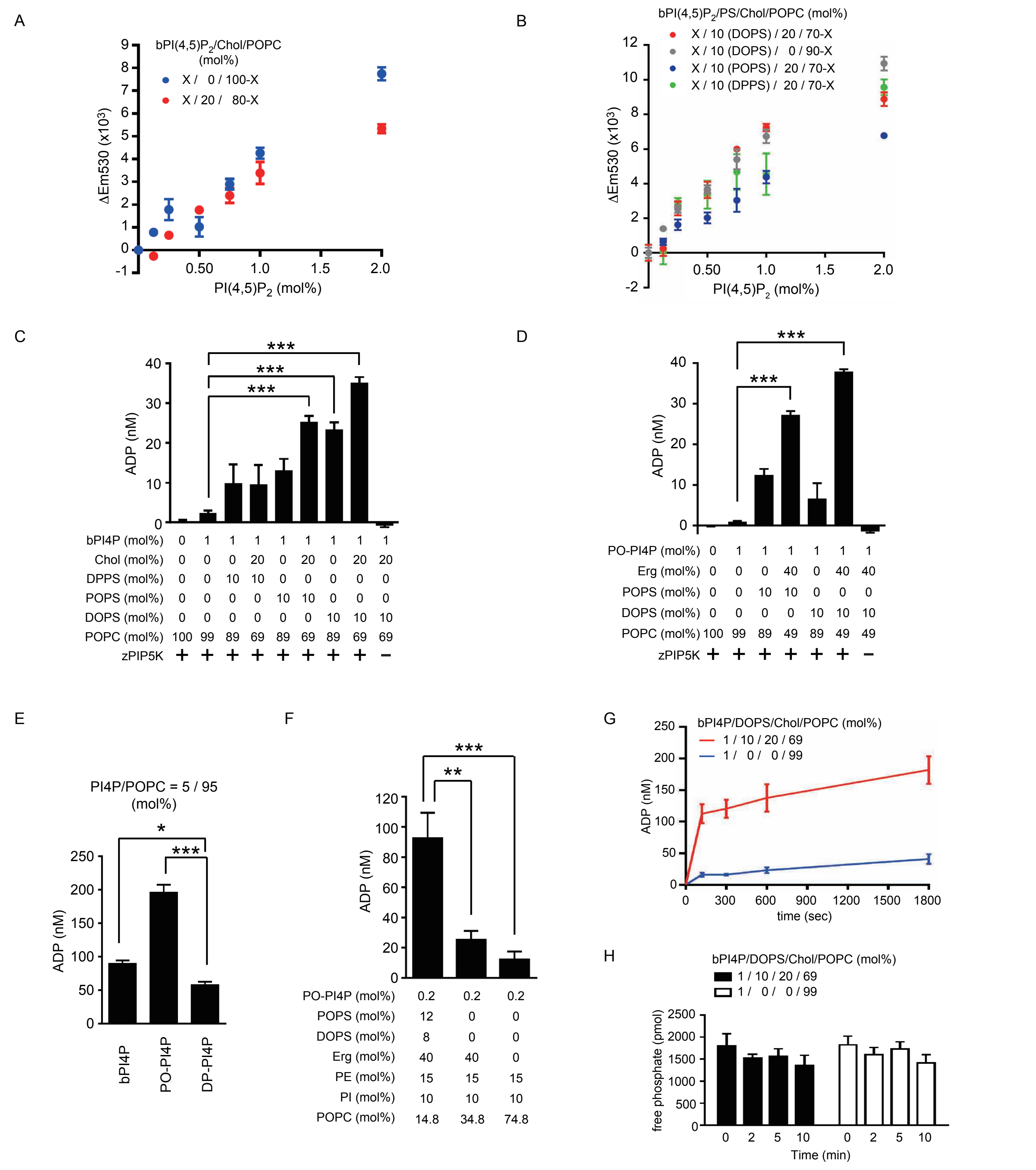

Figure S3. Sterols stimulate zPIP5K activity *in vitro,* related to Figure 3

(A) Effects of cholesterol on $PI(4,5)P_2$-dose dependency of NBD-$PH_{PLC\delta}$ fluorescence intensity. Data represent mean ± SEM (n=3).

(B) Calibration of effects by PS and cholesterol on NBD-$PH_{PLC\delta}$ sensitivity to $PI(4,5)P_2$. Data represent mean ± SEM (n=3).

(C) Stimulation of PIP5K activity by phosphatidylserine (PS) and cholesterol. Liposomes containing the indicated PS species and/or cholesterol were used as a substrate. After 3 min, the PIP5K reactions were stopped with ADP-Glo reagent. Data represent mean ± SEM (n = 3).

(D) Unsaturated PS and ergosterol (Erg) stimulate PIP5K activity. Liposomes containing various PS species and/or ergosterol as indicated were used as a substrate. After 3 min, the PIP5K reactions were stopped with ADP-Glo reagent. Data represent mean ± SEM (n = 3).

(E) The effect of PI4P unsaturation on zPIP5K activity. After 2 min, the PIP5K reactions were stopped with ADP-Glo reagent. Data represent mean ± SEM (n = 3). (F) ADP-Glo assay using PIP5K and liposomes resembling yeast PM lipid composition (Zinser et al., 1991) or in the absence of PS and ergosterol as indicated. After 2 min, the PIP5K reactions were stopped with ADP-Glo reagent. Data represent mean ± SEM (n = 3). (G) A time course analysis of PIP5K activity using the ADP-Glo assay. PIP5K (final conc. 130 nM) was used. Note that ADP levels rise suddenly after 120 sec in the presence of DOPS and cholesterol. Data represent mean ± SEM (n=3). (H) Free phosphate levels during the indicated PIP5K reactions were monitored over time by the Malachite Green Phosphate assay. Data represent mean ± SEM (n=3).
$^*P < 0.05$, $^{**}P < 0.01$, $^{***}P < 0.001$.

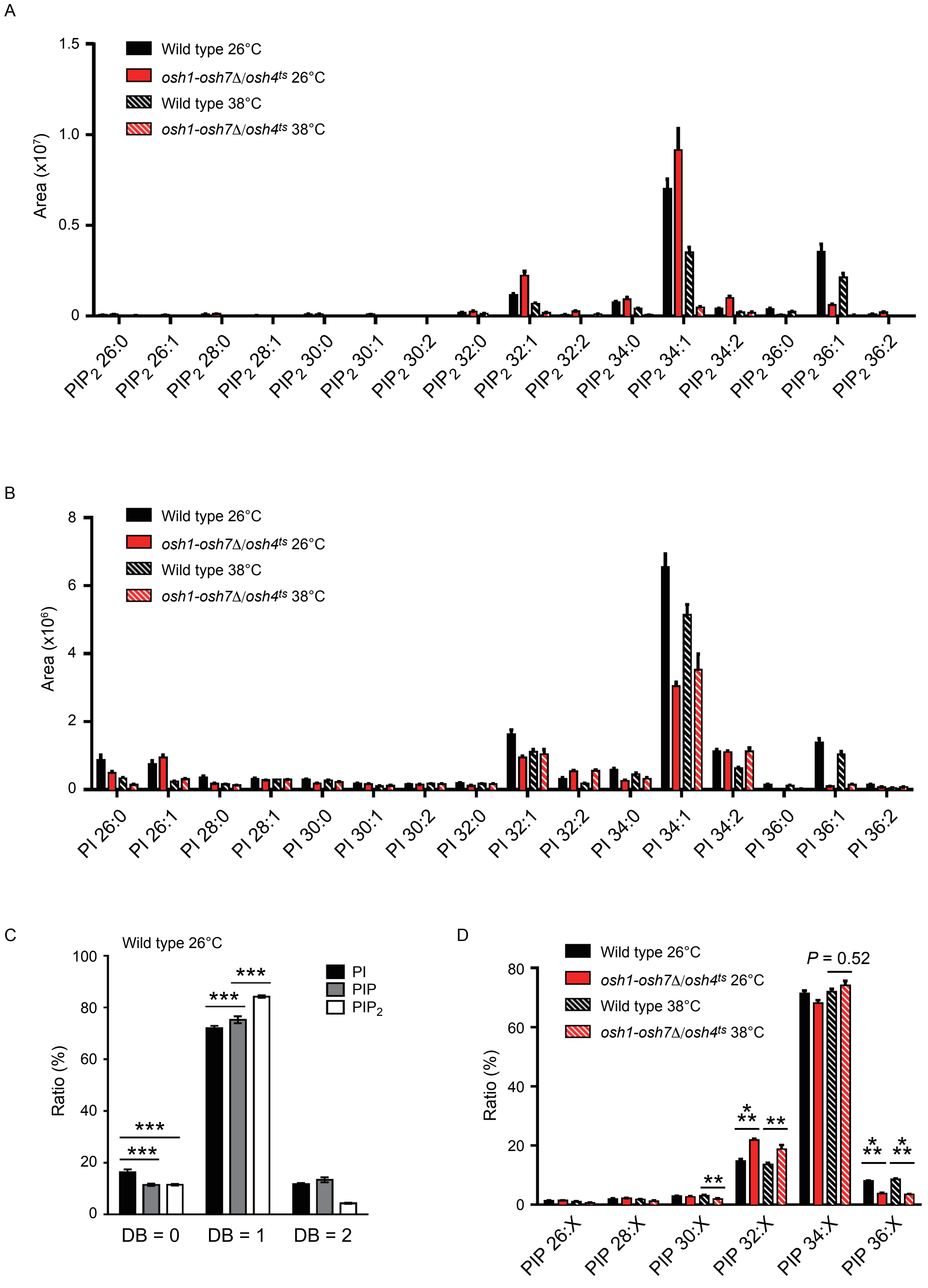

Figure S4 The fatty acid compositions of PI, PIP and PIP_2 in wild type and *osh1-7Δ/osh4^{ts}* mutant cells, related to Figure 4
Analysis of PI, PIP, and PIP_2 levels in wild-type and *osh1-7Δ/osh4^{ts}* cells cultured at 26ºC or 38ºC for 2 h by quantitative lipidomics was performed as described in the STAR Methods. (A, B) Fatty acid compositions of PIP_2 and PI. (C) Mono-unsaturated PIP_2 species are enriched in wild-type cells. (D) Fatty acid length of PIP species. (A-D) Data represent mean ± SEM (n = 5). ***P* < 0.01, ****P* < 0.001.

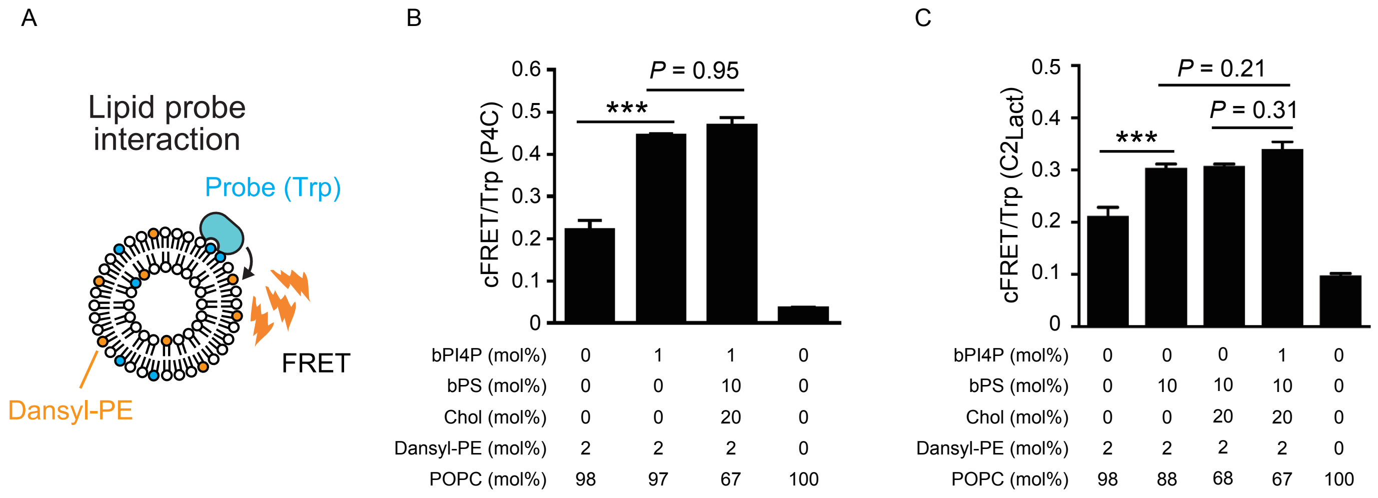

Figure S5. Quantitative measurements of lipid probe binding in the presence of unsaturated PS, PI4P, and cholesterol, related to Figure 5

(A) Membrane binding assay using Dansyl-PE liposomes. FRET signals increase by the proximity of dansyl-PE in the liposome to tryptophan residues in lipid probes. Corrected FRET (cFRET) was calculated as described in STAR Methods.

(B) Quantification of the PI4P probe (P4C) binding to dansyl-PE liposomes of defined compositions. (C) Quantification of PS probe ($C2_{Lact}$) binding to dansyl-PE liposomes as indicated. Data represent the mean ± SEM (n=3). ***$P < 0.001$.

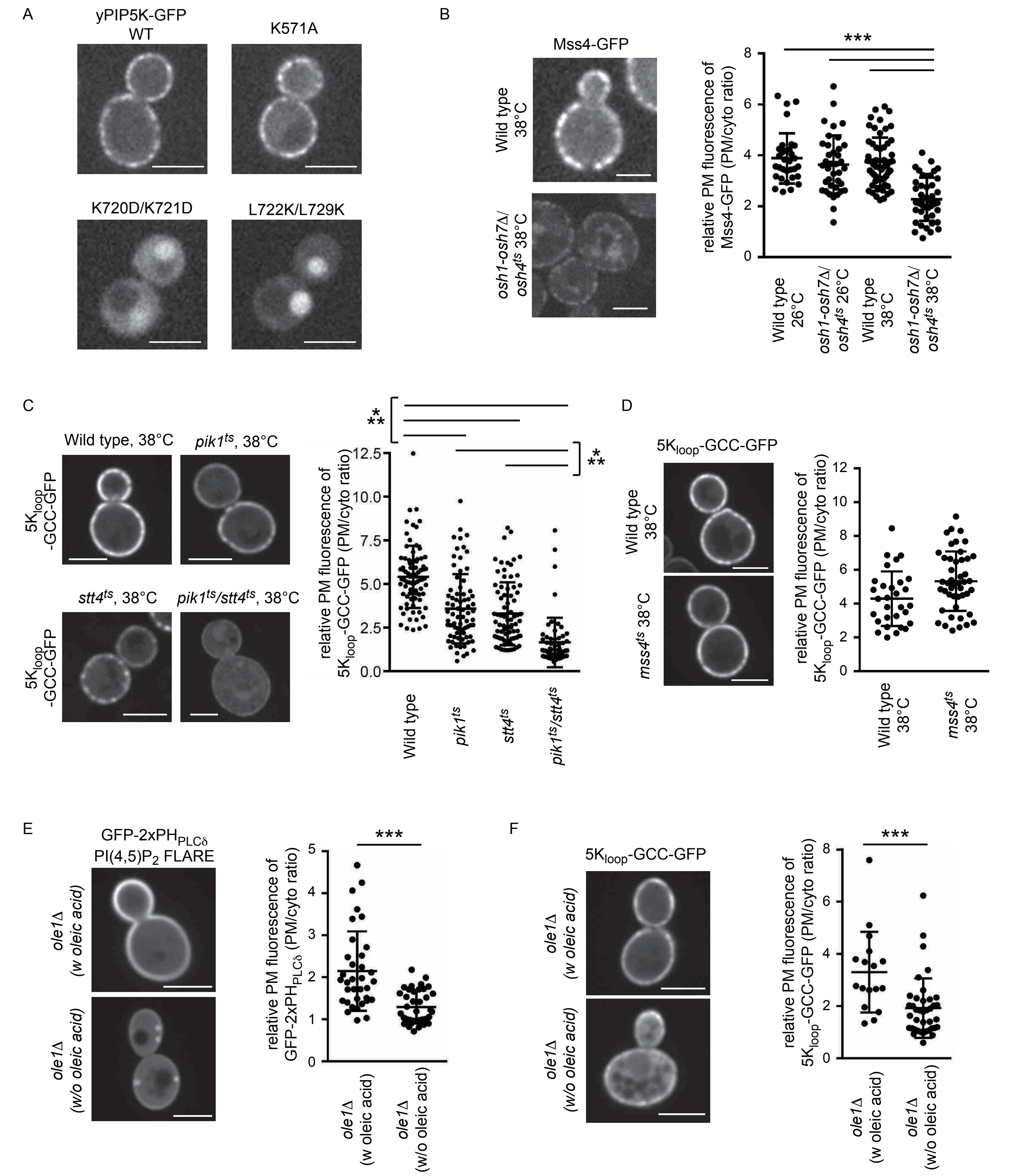

Figure S6. Unsaturated PI4P and PS co-distribution in the PM is recognized by the specificity loop of PIP5K, related to Figure 6

(A) Localization of GFP-tagged wild type and mutant yeast PIP5K Mss4 proteins (yPIP5K-GFP) in *mss4*^{ts} mutant cultured at 26ºC. (B) Mss4-GFP localization in wild type and *osh1-7*Δ/*osh4*^{ts} cells cultured at 38ºC for 2 h (left panels). Quantification of Mss4-GFP localization (PM/cyto ratio) in wild type and *osh1-7*Δ/*osh4*^{ts} cells cultured at 26ºC or 38ºC for 2 h (right panel). Data represent mean ± SD (n≧32 cells). (C) Localization of $5K_{loop}$-GCC-GFP in wild-type, *pik1*^{ts}, *stt4*^{ts} and *pik1*^{ts}/*stt4*^{ts} mutants cultured at 38ºC for 1 h. (left panels). Quantification of $5K_{loop}$-GCC-GFP signals in the PM of wild-type, *pik1*^{ts}, *stt4*^{ts} and *pik1*^{ts}/*stt4*^{ts} mutants. Data represent mean ± SD (n≧61 cells). (D) Quantitative analysis of $5K_{loop}$-GCC-GFP PM localization in wild type and *mss4*^{ts} mutant cells cultured at 38ºC for 1 h. Data represent mean ± SD (n≧30 cells). (E) Quantitative localization of GFP-$2xPH_{PLC\delta}$ in *ole1*Δ mutant cells cultured with or without 1 mM oleic acid for 20 h. Data represent mean ± SD (n≧35 cells). (F) Quantitative localization of $5K_{loop}$-GCC-GFP in *ole1*Δ mutant cells cultured with or without 1 mM oleic acid for 20 h. Data represent mean ± SD (n≧17 cells). Scale bars, 4 μm. ***$P < 0.001$.

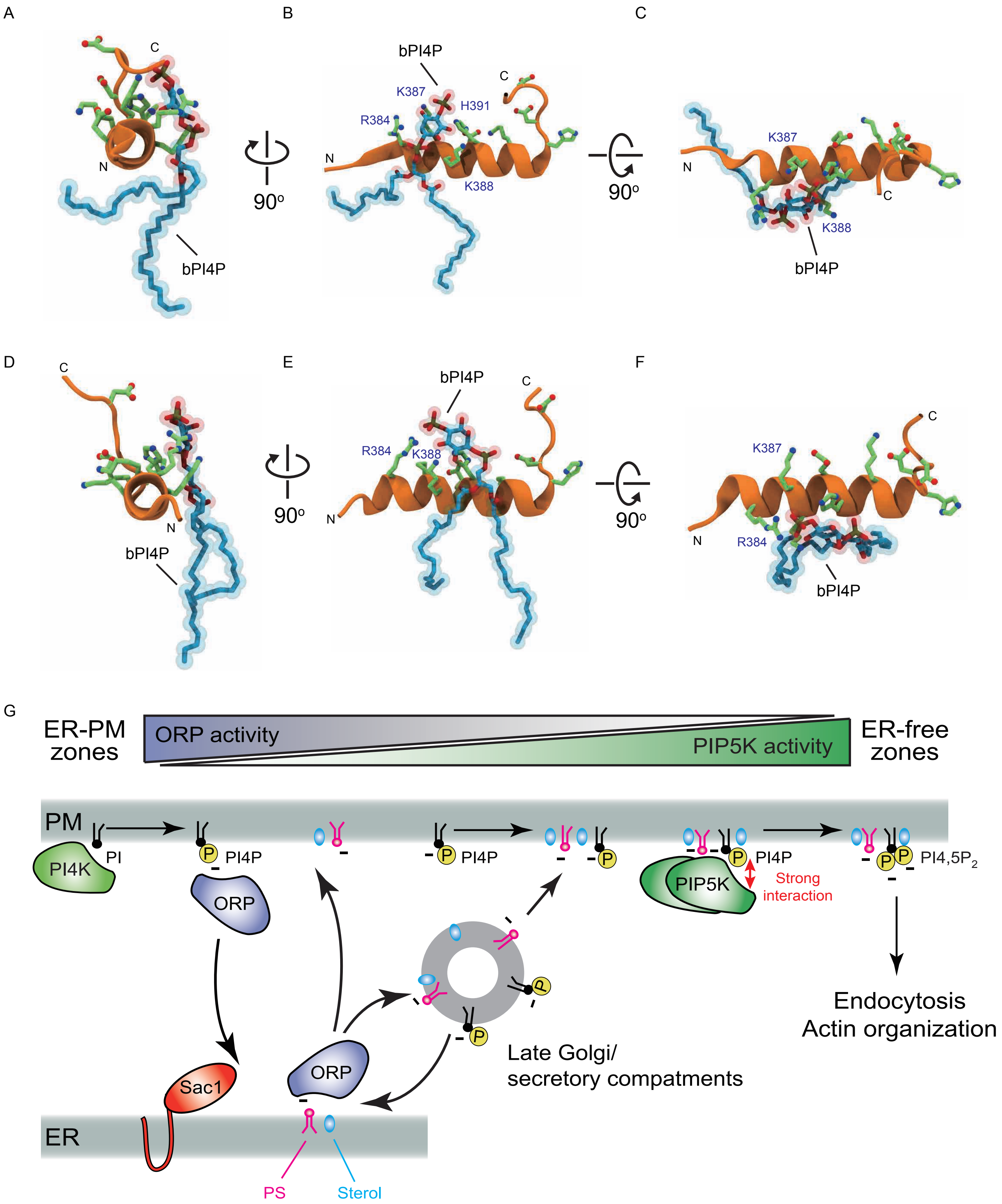

Figure S7. Speculative model for PIP5K stimulation by Osh-dependent lipid exchange, related to Figure 7
(A-F) The $5K_{loop}$ is visualized as a ribbon (orange), acidic and basic residues as green sticks, colored the same as in Fig 7H-J. (A-C) The bPI4P molecule is located at pocket #1 (Fig. 7G) and interacts with four residues of the $5K_{loop}$: R384, K387, K388, and H391. (D-F) The bPI4P molecule is located at pocket #2 (Fig. 7G) and interacts with R384 and K388. (G) Speculative model for spatial and temporal PIP5K regulation by Osh-dependent lipid transfer. PM-localized PI4KIIIα synthesizes PI4P at the PM. As PI4P alone may not be sufficient for PIP5K activity, PI4P is used for Osh-dependent lipid exchange at ER-PM zones to facilitate PS and sterol transport from the ER to the PM. Once PS and sterol lipids reach sufficient levels, PI4P utilization switches from PI4P exchange by the Osh/Sac1 system to PI(4,5)P_2 synthesis by PIP5K. In addition, PI4P generated by Golgi-localized PI4K isoforms may be used for Osh-mediated PS and sterol transport to late Golgi/secretory compartments. These PS and sterol molecules are then delivered to the PM via vesicular transport for subsequent PIP5K activity. PI(4,5)P_2 synthesized by PIP5K controls global PM organization and dynamic events including endocytosis and actin organization in ER-free PM zones.
